# Pedunculopontine Chx10^+^ neurons control global motor arrest in mice

**DOI:** 10.1038/s41593-023-01396-3

**Published:** 2023-07-27

**Authors:** Haizea Goñi-Erro, Raghavendra Selvan, Vittorio Caggiano, Roberto Leiras, Ole Kiehn

**Affiliations:** 1 Department of Neuroscience, Faculty of Health and Medical Sciences, University of Copenhagen, Copenhagen, Denmark; 2 Department of Computer Science, University of Copenhagen, Copenhagen, Denmark; 3 Department of Neuroscience, Karolinska Institutet, Stockholm, Sweden; 4 Present Address: Meta AI Research, New York, NY USA

**Keywords:** Central pattern generators, Motor control

## Abstract

Arrest of ongoing movements is an integral part of executing motor programs. Behavioral arrest may happen upon termination of a variety of goal-directed movements or as a global motor arrest either in the context of fear or in response to salient environmental cues. The neuronal circuits that bridge with the executive motor circuits to implement a global motor arrest are poorly understood. We report the discovery that the activation of glutamatergic Chx10-derived neurons in the pedunculopontine nucleus (PPN) in mice arrests all ongoing movements while simultaneously causing apnea and bradycardia. This global motor arrest has a pause-and-play pattern with an instantaneous interruption of movement followed by a short-latency continuation from where it was paused. Mice naturally perform arrest bouts with the same combination of motor and autonomic features. The Chx10-PPN-evoked arrest is different to ventrolateral periaqueductal gray-induced freezing. Our study defines a motor command that induces a global motor arrest, which may be recruited in response to salient environmental cues to allow for a preparatory or arousal state, and identifies a locomotor-opposing role for rostrally biased glutamatergic neurons in the PPN.

## Abstract (web summary)

The mechanisms underlying motor arrest are unclear. Here, the authors show that Chx10^+^ neurons in the mouse pedunculopontine nucleus control global motor arrest in a pattern of pause (movement on hold) and play (movement resuming from the exact position it paused) likely in response to salient and nonthreatening environmental cues.

## Main

The episodic nature of movement implies that its execution needs to be often arrested or interrupted. The arrest may happen when a goal is reached, for example, reaching for and grasping an object, or stopping locomotion at the desired location. Arresting goal-directed movement involves the termination of the particular motor behavior by either ceasing the initiating drive or providing active stop signals that overcome or inhibit the active drive^1–10^. In other arrest types, for example, within innate responses, the movement arrest is sudden, global and unplanned, as a reaction to threats or other salient environmental cues^5,6^. The neuronal circuits mediating defensive global motor arrest (for example, freezing) are located in the lower brainstem and are activated by neuronal pathways that include the amygdala and the ventral periaqueductal gray as core components^11–13^. However, other pathways that bridge with the executive motor circuits to implement global motor arrest outside of the defensive or fear-related contexts are not well understood.

An area where triggering of global motor arrest has been observed by broad electrical stimulation is the PPN^14,15^. The PPN is located in the upper pons/isthmus and mainly consists of glutamatergic, cholinergic and GABAergic neurons^16^. All three neuronal subtypes are present throughout the extent of the nucleus but display distinct rostrocaudal density gradients each. Glutamatergic neurons, expressing the vesicular glutamate transporter, Vglut2, are densest in the caudal part and, overall, the most abundant subtype^16–20^. The PPN is anatomically and functionally divided into a rostral part (anterior, previously pars dissipata) that receives the bulk of its inputs from the basal ganglia, in particular the substantia nigra pars reticulata, and a caudal part (posterior, pars compacta) that, among others, receives broad somatosensory inputs^21,22^. Reflecting the neurochemical and input/output complexity, the PPN has been implicated in different physiological functions ranging from pure motor functions to, among others, autonomic functions, arousal or attention^20,23–25^.

Optogenetic or chemogenetic activation of caudal glutamatergic PPN neurons in rodents leads to the initiation of slow explorative locomotion^26–30^. However, lack of locomotion initiation, mixed responses or movement arrest have also been reported after broad activation of glutamatergic PPN neurons^29,31,32^, hinting toward a functional heterogeneity. We hypothesized that rostral versus caudal glutamatergic PPN neurons may control diverse or even opposing motor outputs, which could be carried out by molecularly distinct subpopulations of glutamatergic neurons.

Using a combination of anatomical, physiological, and behavioral approaches, we reveal the presence of a rostrally biased glutamatergic subpopulation in the PPN that is characterized by the expression of the transcription factor Chx10, and whose activation causes an arrest of all ongoing motor activity together with apnea and bradycardia. This transient global motor arrest is characterized by a pause-and-play pattern, is different to previously described arrest types like freezing, and may be recruited in response to salient environmental stimuli to allow for a preparatory or arousal state.

## Results

### A subpopulation of glutamatergic pedunculopontine nucleus neurons is Chx10^+^

**Fig. 1:**
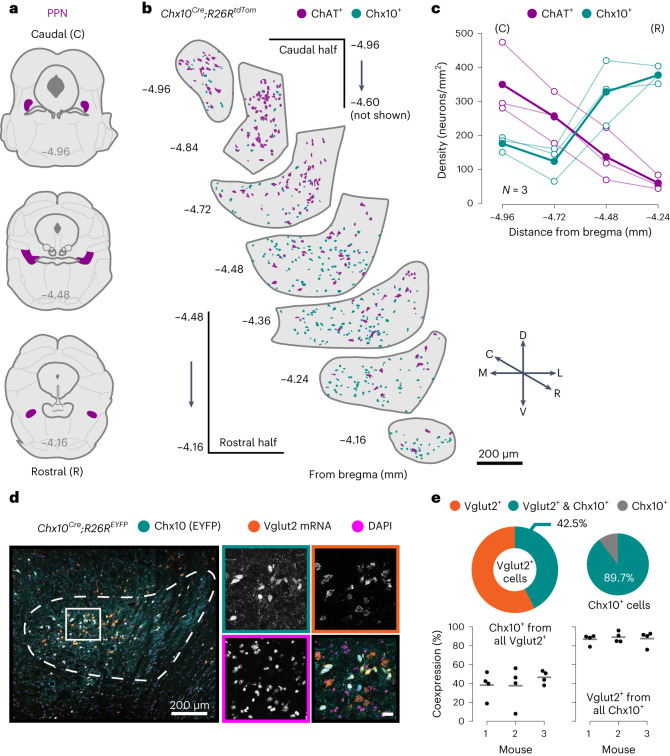
Chx10-derived neurons define a subpopulation of glutamatergic PPN neurons with a rostral bias. **a**, Coronal plane schematics highlighting the PPN (magenta) at different levels of its rostrocaudal axis. From bregma: caudal edge at −4.96 mm (top), rostral edge at −4.16 mm (bottom). **b**, Spatial distribution of Chx10^+^ (cyan, *Chx10*^*Cre*^; *R26R*^*tdTomato*^ reporter mouse) and ChAT^+^ (magenta, antibody) neurons along the rostrocaudal axis of the PPN. C, caudal; R, rostral; D, dorsal; V, ventral; M, medial; L, lateral. **c**, Quantification of Chx10^+^ (cyan) and ChAT^+^ (magenta) neuron densities at four coronal levels of the PPN: −4.96 and −4.72 mm from the caudal half, −4.48 and −4.24 mm from the rostral half. For each cell type, hollow circles are individual mice and filled circles the group average (*N* = 3 mice, 1 hemisection/level/mouse). **d**, Left, confocal photomicrograph of the rostral PPN from a *Chx10*^*Cre*^; *R26R*^*EYFP*^ reporter mouse that labels all Chx10^+^ neurons (Chx10, cyan), in situ hybridization for Vglut2 mRNA (Vglut2 mRNA, orange) and nuclear staining (DAPI, pink). The dashed line delineates the PPN. The solid square delineates the magnified area. Right, single-channel images (grayscale) of the magnified area framed following the same color code, and the composite image (colored) at the bottom right. Scale bar, 20 µm. **e**, Percentage of neurons coexpressing Chx10 and Vglut2 within the PPN (from all Vglut2^+^, left; from all Chx10^+^, right). Most Chx10^+^ neurons also express Vglut2 mRNA. Pie charts depict group means (Vglut2^+^only, orange; Vglut2^+^ and Chx10^+^, cyan; Chx10^+^ only, gray). Strip plots show single hemisections and the mouse average (gray line; *N* = 3 mice, 4 hemisections/mouse). Source data

The PPN is an elongated nucleus along the rostrocaudal axis (Fig. 1a)^33^ anatomically subdivided into a rostral and a caudal part^20,23,25^. To evaluate if excitatory neurons are further diversified into molecularly defined rostral versus caudal PPN subpopulations, we focused on the transcription factor Chx10 (also known as Vsx2) because it has been previously found in motor-related glutamatergic neurons of the mouse brainstem and spinal cord^7,34,35^. In an initial exploratory search, we found that Chx10^+^ neurons are also present in the PPN (hereafter, ‘Chx10-PPN’).

**Extended Data Fig. 1:**
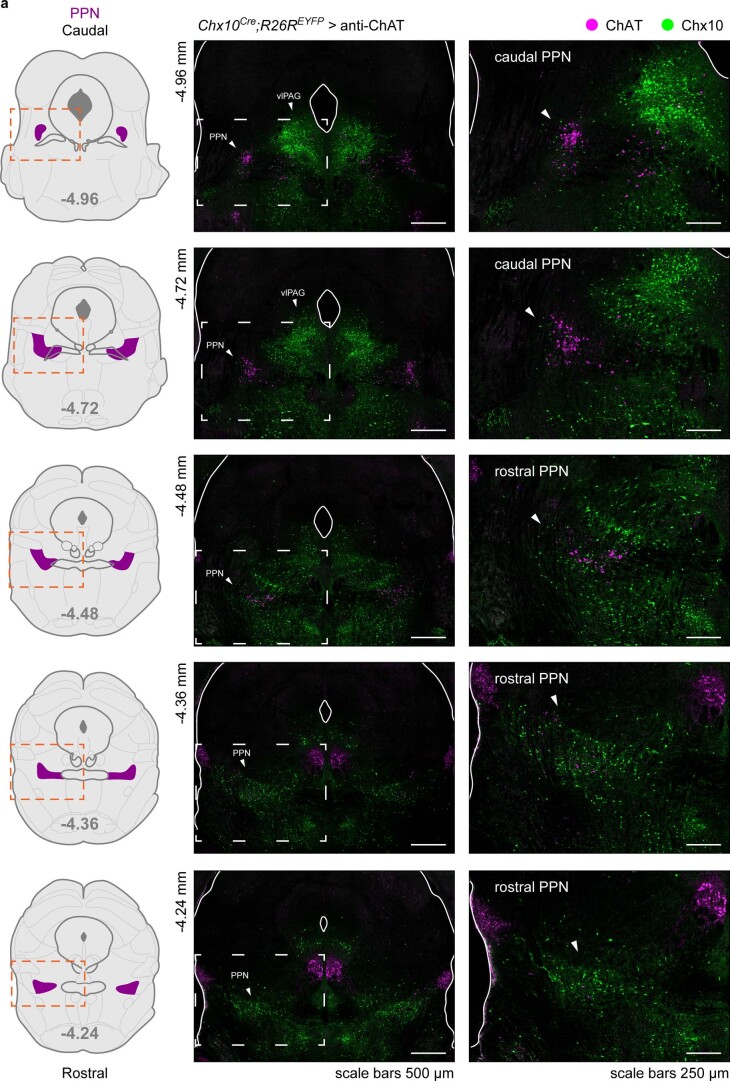
Mutually exclusive distribution of Chx10^+^ and ChAT^+^ neurons along the rostro-caudal axis of the PPN. **a**, Left, coronal plane schematics highlighting the PPN (magenta) at different rostro-caudal levels. Right, confocal photomicrographs showing the distribution of Chx10^+^ (green, *Chx10*^*Cre*^; *R26*^*EYFP*^ reporter mouse) and ChAT^+^ (magenta, antibody) neurons at the corresponding rostro-caudal levels of the schematics to the left. The pictures at the right-most column are magnified images corresponding to the area delineated by dashed lines in the pictures to the left. The same area is also delineated in the schematics with orange dashed lines. White arrowheads in the magnified pictures point to the PPN. Chx10^+^ neurons are sparse within the caudal half of the PPN while their density increases in the rostral half of the PPN, following an inverted pattern compared to ChAT^+^ neurons within the nucleus. There is no overlap in the cellular expression of ChAT and Chx10 at any rostro-caudal level of the PPN. Chx10^+^ neurons are also found in the vlPAG where they are enriched at the coronal levels corresponding to the caudal half of the PPN (see also Extended Data Fig. 8).

To assess the distribution of Chx10^+^ neurons in the PPN, we crossed *Chx10*^*Cre*^ mice with the *R26R*^*tdTomato*^ conditional reporter line to permanently label Chx10^+^ neurons^7,36^ and simultaneously visualized cholinergic cells by immunostaining against choline acetyltransferase (ChAT). This approach allowed us to characterize Chx10^+^ neuron distribution through the entire rostrocaudal extent of the PPN using the cholinergic neurons as reference. We observed a gradient, with Chx10^+^ neurons preferentially located in the rostral half of the nucleus (bregma −4.48 to −4.16 mm) compared to the caudal half (bregma −4.96 to −4.72 mm; −4.60 mm not shown); while ChAT^+^ neurons, in agreement with previous reports^17^, were densely packed in the caudal half but sparsely distributed in the rostral half (Fig. 1b and Extended Data Fig. 1). We then quantified neuron density at four different coronal levels (Fig. 1c), confirming the nonuniform distribution of Chx10^+^ neurons along the rostrocaudal axis of the PPN (Fig. 1c; one-way repeated measures (RM) analysis of variance (ANOVA), *F*_(1.379, 2.758)_ = 24.35, *P* = 0.0185) and a higher density in the rostral half compared to the caudal half (Tukey’s multiple-comparisons test, adjusted *P* values: −4.96 versus −4.24, *P* = 0.0280; −4.72 versus −4.48 *P* = 0.0470; −4.72 versus −4.24, *P* = 0.0356; all other comparisons not significant (NS)). On average, the lowest density of Chx10^+^ neurons was at bregma −4.72 mm (123.27 ± 51.44 neurons/mm^2^), and the highest at bregma −4.24 mm (377.07 ± 26.05 neurons/mm^2^; mean ± s.d.). As expected^17^, ChAT^+^ neuron density was also different along the rostrocaudal axis (Fig. 1c; one-way RM ANOVA, *F*_(1.420, 2.839)_ = 31.12, *P* = 0.0124), with a progressive reduction from caudal to rostral of nearly 100 neurons/mm^2^ (test for linear trend, slope = −98.89, confidence interval (CI) = −73.78 to −124.0), from 349.44 ± 108.09 neurons/mm^2^ at bregma −4.96 mm to 59.14 ± 21.08 neurons/mm^2^ at bregma −4.24 mm (mean ± s.d.).

We then investigated the neurotransmitter phenotype of Chx10^+^ neurons in the PPN. We did not detect any overlap of Chx10 with ChAT in our initial experiments (Fig. 1b and Extended Data Fig. 1), and because Chx10^+^ neurons are glutamatergic and Vglut2^+^ in other mouse brainstem areas^7,37^ and the spinal cord^38,39^, we focused on Vglut2. We performed in situ hybridization for Vglut2 mRNA using RNAscope combined with immunostaining against eYFP in sections of *Chx10*^*Cre*^; *R26R*^*EYFP*^ reporter mice, which express eYFP in all Chx10^+^ neurons, and quantified their coexpression (Fig. 1d). Most Chx10^+^ cells expressed Vglut2 mRNA (89.67% ± 0.75%, mean ± s.d.; Fig. 1e). Although we did not analyze other neurotransmitter markers than Vglut2, it is unlikely that Vglut2^+^/Chx10^+^ neurons in the PPN also coexpress ChAT or GABA, because previous reports in adult rodents show only minor coexpression between ChAT and Vglut2 (refs. ^16,40,41^) and no overlap between Vglut2 and GAD65/GAD67 (ref. ^40^). Thus, we conclude that Chx10-PPN neurons are glutamatergic. In addition, we also quantified the proportion of Chx10^+^ neurons among all detected Vglut2^+^ neurons (Fig. 1e). Coexpression levels varied widely from 7.91% to 56.14%, likely depending on the rostrocaudal level, although this was not formally addressed. On average, 42.5% ± 3.34% (mean ± s.d.) of all quantified Vglut2^+^ neurons also coexpressed Chx10.

In sum, Chx10-expressing neurons constitute a subpopulation of the Vglut2-expressing glutamatergic neurons in the PPN and are predominantly located in the rostral half of the nucleus.

### Activation of Chx10-PPN neurons arrests movement

**Fig. 2:**
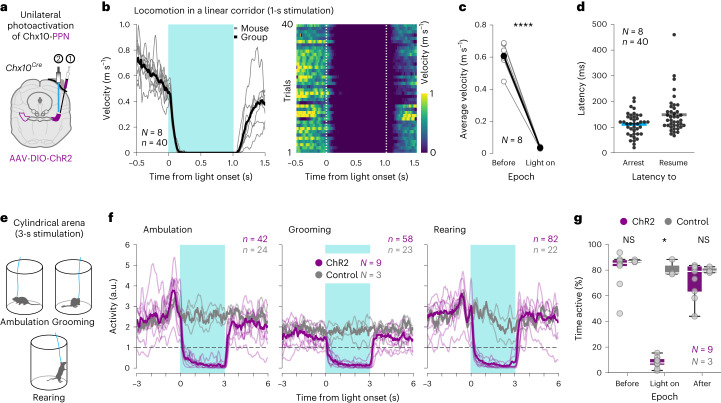
Activation of Chx10-PPN neurons causes global motor arrest. **a**, Experimental strategy to optogenetically target Chx10-PPN neurons in *Chx10*^*Cre*^ mice. **b**, Effect of Chx10-PPN neuron activation on velocity during locomotion in a linear corridor. Left, group average velocity (black line; 40 trials) and mouse average velocities (gray lines; *N* = 8 mice, 5 trials/mouse). Right, velocity heat map for individual trials. Dashed vertical lines delimit light onset and offset. **c**, Average locomotor velocities while crossing the linear corridor before light onset (before) and during blue-light stimulation (light on; two-tailed paired *t*-test; light on versus before, mean speed difference = −0.57 ± 0.07 m s^−1^ (s.d.), CI = −0.63 to −0.51, *P* *<* 0.0001). Gray hollow circles are individual mice (mouse average), and black filled circles indicate the group average (*N* *=* 8 mice, 5 trials/mouse). **d**, Latencies to arrest locomotion from light onset (left, blue), and latencies to resume locomotion from light offset (right, gray). Dots are individual trials, and lines represent group means. **e**, Setup to assess the effect of Chx10-PPN neuron activation during slow ambulation, grooming and rearing. **f**, Arrest of all assessed behaviors during light stimulation in mice expressing ChR2 in Chx10-PPN neurons, while EYFP-expressing control mice are unaffected. For each treatment group (ChR2, magenta, *N* *=* 9 mice; control (EYFP), gray, *N* *=* 3 mice), thick lines are the group average activity and thin lines indicate the mouse average activity. Horizontal dashed lines define the inactivity threshold. Blue shades (**b** and **f**) delimit light stimulus duration. **g**, Percentage of time that mice spent being active within each of the 3-s epochs around stimulation (before, light on, after) in the cylinder test, all three behaviors combined. Mice in the ChR2-expressing group (magenta, *N* = 9) are inactive during stimulation, while mice in the EYFP-expressing control group (gray, *N* *=* 3) remain active (Mann–Whitney test with Holm–Sidak’s correction for multiple comparisons, ChR2 versus control, adjusted *P* values: light on, *P* *=* 0.027026; before and after NS). In box-and-whisker plots, white lines indicate medians, box edges the IQR, and whiskers extend to the minimum (Q1 – 1.5 × IQR) and maximum (Q3 + 1.5 × IQR). Circles represent individual mice. a.u., arbitrary units. Source data

**Extended Data Fig. 2:**
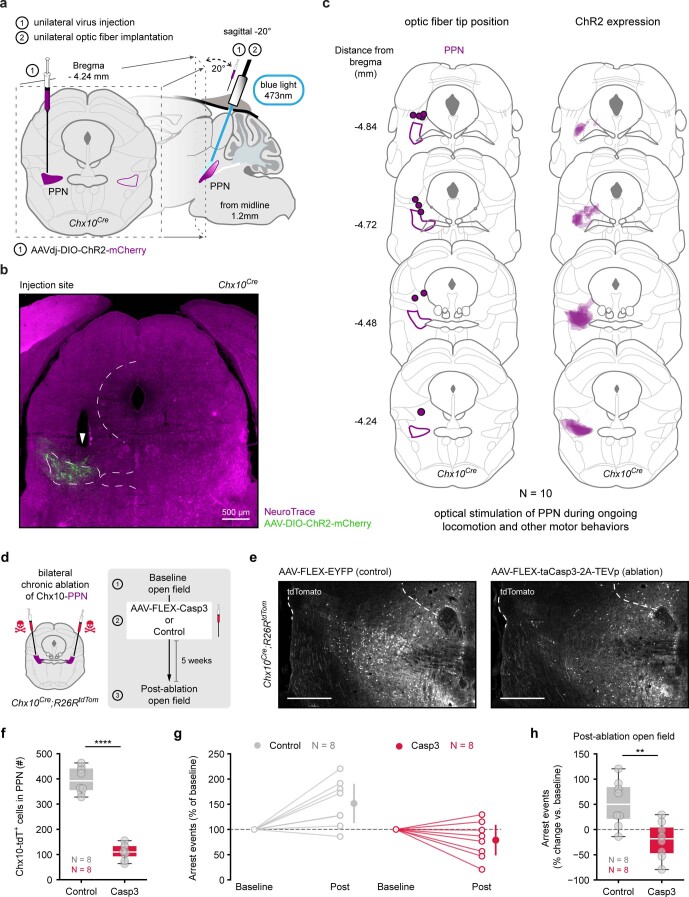
Experimental approach to target Chx10-PPN neurons during optogenetic activation and ablation experiments. **a**, Extended overview of the experimental strategy to optogenetically target Chx10^+^ neurons in the PPN using a Cre-dependent viral approach in *Chx10*^*Cre*^ mice. **b**, Example photomicrograph (coronal plane) of the rostral PPN at the injection site with the ChR2-mCherry-expressing Chx10-PPN neurons in green and a fluorescent Nissl stain (NeuroTrace) in violet. White arrowhead points to the tip of the optic fiber. **c-d**, Reconstruction of the optic fiber tip position (**c**) and ChR2 expression (**d**) for all mice (N = 10) used in the optogenetic experiments performed to assess the effect of Chx10-PPN activation during ongoing locomotion (linear corridor; N = 8) and during other motor behaviors (ambulation, grooming, rearing; cylinder test; N = 9). **d**, Experimental strategy to assess the effect of Chx10-PPN neuron ablation over naturally occurring arrest events using a Cre-dependent Caspase3-based viral approach in *Chx10*^*Cre*^; *R26R*^*tdTomato*^ mice. **e**, Example fluorescence photomicrographs of the PPN from *Chx10*^*Cre*^; *R26R*^*tdTomato*^ reporter mice with Chx10^+^ neurons expressing tdTomato (white), acquired after the post-ablation open field time point. Left, PPN of an EYFP-injected control mouse; right, PPN of a Casp3-injected mouse. For anatomical reference, in both images the dashed lines delimit the lateral border of the parabigeminal nucleus (left) and the ventral border of the periaqueductal gray (right). Scale bars, 500 µm. **f**, Number of Chx10-tdTomato^+^ cells in the PPN of Control (left, gray) and Casp3 (right, red) mice (N = 8 mice per group; bilateral counts, one section/mouse) (two-tailed unpaired *t*-test, Control vs. Casp3: difference between means = 286.72 cells, CI = 239.3 to 117.66 cells, *P* < 0.0001). **g**, Arrest events in the open field during baseline and post-injection open field sessions in control mice (left, gray; N = 8) and Casp3-injected mice (right, red; N = 8), displayed as the % of baseline for each mouse. Hollow circles are individual mice, solid circles and error line are the mean and CI. **h**, Arrest events in the post-injection open field session displayed as the % change from baseline for each mouse (two-tailed unpaired *t*-test, Control vs. Casp3: difference between means = 72.72 %, CI = 27.84 to 117.66 %, *P* = 0.0039). In box-and-whisker plots, white lines indicate medians, box edges the interquartile range (IQR), and whiskers extend to the minimum and maximum. Circles represent individual mice.

We next investigated whether Chx10-PPN neurons play a role in motor control using an optogenetic approach. *Chx10*^*Cre*^ mice were unilaterally injected in the PPN with either a Cre-dependent channelrhodopsin virus (AAV-DIO-ChR2) or a control virus only expressing a fluorophore (AAV-DIO-eYFP, ‘control’), and then implanted with an optic fiber. The injection and implantation had a −20° angle in the sagittal plane pointing toward the rostral pole of the PPN. The end position of the fiber tip was ~400–500 µm dorsal to the injection center (along the dorsoventral axis) but following the same trajectory as the injection tract, overall favoring the light activation of the rostral PPN (Fig. 2a and Extended Data Fig. 2a–c).

Mice were tested in two different environments: a linear corridor to assess the effect on locomotion, and a cylindrical arena, where mice more frequently express other motor programs than straight locomotion such as grooming and rearing.

In the linear corridor, unilateral photoactivation (blue light, 40 Hz, 1 s) of Chx10-PPN neurons reliably and abruptly halted locomotion in every trial from all mice (*N* = 8 mice, 5 trials/mouse; Fig. 2b,c and Supplementary Video 1), from an average speed of 0.61 ± 0.07 m s^−1^ before light to an average of 0.03 ± 0.01 m s^−1^ during the light-on epoch, which includes the latency until full arrest (mean ± s.d.; two-tailed paired *t*-test, light on versus before, mean speed difference = −0.57 ± 0.07 m s^−1^ (s.d.), CI = −0.63 to −0.51, *P* < 0.0001). The locomotor arrest was evoked in every trial (40/40; Fig. 2b) with an average latency of 110.83 ± 44.01 ms from light onset (Fig. 2d), and lasted through the entire stimulus (average time immobile = 1.04 ± 0.1 s; mean ± s.d.). Mice resumed locomotion 148.33 ± 73.66 ms (mean ± s.d.) after light offset (Fig. 2d). Thus, locomotor arrest is reliably evoked by unilateral activation of Chx10-PPN neurons with an almost instantaneous arrest and fast recovery upon stimulation.

In the cylinder, optogenetic stimulation (blue light, 40 Hz, 3 s) was triggered when mice initiated any of the three selected behaviors: ambulation, grooming or rearing (Fig. 2e). Regardless of behavior type, ChR2-expressing mice temporarily paused their actions during stimulation (Fig. 2f, *N* = 9; Supplementary Video 2), while control mice remained active throughout (Fig. 2f, *N* = 3). The activity pattern before light onset varied between behavior types reflecting their different dynamics: a single peak when initiating ambulation, a relatively flat line during grooming, and a double peak at the beginning of a rearing bout (Fig. 2f). While these shapes were comparable between ChR2 and control, light stimulation led to a sharp activity decrease only in ChR2-expressing mice. To compare the ChR2 and control groups, we looked at the percentage of time that mice spent being active (above the inactivity threshold; Fig. 2f), where each 3-s-long epoch (before, light on, after) corresponds to 100% of the time (Fig. 2g). ChR2-expressing mice and control mice spent, on average, a similar amount of time active before and after light (ChR2 versus control: 80.59% ± 14.47% versus 87.01% ± 0.94% before, and 71.98% ± 13.83% versus 79.92% ± 2.39% after, mean ± s.d.). However, during light, the ChR2 group became inactive while control mice remained active (ChR2 versus control: 8.17% ± 4.27% versus 81% ± 6.34% of time active, mean ± s.d.; mean difference during light on = −72.83%, CI = −94.34% to −51.32%; multiple Mann–Whitney tests; correction for multiple comparisons with Holm–Sidak’s method, ChR2 versus control, adjusted *P* values: light on, *P* = 0.027026; before and after NS). Because the activity in both groups only differed during light stimulation regardless of behavior type, we conclude that the movement arrest is due to the activation of Chx10-PPN neurons.

To evaluate if Chx10-PPN neurons play a role in the expression of naturally occurring arrest events, we bilaterally ablated Chx10-PPN neurons using a Cre-dependent Caspase3-based viral strategy that triggers cell-autonomous apoptosis^42,43^. We first recorded 16 naïve *Chx10*^*Cre*^; *R26R*^*tdTomato*^ mice in an open field (OF) arena (baseline OF) and quantified the total number of naturally occurring arrest events (arrest bouts between 500 ms and 2 s long). Mice were then bilaterally injected in the PPN with either AAV5-FLEX-taCasp3-2A-TEVp (Casp3 group) or AAV5-FLEX-EYFP (control group; *N* = 8 mice each). Five weeks after surgery, both groups were again exposed to the OF (post-injection OF; Extended Data Fig. 2d). The number of Chx10-PPN cells was significantly lower in the Casp3 group compared to control mice (Extended Data Fig. 2e,f). Control mice typically increased (7/8 mice) the number of arrest events in the 5 weeks post-injection OF session compared to their baseline session (control, post-injection versus baseline: mean difference = 51.59%; minimum, maximum = 12.87%, 90.31%; Extended Data Fig. 2g). In contrast, Casp3-injected mice typically decreased (6/8 mice) the number of arrest events performed during the post-ablation OF compared to their baseline session (Casp3, post-ablation versus baseline: mean difference = −21.13%; min, max = −79.25%, 29.41%; Extended Data Fig. 2g). The decrease in arrest events by the Casp3 group was significantly different to the increase showed by the control group (percentage change from baseline, two-tailed unpaired *t*-test, control versus Casp3: difference between means = 72.72%, CI = 27.84% to 117.66%, *P* = 0.0039; Extended Data Fig. 2h), suggesting that Chx10-PPN neurons regulate the expression of natural arrest events.

These findings demonstrate that Chx10-PPN neurons play a role in motor control as their activation reliably leads to global motor arrest, while their ablation reduces the amount of naturally occurring arrest events.

### Chx10-PPN neurons hold movement with a pause-and-play pattern

**Fig. 3:**
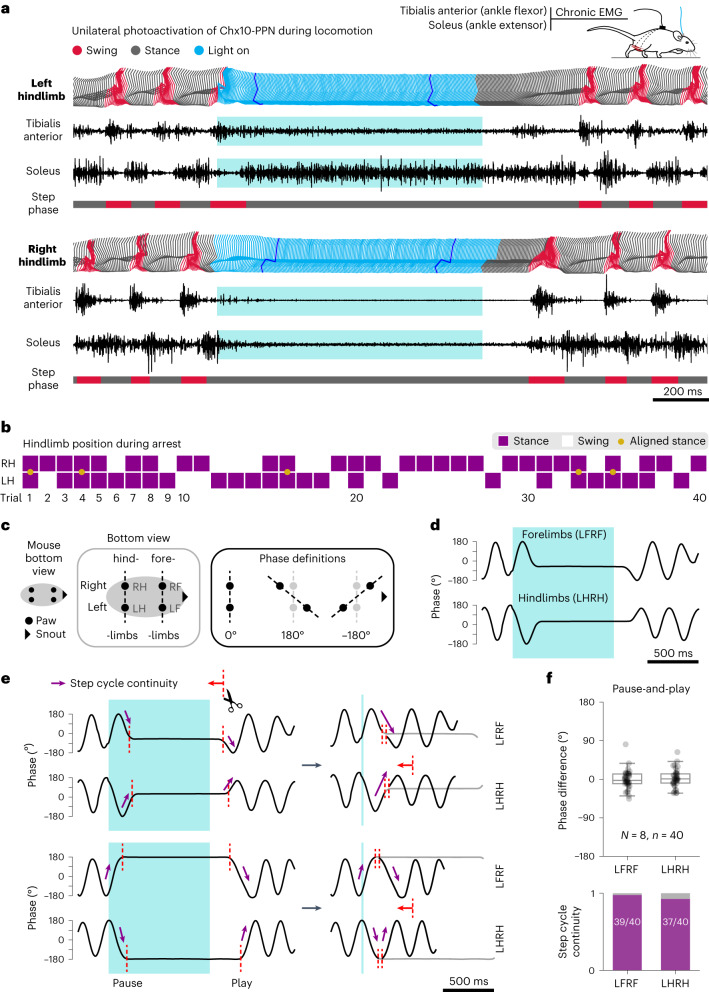
The Chx10-PPN-evoked motor arrest has a pause-and-play pattern without a distinct kinematic signature. **a**, Hindlimb dynamics of a *Chx10*^*Cre*^ mouse expressing ChR2 in the PPN during a locomotor bout in the linear corridor temporarily interrupted by activation of Chx10-PPN neurons. Left and right hindlimb activity represented with three methods: top, stick diagrams to track joint positions over time; middle, EMG recordings showing the activity of ankle flexor (tibialis anterior) and ankle extensor (soleus) muscles; bottom, step-phase classification into stance (gray) and swing (red) phases. Stick diagrams follow the same color code, except during the light-on period (light blue). Dark-blue sticks highlight how joints remain in the same position throughout the stimulation once an arrest position has been reached. **b**, Hindlimb (RH, right; LH, left) position during arrest. Solid magenta squares indicate stance phase, while empty squares indicate swing phase. Yellow dots indicate that the animal had both legs on the ground aligned perpendicular to the body axis (*n* *=* 40 trials, *N* *=* 8 mice). **c**, Explanatory diagrams of the limb coordination tracking based on paw positions (bottom view). Phase values are defined by the distance on the *x* axis between left and right forelimbs or hindlimbs. **d**, Representative example of phase values for LFRF and LHRH during locomotion in a Chx10-PPN stimulation trial. **e**, Representative stimulation trials illustrating step cycle continuity after light offset, both when the arrest happens midway (top example, rising or falling phase), or at maximum left–right displacement (bottom example, peak or trough). **f**, Quantification of the pause-and-play pattern. Top, phase difference between pause-and-play time points for forelimbs (left) and hindlimbs (right). Bottom, step cycle continuity as illustrated by purple arrows in **e**, quantified as a binary outcome for all trials. In box-and-whisker plots, central lines indicate medians, box edges the IQR, and whiskers extend to the minimum (Q1 – 1.5 × IQR) and maximum (Q3 + 1.5 × IQR). Circles represent individual trials (*n* *=* 40 trials, *N* *=* 8 mice, 5 trials/mouse). Blue shades (**a**, **c** and **d**) delimit light stimulus duration. Source data

A question that arises from the light-evoked global motor arrest is whether Chx10-PPN activation acts as a command that results in a specific and consistently evoked posture and/or muscle activation pattern^7^, or it causes an arrest with no associated stereotypic pattern. We used several complementary approaches to assess if any characteristic pattern emerged when Chx10-PPN neurons were activated during locomotion (Fig. 3).

**Extended Data Fig. 3:**
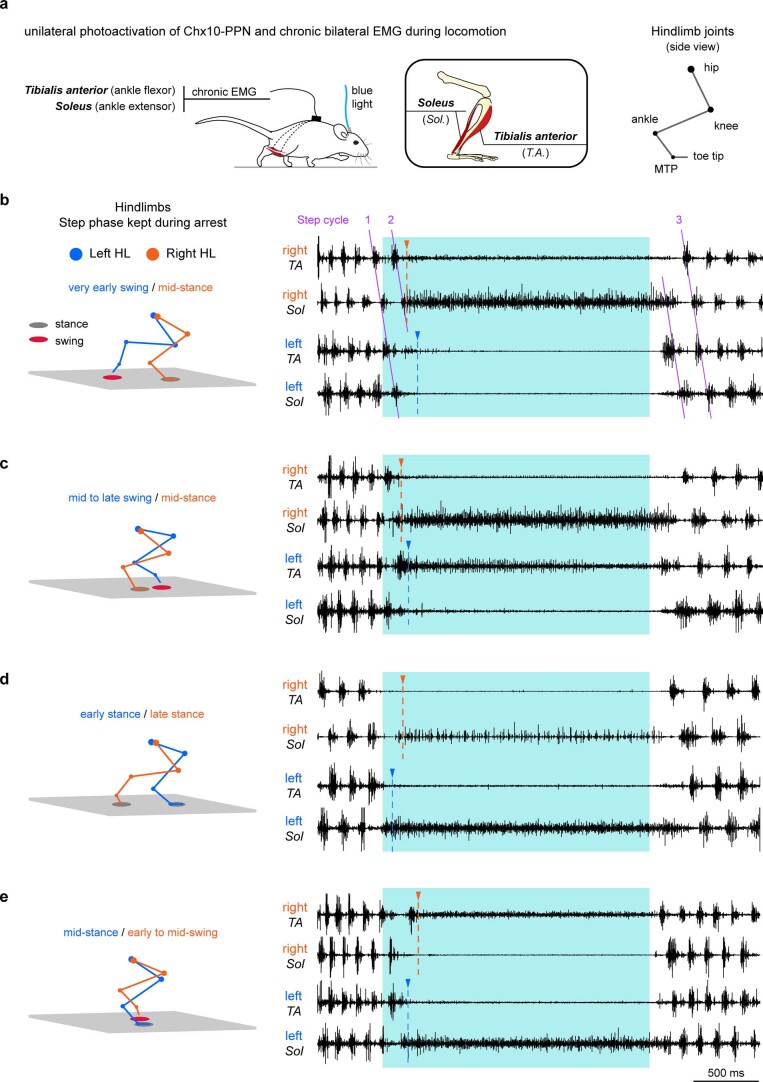
Stimulation of Chx10-PPN neurons at different phases of the step cycle leads to unique arrest patterns. **a**, Left, summary of the experimental setup for chronic EMG recordings during locomotion in a linear corridor paired with optogenetic stimulation of Chx10-PPN neurons. Center, drawing of the recorded hindlimb muscles. Right, hindlimb joint diagram (side-view) used to model limb and joint positions. MTP, metatarsophalangeal joint. **b-e**, Examples from a single animal of unique arrest patterns at different phases of the step cycle. For all panels: Left, stick diagrams showing hindlimb joint positions during arrest for the same animal stimulated at four different time-points within the step cycle resulting in unique step phase combinations kept during arrest (blue, left hindlimb; orange, right hindlimb). The elliptical shade below each foot indicates the step cycle phase (gray, stance; red, swing). Right, EMG recordings showing the muscle activity of the right *Tibialis anterior* (right *TA*, ankle flexor), the right *Soleus* (right *Sol*, ankle extensor), the left *TA*, and the left *Sol*; before, during and after light stimulus for the four different trials. The unique combination of muscle activity during light on is the one supporting the hindlimb position kept during arrest, which is depicted to the left with stick diagrams. Arrowheads and dashed lines mark the moment of movement arrest for each of the limbs (right limb, orange; left limb, blue). Purple lines in **b** (right) mark the pattern of muscle activation during three consecutive step cycles (denoted as 1, 2, 3). Notice how the pattern in cycle no. 2 is kept on hold during the light-evoked arrest, and the sequence of muscle activation is resumed after light off. Blue shades delimit light stimulus duration.

**Extended Data Fig. 4:**
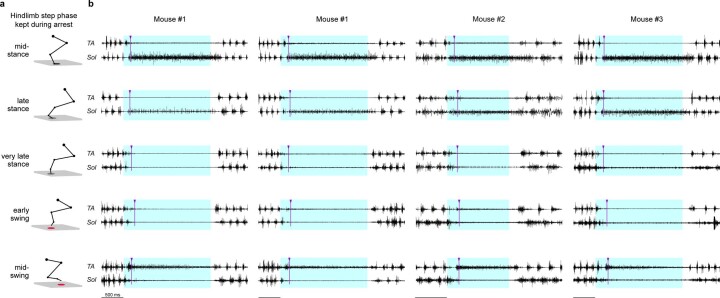
Stimulation of Chx10-PPN neurons at different phases of the step cycle leads to unique arrest patterns that are reproducible across animals. Chronic hindlimb EMG recordings during locomotion in a linear corridor paired with optogenetic stimulation of Chx10-PPN neurons. **a**, Hindlimb step phase kept during the optogenetically evoked locomotor arrest depicted by stick diagrams. The elliptical shade below the foot indicates the step cycle phase (gray, stance; red, swing). **b**, Matrix showing examples of muscle activity patterns when the same animal (first and second columns, mouse #1) or different animals (third and fourth columns, mice #2 and #3) were arrested in the same phase of the step cycle (across rows) and at different phases of the step cycle (across columns). The step phase for each row corresponds to the diagram in **a**. Every pair of traces in the same row shows the muscle activity of the *Tibialis anterior* (TA, upper trace) and the *Soleus* (Sol, bottom trace) during the evoked arrest. The arrest evoked by Chx10-PPN neuron activation at any given phase of the step cycle leads to a similar posture and EMG pattern in TA and Sol both in the same animal and across animals. Purple arrowheads and lines mark the moment of movement arrest for each hindlimb. Blue shades delimit light stimulus duration (2 s —mice #1 and #3— or 1 s —mouse #2— at 40 Hz).

Electromyography (EMG) recordings of hindlimb ankle flexor (tibialis anterior) and extensor (soleus) muscles revealed that Chx10-PPN activation did not evoke de novo muscle activity, neither leading to a specific activation pattern nor to a consistent flexor–extensor co-activation pattern. Instead, the flexor–extensor and left–right alternating pattern observed during walk and trot was paused and kept on hold maintaining a given combination through the stimulation. Both the coordination of activity between muscles and the joint positions during the pause remained constant once the animal adopted its arrest position (Fig. 3a and Extended Data Figs. 3 and 4). The pause could coincide with any phase of the step cycle, leading to unique arrest patterns observed within and across animals (Extended Data Figs. 3 and 4). If a limb was held immobile during stance, the ankle extensor remained tonically active during the stimulation. Instead, if a limb was held immobile during swing phase, the ankle flexor remained active. In both cases, the corresponding antagonist remained silent and only became active once the alternating gait resumed after light offset (Fig. 3a and Extended Data Figs. 3 and 4). If the limb arrested at mid-swing or late-swing phase, the tonic activity in the ankle flexor could be progressively declining through the stimulus duration due to a small tonic postural adjustment of the limb (for example, Extended Data Fig. 3c). We conclude that the Chx10-PPN-evoked arrest has a ‘context-dependent’ pattern, where the muscle activity during arrest reflects the position of the limbs at the time of movement interruption.

We then analyzed hindlimb positions during arrest in the linear corridor (Fig. 3b). In each trial, we determined whether the limb was in mid-stance or swing phase (Methods). The left and right hindlimbs were in stance phase in 62.5% (25/40) and 70% (28/40) of the trials, respectively, while both hindlimbs were in mid-stance phase in 32.5% (13/40) of the trials. When we determined the hindlimb-to-body angles of the stance-to-stance trials in relationship to the body axis, in only five trials were the two paws perpendicularly aligned on the ground, while the rest of stance-to-stance trials had either of the paws leading. Thus, during the Chx10-PPN-evoked arrest, the hindlimbs did not adopt stereotyped stance–swing or stance–stance phases but showed all possible combinations.

**Extended Data Fig. 5:**
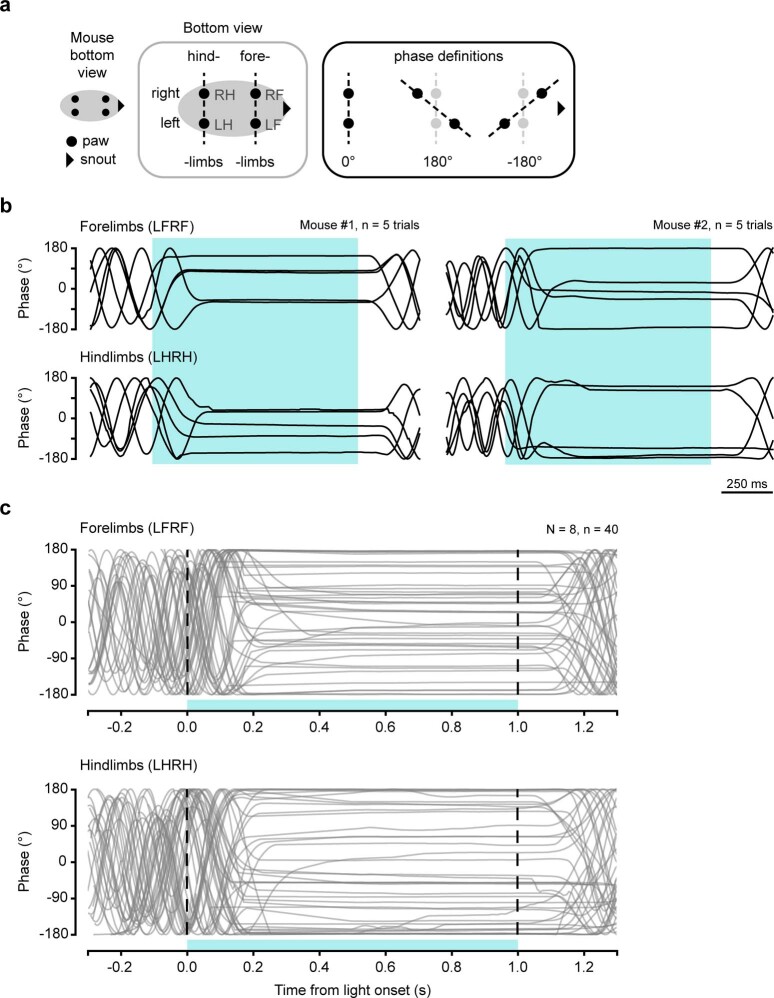
Chx10-PPN activation can pause locomotion at any position without a preferred phase and in a subject independent manner. **a**, Explanatory diagrams of the analysis used to assess left-right limb coordination based on paw tracking from the bottom view of the Chx10-PPN stimulation trials in the linear corridor. Phase values are defined by the distance on the *x* axis between left and right fore- or hind-limbs. **b**, Phase values for forelimbs (LFRF, top) and hindlimbs (LHRH, bottom) of two different mice (5 trials each) during locomotion in the linear corridor, temporarily interrupted by optogenetic Chx10-PPN neuron stimulation (blue shade). **c**, Same as **b** but for all trials of all mice (N = 8, n = 40 trials total, 5 trials/mouse). Blue shades (**b**, **c**) delimit light stimulus duration.

In all the linear corridor trials, we also tracked the paws from the bottom view to analyze left–right limb coordination. We assigned phase values to the position of each left (L) and right (R) limb pair (forelimbs (F), LFRF; hindlimbs (H), LHRH) based on the distance between the left and right paws on the *x* axis (Fig. 3c and Methods). The continuous phase values during locomotion resulted in sinusoidal traces that illustrate left–right alternation, where each peak-to-peak or trough-to-trough corresponds to one step (stance to stance) for either the left or the right limb, respectively. During Chx10-PPN stimulation, the regular alternating pattern became a flat line because paw positions did not change during arrest (Fig. 3d). The arrest position was not subject-dependent because the same mouse could exhibit different arrest patterns (Extended Data Fig. 5b). By plotting all trials together (*N* = 8 mice, *n* = 40 trials, 5 trials per mouse), we confirmed that Chx10-PPN neurons can pause locomotion at virtually any position, covering the entire range of possible values of left–right limb displacement for forelimbs and hindlimbs (Extended Data Fig. 5c). This pattern highlights that Chx10-PPN neurons can halt movement at any point of the step cycle without lateralization, although the stimulation was delivered unilaterally on the same hemisphere in all mice.

Next, we assessed how the left–right alternation continued once mice resumed locomotion. We found that if the flat line that represents the arrest was virtually cut out of the trace, after light offset the step cycle continued with the expected rhythmic pattern regardless of the left–right coordination pattern during arrest (Fig. 3e). To quantify this phenomenon, we defined a pause and a play time point for each limb pair of each trial and calculated the phase difference between them (Fig. 3f and Methods). The pause–play phase differences were concentrated around 0° (medians: LFRF = −2.66°, LHRH = 0.91°; interquartile range (IQR): LFRF = −10.20° to 12.39°, LHRH = −8.81° to 12.70°) with few larger values (minimum, maximum: LFRF = −45.64°, 81.62°; LHRH = −32.40°, 63.49°), corresponding to trials where limbs were arrested at maximum displacement in the air and mice performed small readjustments due to the unstable posture. To quantify step cycle continuity, we compared the signs of the slopes before pause and after play while considering if the arrest happened midway (rising/falling) or at the edges (peak/trough; Methods), allowing us to compute continuity as a binary outcome. In 97.5% (39/40) and 92.5% (37/40) of the trials for forelimbs and hindlimbs, respectively, mice continued the step cycle with the expected coordination pattern (Fig. 3f). Thus, after light offset, mice resumed locomotion by continuing from the same posture they paused, and finished the step cycle with the expected coordination pattern (Supplementary Video 1). We called this pattern ‘pause-and-play’ because during the Chx10-PPN-evoked pause the nervous system appeared to keep a memory of the movement, until it released and continued its intended course of action.

The pause-and-play pattern was not exclusive for straight forward locomotion, and although not analyzed kinematically, it was also observed in the other assessed motor behaviors (grooming, rearing and ambulation; Supplementary Videos 2 and 3). Indeed, ChR2-expressing mice continued the interrupted grooming bout for at least 500 ms after light offset in 61.23% of the trials. In contrast, in only 35% of the trials from EYFP-expressing control mice were the animals engaged in grooming 500 ms after light offset.

These findings show that the Chx10-PPN arrest command can interrupt any motor program at any time point of its execution without a stereotypically evoked kinematic or postural pattern associated to it. The ongoing motor sequence is thus interrupted and frozen in time –pause– until light offset, when movement resumes by continuing the motor sequence –play–.

### Apnea and a reduction in heart rate accompany the motor arrest

**Fig. 4:**
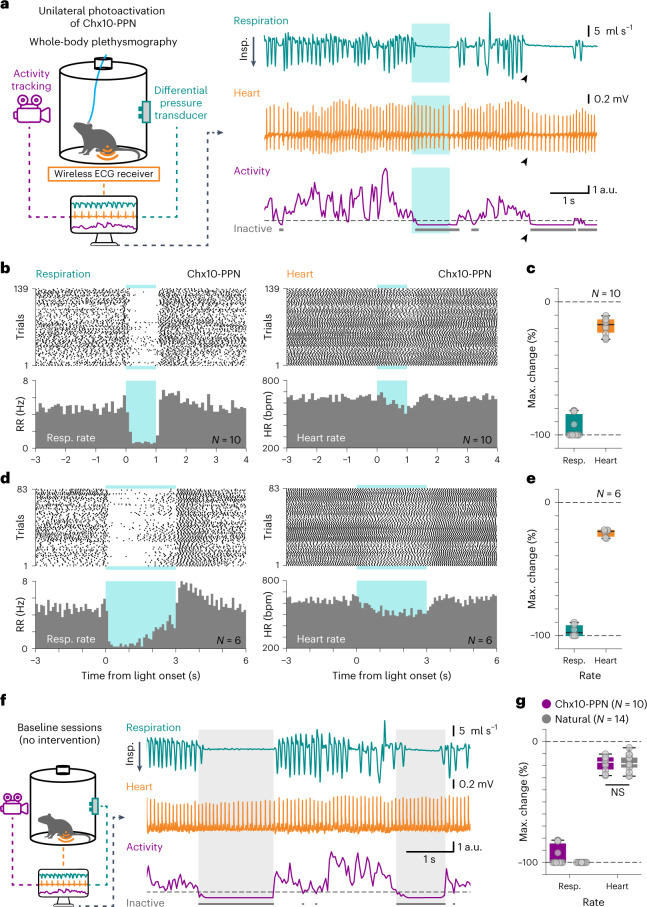
Apnea and bradycardia accompany Chx10-PPN-induced motor arrest. **a**, Left, experimental strategy for simultaneous recording of respiratory, cardiac and motor activity in unrestrained mice combined with optogenetics for activation of Chx10-PPN neurons. Right, example traces containing a snippet of all the recorded signals during and around a blue-light stimulation event: the plethysmography trace shows respiratory activity through flow changes where downward deflections indicate inspiration (top, cyan), the ECG trace shows cardiac activity (center, orange) and the activity trace reflects movement (bottom, magenta), with inactive periods tagged in gray. The horizontal dashed line marks the inactivity threshold. Photoactivation of Chx10-PPN neurons (blue shade) evokes apnea, a reduction in heart rate and motor arrest. Black arrowheads point to a naturally occurring arrest event with a similar pattern in all three signals in the absence of experimental manipulations. **b**, Raster plots (top) and PSTHs (bottom) of the respiratory rate (left) and the heart rate (right) around Chx10-PPN neuron activation with blue light for 1 s (*N* *=* 10 mice, *n* *=* 139 trials). **c**, Maximum change in average respiratory rate and average heart rate during blue-light activation of Chx10-PPN neurons compared to the baseline average rates. **d**,**e**, Same as in **b** and **c**, but for 3 s of blue-light stimulation (*N* *=* 6 mice, 83 trials). **f**, Same as in **a** but for baseline sessions in the absence of any experimental intervention. Gray shades delimit naturally occurring apneic arrest events. **g**, Same as in **c** but comparing the 1-s blue-light activation of Chx10-PPN neurons (magenta) to naturally occurring long apneic arrest events (gray; *N* *=* 14 mice), where the heart rate also changed similarly (two-tailed unpaired *t*-test, PPN versus natural, NS). In box-and-whisker plots, lines indicate medians, box edges the IQR, and whiskers extend to the minimum and maximum. Circles represent individual mice. Blue shades (**a**, **b** and **d**) delimit light stimulus duration. Source data

As the PPN plays a role in autonomic regulation^44,45^, we wondered whether Chx10-PPN activation also had effects beyond the limbed motor output, and thus investigated its effect on respiration and heart rate (see Supplementary Information for further details). We recorded unrestrained awake mice using simultaneous whole-body plethysmography (WBP), wireless electrocardiography (ECG) and activity tracking while delivering optical stimulation to Chx10-PPN neurons (blue or yellow light as a control) with random intertrial intervals (Fig. 4a).

**Extended Data Fig. 6:**
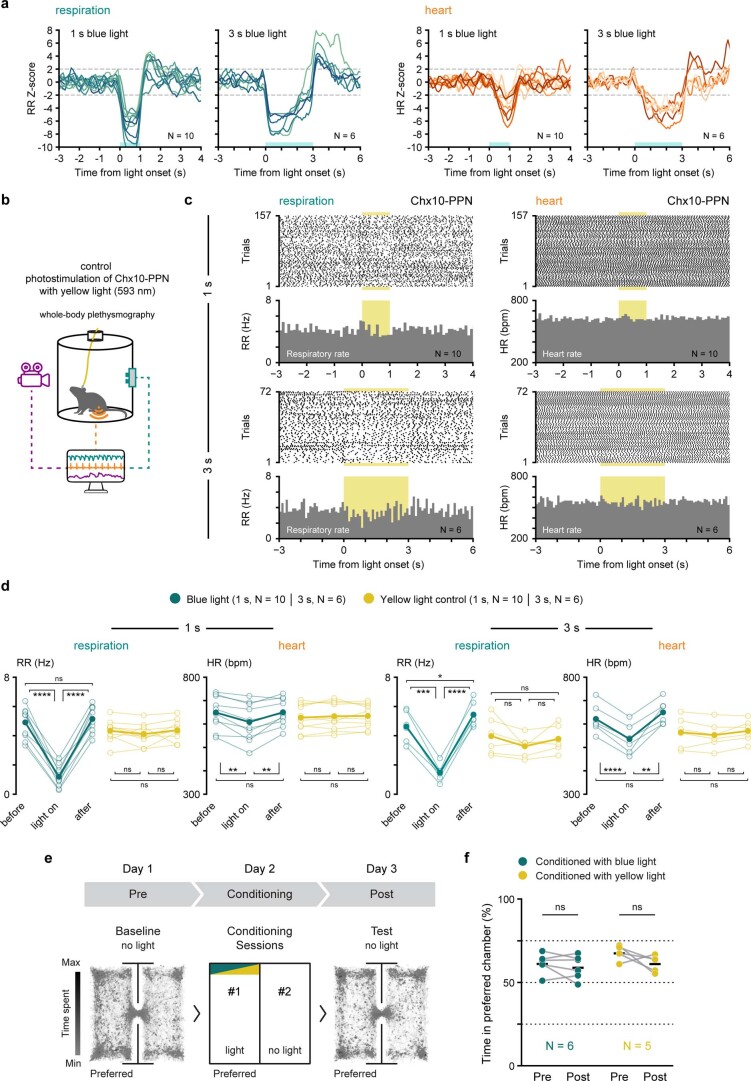
Consistency of respiratory and heart rate changes, control for the wavelength specificity of light stimulation, and conditioned place aversion test upon Chx10-PPN neuron stimulation. **a**, Z-score for respiratory rate (RR; left, blue lines) and heart rate (HR; right, orange lines) upon Chx10-PPN neuron activation with either 1 or 3 s of blue light (baseline = 5 s before light onset for each mouse). Each line represents a mouse (N = 10 mice for 1 s, N = 6 mice for 3 s). **b**, Experimental strategy for simultaneous recording of respiratory, cardiac, and motor activity in unrestrained mice combined with optogenetics for control stimulation of Chx10-PPN neurons with yellow light (593 nm). **c**, Raster and peri-stimulus time histograms of the respiratory rate (RR, left) and the heart rate (HR, right) around yellow light delivery as a wavelength specificity control in mice expressing ChR2 in Chx10-PPN neurons (top, 1 s stimulation, N = 10 mice, 157 trials; bottom, 3 s stimulation, N = 6 mice, n = 72 trials). Yellow shades delimit yellow-light stimulus duration. **d**, Average respiratory rate (RR) and heart rate (HR) during each of the time epochs surrounding blue or yellow light stimulation (5 s before, 1 or 3 s light on, 5 s after). As opposed to blue light, yellow light does not evoke any significant changes in respiratory or heart rates (statistical comparisons correspond to adjusted *P* values from Tukey’s multiple comparisons test, see Supplementary Table 1 for details). For each rate, stimulus duration, and wavelength: hollow circles are individual mice and filled circles the group average, all connected across epochs. The same mice were first tested with blue light and then with yellow light (N = 10 for 1 s stimulus duration; N = 6 for 3 s). **e**, Timeline of the conditioned place aversion (CPA) paradigm with baseline (‘Pre’) and test (‘Post’) heatmaps representing the time spent across the entire arena for all mice conditioned with blue light (10 ms pulse width, 1 s train duration, 40 Hz; 1 s ON / 9 s OFF in a 30 minute session). **f**, Percentage of time that mice spent in their preferred chamber before (‘Pre’) and after (‘Post’) conditioning with blue light (left, N = 6 mice) or yellow light (right, N = 5 mice). Neither blue nor yellow light caused a change in place preference. Two tailed paired *t*-test, ‘Post’ vs. ‘Pre’: blue light, mean difference = −2.22 ± 5.87 % (s.d.), CI = −8.38 to 3.93, not significant (ns); yellow light, mean difference = −6.44 ± 8.4 % (s.d.), CI = −16.86 to 3.98, ns.

Unilateral activation of ChR2-expressing Chx10-PPN neurons (blue light, 40 Hz, 1 s) visibly affected respiration for the duration of the stimulus (Fig. 4a,b; *N* = 10 mice, *n* = 139 trials). The mean respiratory rate dropped from a baseline average of 4.79 Hz (5 s before light onset) to an average of 1.14 Hz during the entire stimulation period, and to 0.66 Hz during the last 800 ms of the stimulation, once mice were completely immobile (Fig. 4b; peri-stimulus time histogram (PSTH)). In most trials (108/139), the respiratory block was complete, leading to apnea throughout the stimulation period, while in the remaining trials, the respiratory frequency was severely reduced. This effect was consistent in all mice (Extended Data Fig. 6d; 1 s, blue light; Tukey’s multiple-comparisons test, adjusted *P* values: before versus light on and light on versus after, *P* < 0.0001; before versus after NS). After light off, the respiratory arrest/slowing was followed by a rebound that lasted for approximately 1 s (6.16 Hz between 0.1 and 1.1 s after light off) before returning to baseline levels (4.85 Hz between 1.1 and 5 s after light off; Fig. 4b, PSTH; Extended Data Fig. 6a,d), but was not significant (see above, before versus after). Moreover, the activation of ChR2-expressing Chx10-PPN neurons caused a mild reduction in heart rate that was most prominent toward the end of the stimulation (Fig. 4b; average of 637 beats per minute (bpm.) during the first 500 ms of stimulation dropping to 572 bpm. during the last 500 ms; baseline average rate, 646 bpm.). The heart rate change was significant between epoch averages (Extended Data Fig. 6d; 1 s, blue light; Tukey’s multiple-comparisons test, adjusted *P* values: before versus light on, *P* = 0.0025; light on versus after, *P* = 0.0071; before versus after NS). The average maximum change from baseline in respiratory rate was −93.81% ± 8.51% (mean ± s.d.; minimum, maximum: −81.62%, −100%; Fig. 4c), while the heart rate was −18.48% ± 6.19% (mean ± s.d.; minimum, maximum: −10.45%, −28.24%; Fig. 4c), corresponding in all mice to a significant drop in both rates compared to baseline.

**Extended Data Fig. 7:**
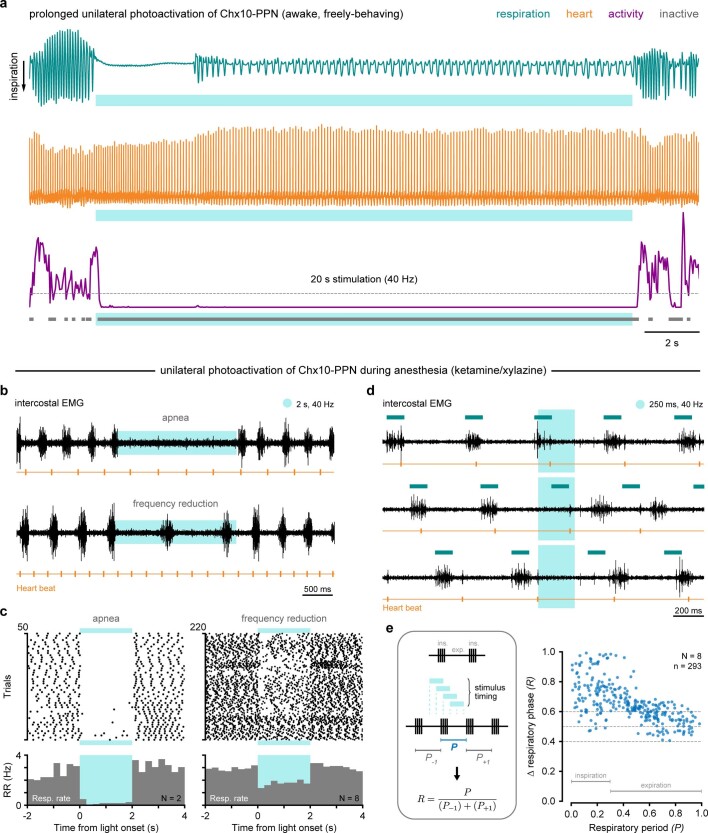
The duration of motor arrest is time-locked to Chx10-PPN activation and the evoked respiratory changes are a direct effect of Chx10-PPN neuron activation decoupled from motor arrest. **a**, Example traces of respiratory (top, cyan), cardiac (middle, orange) and motor activity (bottom, magenta) from unrestrained mice recorded in the plethysmograph during a 20-second-long stimulation train with blue-light activating Chx10-PPN neurons. The horizontal dashed line marks the inactivity threshold; inactive periods are tagged in gray. Activation of Chx10-PPN neurons (blue shade) leads to motor arrest for the entire duration of the stimulus. Mice first exhibit apnea for up to ~3.5 seconds but resume slow breathing afterwards ensuring survival. **b**, Example intercostal EMG recordings upon activation of ChR2-expressing Chx10-PPN neurons during anesthesia. Top, example trial where activation leads to apnea. Bottom, example trial where activation leads to a reduction of the respiratory frequency. In both cases heart beats are shown in orange below the EMG recordings. **c**, Raster plots and peri-stimulus time histograms of trials where stimulation leads to apnea (left; N = 2 mice, n = 50 trials) or to frequency reduction (right; N = 8 mice, n = 220 trials). RR, respiratory rate. **d**, Three examples of intercostal EMGs during anesthesia upon delivery of short 250 ms trains of blue light (40 Hz, 10 ms pulse width) to activate ChR2-expressing Chx10-PPN neurons. Cyan rectangles mark the expected position of the respiratory bursts based on the burst timing prior to light stimulation. The mismatch between actual and expected burst timing in the middle and bottom traces shows that activation of Chx10-PPN neurons has a direct effect on respiration and is able to disturb the respiratory period. **e**, Population level quantification of the effect over the respiratory rhythm shown in **d** depicted as a phase response curve (N = 8 mice, n = 293 trials). The *x* axis corresponds to the time within the respiratory period (*P*) when the stimulation train starts. Within *P*, the inspiratory phase extends (EMG burst) approximately from 0 to 0.3. The *y* axis corresponds to the value (*R*) of the respiratory phase shift (if any) happening during *P*, calculated as shown in the explanatory diagram (left). *R* values between 0.4 and 0.6 indicate no change in *P* as exemplified in **d** (top). *R* values > 0.6 indicate a prolonged *P* with two possible scenarios depending on stimulus timing: if the stimulation overlaps with the burst onset, it leads to the silencing of the inspiratory burst and subsequent phase advance and resetting of the inspiratory rhythm as exemplified in **d** (middle); if the stimulations strictly overlaps with the expiratory phase, it delays the next burst without resetting of the rhythm as exemplified in **d** (bottom). In all panels blue shades delimit stimulus duration.

The respiratory and heart rate reduction was also consistently observed with longer stimulus lengths (3 s; Fig. 4d,e, Extended Data Fig. 6a,d and Supplementary Information). In contrast, stimulation with yellow light (593 nm, 40 Hz, 1 or 3 s) as a control for ChR2 activation had no effect on respiratory or heart rate (Extended Data Fig. 6b–d). Interestingly, despite the strong motor and respiratory modulation, mice did not perceive Chx10-PPN neuron activation as aversive (Extended Data Fig. 6e,f). For prolonged stimulation periods (20 s), the respiratory centers were able to escape the arrest and set to a slower rhythm ensuring survival (Extended Data Fig. 7a and full description in Supplementary Information).

A well-known link exists between locomotor activity and changes in respiratory rhythm^46,47^. A possible explanation for the observed respiratory changes could therefore be that they are secondary to the motor arrest evoked from Chx10-PPN activation. However, changes in respiratory and heart rate were also observed in anesthetized mice and are thus independent from movement arrest (Extended Data Fig. 7b–d and Supplementary Information). Moreover, the effect over respiration seems to be directly mediated through inhibition of the inspiratory rhythm generation (Extended Data Fig. 7d and Supplementary Information).

Collectively, these results add additional features to the phenotypic fingerprint of the Chx10-PPN-evoked motor arrest: during the pause period, animals show apnea and mild bradycardia, and during the play period, respiration overshoots but quickly returns to pre-stimulus values as does the heart rate. Importantly, we found that mice spontaneously perform arrest bouts with similar motor and autonomic features during baseline conditions in the absence of experimental neuronal manipulations, although their expression was highly heterogeneous between animals (Fig. 4a,f,g; full description of results in Supplementary Information). These results confirm that the behavioral pattern observed upon Chx10-PPN neuron activation also exists under natural conditions, possibly elicited by natural triggers such as salient environmental cues.

### vlPAG-evoked freezing is different from Chx10-PPN arrest

**Fig. 5:**
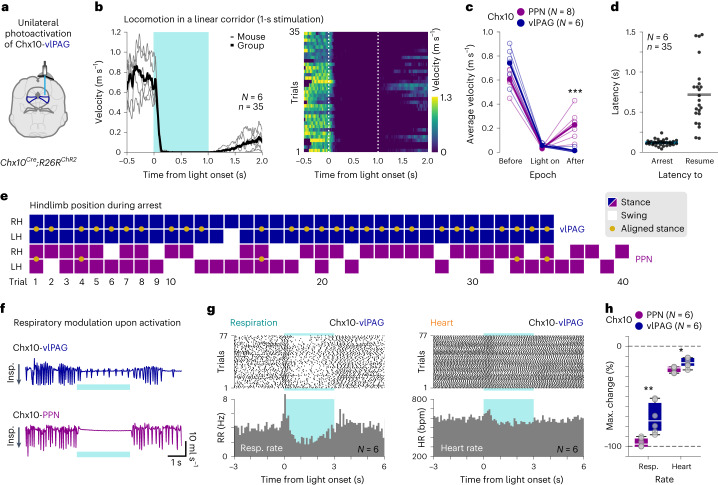
The Chx10-PPN evoked global motor arrest differs from Chx10-vlPAG induced fear-related freezing. **a**, Experimental strategy to optogenetically target Chx10^+^ neurons in the vlPAG using *Chx10*^*Cre*^; *R26R*^*ChR2*^ mice. **b**, Effect on velocity of Chx10-vlPAG neuron photoactivation during locomotion in a linear corridor. Left, group average velocity (black line; 35 trials) and mouse average velocities (gray lines; *N* *=* 6 mice, 3–8 trials/mouse). Right, velocity heat map for individual trials. Dashed vertical lines delimit light onset and offset. **c**, Average locomotor velocities while crossing the linear corridor before light onset (before), during stimulation (light on), and after light offset (after), when activating Chx10^+^ neurons in the PPN (magenta, *N* = 8 mice) or the vlPAG (blue, *N* *=* 6 mice; two tailed Mann–Whitney test, PPN versus vlPAG, after, *P* *=* 0.0007). Hollow circles are individual mice (mouse average), and filled circles are the group average. **d**, Latencies to arrest locomotion from light onset (left), and latencies to resume locomotion from light offset (right). Dots are individual trials and lines are group means. **e**, Hindlimb (RH, right; LH, left) position during arrest in the linear corridor for Chx10-vlPAG (blue) and Chx10-PPN (magenta) neuron stimulation trials. Solid squares represent stance, while empty squares represent swing. Yellow dots denote that RH and LH are aligned perpendicular to the body axis. **f**, Example plethysmography traces upon Chx10-vlPAG (top, blue) and Chx10-PPN (bottom, magenta) neuron activation. Downward deflections correspond to inspiration. Data were acquired using the same experimental setup as in Fig. 4. **g**, Raster plots (top) and PSTHs (bottom) of the respiratory rate (left) and the heart rate (right) around Chx10-vlPAG activation (*N* *=* 6 mice, 77 trials). **h**, Maximum change in average respiratory and heart rates during activation of Chx10-PPN (magenta) or Chx10-vlPAG (blue) neurons compared to their baseline average rates (two-tailed Mann–Whitney test, PPN versus vlPAG, respiratory rate: *P* *=* 0.0022; heart rate: *P* *=* 0.0260). In box-and-whisker plots, lines indicate medians, box edges denote the IQR, and whiskers extend to the minimum and maximum values. Circles represent individual mice (*N* *=* 6 mice in both groups). Blue shades (**b**, **f** and **g**) delimit light duration. Source data

**Extended Data Fig. 8:**
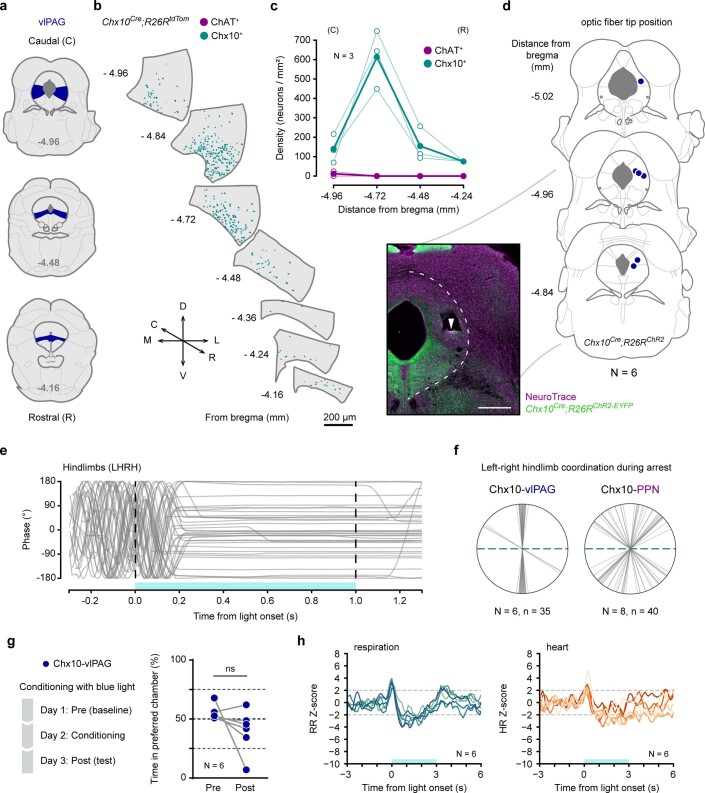
Distribution of Chx10^+^ neurons within the vlPAG, optic fiber positions, limb coordination, conditioned place aversion test, and heart and respiratory changes upon Chx10-vlPAG neuron activation. **a**, Coronal plane schematics highlighting the vlPAG (blue) at different levels of its rostro-caudal axis. **b**, Spatial distribution of Chx10^+^ (cyan, *Chx10*^*Cre*^; *R26R*^*tdTomato*^ reporter mouse) and ChAT^+^ (magenta, antibody) neurons along the rostro-caudal axis of the vlPAG. Only the coronal levels shared with the PPN are shown to facilitate comparison (see Fig. 1) although the vlPAG extends further caudally. C, caudal; R, rostral; D, dorsal; V, ventral; M, medial; L, lateral. **c**, Quantification of Chx10^+^ (cyan) and ChAT^+^ (magenta) neuron densities at four coronal levels of the vlPAG: −4.96 and −4.72 mm from the caudal half, −4.48 and −4.24 mm from the rostral half. For each cell type: hollow circles are individual mice and filled circles the group average (N = 3 mice, 1 hemi-section/level/mouse). **d**, Reconstruction of the optic fiber tip position in all *Chx10*^*Cre*^; *R26R*^*ChR2*^ mice used for Chx10-vlPAG neuron stimulation experiments (N = 6) and example fluorescence photomicrograph of optic fiber placement. The white arrowhead points to the tip of the optic fiber. Scale bar 500 µm. **e**, Phase values for left-right hindlimb (LHRH) coordination during locomotion in the linear corridor temporarily interrupted by optogenetic Chx10-vlPAG neuron stimulation (N = 6, n = 35 trials total, 3–8 trials/mouse). **f**, Left-right hindlimb coordination during arrest for Chx10-vlPAG (left) and Chx10-PPN (right) represented by the line that unites the left and right hind-paws corrected for body axis. Each gray line corresponds to a trial in the linear corridor. Dashed blue lines correspond to the body axis. **g**, Conditioned place aversion (CPA) timeline (left) and quantification (right) of the percentage of time that mice spent in their preferred chamber before (‘Pre’) and after (‘Post’) conditioning with blue light (10 ms pulse width, 1 s train duration, 40 Hz; 1 s ON / 9 s OFF in a 30-minute session) in (N = 6 mice). Two tailed Wilcoxon matched-pairs signed rank test, ‘Post’ vs. ‘Pre’: not significant (ns). **h**, Z-score for respiratory rate (RR; left, blue lines) and heart rate (HR; right, orange lines) changes evoked upon Chx10-vlPAG neuron activation with 3 s of blue light. Each line represents a mouse (N = 6 mice for 3 s). Blue shades delimit light stimulus duration.

Activation of glutamatergic neurons in the ventrolateral column of the periaqueductal gray (vlPAG) elicits global motor arrest associated with innate fear responses^12^. Moreover, a Chx10-expressing subpopulation of the glutamatergic neurons in the vlPAG also elicits a freezing response^48–50^. Because the vlPAG is close to the PPN and both contain Chx10^+^ neurons (Extended Data Figs. 1 and 8a–c), we asked if stimulation of Chx10-vlPAG neurons would evoke a similar motor arrest as Chx10-PPN stimulation does. To target Chx10-vlPAG neurons, we crossed *Chx10*^*Cre*^ with *R26R-ChR2-EYFP* mice, which led to constitutive ChR2 expression in Chx10^+^ neurons as in Vaaga et al.^49^. The transgenic approach allowed us to evoke freezing by a unilaterally implanted optical fiber above the vlPAG (Fig. 5a and Extended Data Fig. 8d) instead of the bilateral stimulation required with viral infections^12,50^.

We optogenetically activated Chx10-vlPAG cells using the same parameters as in Fig. 2 (blue light; 40 Hz, 1 s, 10-ms pulse width) while mice were locomoting in a linear corridor. Activation of Chx10-vlPAG neurons reliably arrested ongoing locomotion in all trials (*N* = 6 mice, *n* = 35, 3–8 trials/mouse; Fig. 5b and Supplementary Video 4). The average velocity decreased from 0.74 ± 0.14 m s^−1^ before light to 0.06 ± 0.02 m s^−1^ during the light on epoch (mean ± s.d.; Fig. 5c), similar to the speed drop observed upon Chx10-PPN activation (Figs. 2c and 5c; Chx10-PPN versus Chx10-vlPAG: 0.61 ± 0.07 m s^−1^ versus 0.74 ± 0.14 m s^−1^ before, and 0.03 ± 0.01 m s^−1^ versus 0.06 ± 0.02 m s^−1^ during light on, mean ± s.d.). However, the average velocity after light was higher in Chx10-PPN mice, as Chx10-vlPAG mice remained largely immobile after stimulus offset (Chx10-PPN versus Chx10-vlPAG: 0.23 ± 0.11 m s^−1^ versus 0.01 ± 0.02 m s^−1^ after light off, mean ± s.d.; mean difference after light off = 0.22 m s^−1^, CI = 0.1 to 0.33 m s^−1^; two-tailed Mann–Whitney test, PPN versus vlPAG, after, *P* = 0.0007; Fig. 5c). The latency to arrest from light onset was in the same range as in the Chx10-PPN-induced global motor arrest (PPN versus vlPAG: 110.83 ± 44.01 ms versus 118.1 ± 35.85 ms, mean ± s.d.; Fig. 5d). However, there was a much slower and variable recovery time after light offset when Chx10-vlPAG neurons were activated compared to Chx10-PPN neurons (latency to resume motion, PPN versus vlPAG: 148.33 ± 73.66 ms versus 717.39 ± 383.81 ms, mean ± s.d.; mean difference = −569.1 ms, CI = −665.7 to −472.4 ms) and, as a consequence, a longer total time spent immobile (PPN versus vlPAG: 1.04 ± 0.1 s versus 1.6 ± 0.4 s, mean ± s.d.; Fig. 5d; see Fig. 2d for comparison). In 12/35 of Chx10-vlPAG stimulation trials, mice did not resume locomotion during the post-stimulus observation period (1.5 s). These trials were excluded from the calculations of latency to resume locomotion and total time immobile, resulting in an underestimation of both parameters as reported here. Interestingly, the limb position during Chx10-vlPAG-evoked freezing had a stereotyped pattern (Fig. 5e and Supplementary Video 4). In 97.14% (34/35) of the Chx10-vlPAG stimulation trials, both hindlimbs were in stance phase during the stimulation, and in most cases (27/35), these were aligned perpendicular to the body axis during the arrest (Fig. 5e). This stereotyped arrest position is in sharp contrast to the variable arrest positions observed upon Chx10-PPN neuron stimulation (Fig. 5e), which was also apparent when analyzing limb coordination (Extended Data Fig. 8e,f and Supplementary Information). Moreover, visual evaluation of the behavior shows that mice either restart a new step cycle from the resting position adopted during arrest or engage in a behavior different from locomotion, for example, exploratory sniffing, with no resemblance to the pause-and-play pattern (Supplementary Video 4). Thus, although both Chx10-PPN and Chx10-vlPAG neuron activation leads to global motor arrest, the dynamical expression of the evoked behaviors is very different. Yet another difference between Chx10-PPN and Chx10-vlPAG stimulation is that when tested for conditioned place aversion, 5/6 mice changed their preference after opto-pairing (Chx10-vlPAG neuron activation), although in most cases this shift was mild and not significant at a group level (Extended Data Fig. 8g).

Since defensive responses mediated by the PAG are accompanied by cardiovascular and respiratory changes^50–52^, we also investigated respiratory and heart rate modulation upon Chx10-vlPAG activation. Generally, Chx10-vlPAG activation reduced respiratory frequency compared to the Chx10-PPN-induced apnea (Fig. 5f). Chx10-vlPAG neuron stimulation caused an initial short period of tachypnea followed by a reduction of the respiratory frequency but without apnea (Fig. 5g; for comparison with PPN, see Fig. 4d). From a baseline average of 4.13 Hz, the respiratory rate increased to 6.58 Hz during the first 300 ms of stimulation and dropped to 2.71 Hz for the rest of the stimulus duration, returning to 4.29 Hz after light offset (Fig. 5g, PSTH). Heart rate modulation was also different. After an initial short-lasting phase of tachycardia, Chx10-vlPAG stimulation was followed by a mild reduction in heart rate (Fig. 5g; for comparison with PPN, see Fig. 4d; all trials: 601 bpm baseline average, 668 bpm during the first 400 ms of stimulation, 558 bpm for the rest of stimulus duration, and back to 600 bpm after light off; Fig. 5g, PSTH). The temporal dynamics in respiratory and heart rate were consistent across animals and different from Chx10-PPN neuron activation (Extended Data Fig. 8h; for comparison with PPN, see Extended Data Fig. 6a).

The average maximum change from baseline in respiratory rate after Chx10-vlPAG activation was −71.34% ± 15.90% (mean ± s.d.; minimum, maximum: −52.06%, −88.37%), in contrast to a larger drop of −96.25% ± 4.48% (mean ± s.d.; minimum, maximum: −90.18%, −100%) after Chx10-PPN activation (difference between means = −24.91%, CI = −41.55% to −8.26%, *N* = 6 in each group; Mann–Whitney test, PPN versus vlPAG, *P* = 0.0022; Fig. 5h). The maximum reduction in heart rate during Chx10-vlPAG stimulation was smaller than during Chx10-PPN activation but within a partly overlapping range (mean ± s.d. PPN versus vlPAG: −23.05% ± 15.90% versus −16.52% ± 4.85%; minimum, maximum PPN versus vlPAG: −20.64%, −27.01% versus −11.50%, −23.68%; difference between means = −6.54%, CI = −11.83% to −1.25%; Mann–Whitney test, PPN versus vlPAG, *P* = 0.0260; Fig. 5h). Our findings demonstrate that although the evoked responses overall change in the same direction, respiration and heart rate are differentially affected upon Chx10-vlPAG and Chx10-PPN activation, suggesting that the Chx10-PPN-evoked global motor arrest is unrelated to the Chx10-vlPAG-induced global motor arrest (defensive freezing).

### Chx10-PPN neurons project widely through the neuroaxis

**Fig. 6:**
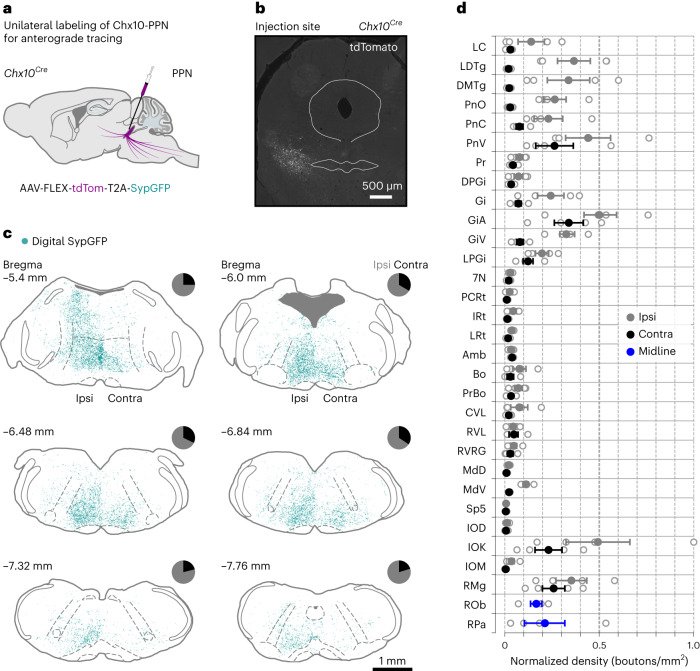
Brainstem projection targets from Chx10-PPN neurons. **a**, Experimental strategy to unilaterally label Chx10-PPN neurons with an anterograde tracer (AAV-FLEX-tdTom-T2A-SypGFP) to reveal their projection pattern. **b**, Example fluorescence photomicrograph (coronal plane) of the rostral PPN at the injection site with tdTomato-expressing Chx10-PPN neurons in white. **c**, Reconstruction of putative synaptic bouton positions (digital SypGFP, cyan) from Chx10-PPN neuron projections at six different brainstem levels. The boutons are mostly located in the medial zone of the pontine and medullary reticular formations. Pie charts at each coronal level depict the percentage of boutons ipsilateral (ipsi, gray) or contralateral (contra, black) to the injection site, with the majority of them being ipsilateral at all brainstem levels. **d**, Normalized average bouton density in selected structures located within the coronal levels reconstructed in **c**. For each structure, the ipsilateral (gray) and contralateral (black) sides are quantified separately, unless they are midline structures (blue). Error bars represent the s.e.m. and hollow circles denote individual mice (*N* = 4 mice). For an extended overview and full names of all abbreviations, see Extended Data Fig. 10, which also includes projection areas in the midbrain and diencephalon. Source data

To gain insights into how Chx10-PPN neurons effectuate their output responses, *Chx10*^*Cre*^ mice were unilaterally injected in the PPN with a Cre-dependent anterograde viral tracer (AAV1-phSyn1(S)-FLEX-tdTomato-T2A-SypEGFP-WPRE) to label their synaptic terminals (Fig. 6a,b).

**Extended Data Fig. 9:**
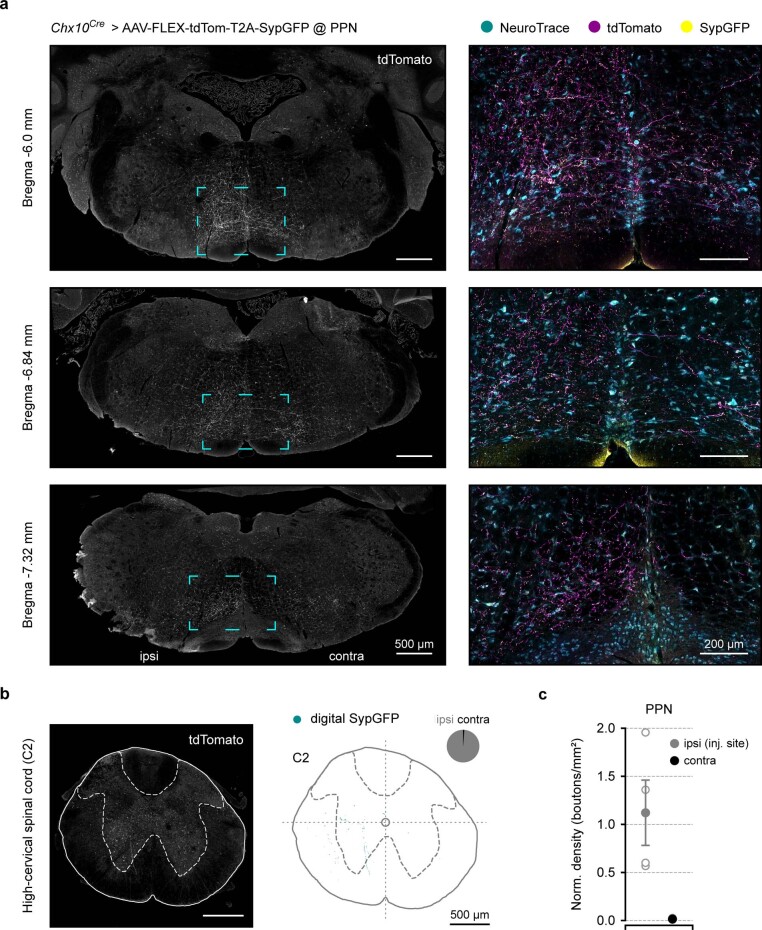
Representative images of the anterograde tracing and lack of projections to the contralateral PPN. **a**, Example confocal photomicrographs at three different brainstem levels (coronal plane) from a *Chx10*^*Cre*^ mouse unilaterally injected in the PPN with the anterograde tracer AAV-FLEX-tdTom-T2A-SypGFP to reveal the projection pattern of Chx10-PPN neurons. Left, single-channel images (grayscale) of brainstem coronal sections (excluding cerebellum) at −6.0, −6.84, and −7.32 mm from bregma. White fluorescence signal corresponds to tdTomato, which fills the cell-bodies and fibers of the transfected Chx10-PPN neurons. The whole tissue and associated anatomical landmarks are visible through background auto-fluorescence. Dashed turquoise squares delineate the magnified areas to the right. Scale bars 500 µm. Right, multi-channel composite images of the magnified areas on the left containing a fluorescent Nissl stain (NeuroTrace, cyan), fibers from the transfected Chx10-PPN neurons (tdTomato, magenta), and their synaptic boutons (SypGFP, yellow). Scale bars 200 µm. **b**, Left, example single-channel (grayscale) confocal photomicrograph of a coronal spinal cord section at C2 level (high cervical spinal cord) where white fluorescence signal corresponds to tdTomato as in **a**, showing very sparse labelling. Right, digital reconstruction of putative synaptic bouton positions (digital SypGFP, cyan) from the example cervical spinal cord image on the left. The pie chart depicts the percentage of boutons ipsilateral (ipsi, gray) or contralateral (contra, black) to the injection site. Scale bars 500 µm. **c**, Normalized bouton density (boutons/mm^2^) at the injection site (PPN, ipsilateral, gray), compared to the contralateral PPN (black). Chx10-PPN neurons do not project to the contralateral PPN. Solid circles (ipsi, gray; contra, black) are the group average (all mice), error bars represent s.e.m., and hollow circles individual mice (N = 4 mice).

**Extended Data Fig. 10:**
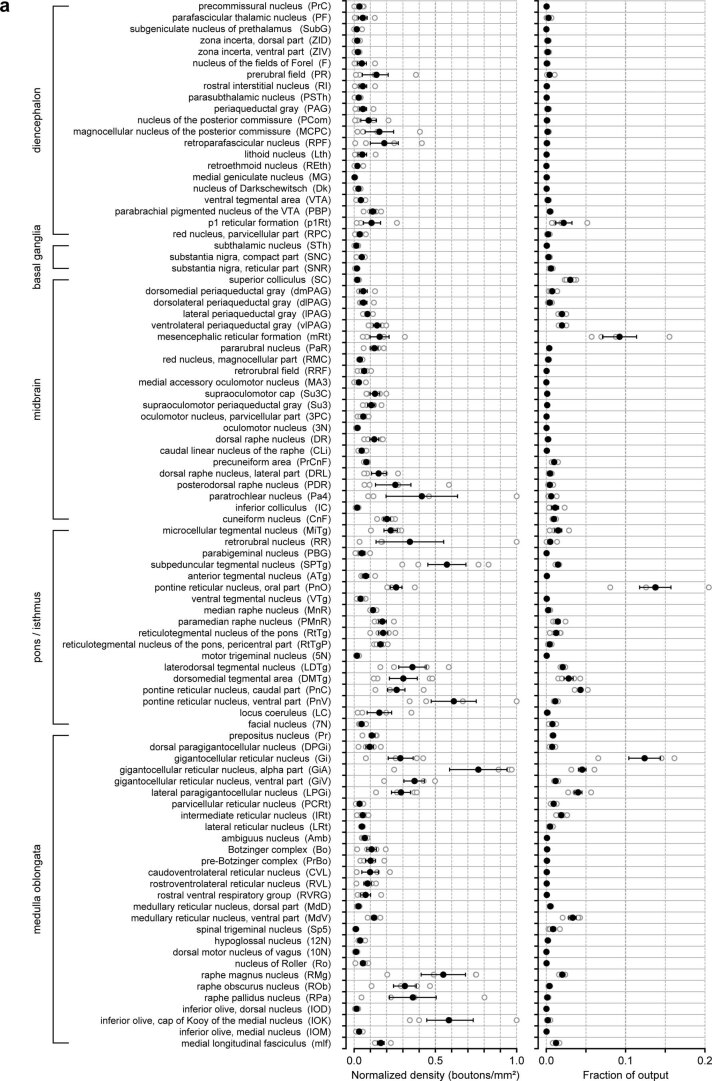
Extended overview of the anterograde tracing from Chx10-PPN neurons. **a**, Quantification from an extended selection of structures along the nervous system that receive projections from Chx10-PPN neurons. Left, normalized bouton density (boutons/mm^2^) at each anatomical structure where the min-max scaling is based on the densest structure for each mouse excluding the injection site. Right, fraction of the total output that innervates each anatomical structure, calculated as the fraction of boutons quantified within each structure from the total number of boutons segmented in that mouse. Each anatomical structure is represented by a single value per mouse comprising both the ipsilateral and contralateral sides. Nevertheless, most of the boutons for all structures are found ipsilateral to the site of injection (see Fig. 6 and Extended Data Fig. 9). The inclusion of structures in the table is based on the presence of innervation and/or general interest. Not all brain structures were analyzed and, for the sake of space, not all analyzed structures are included in the table. The overall order of appearance in the table is from rostral (top part of the table) to caudal. However, the classification into the main brain areas and the order within them was set based on the first appearance of each structure on the reference mouse brain atlas (Franklin & Paxinos^33^) starting from caudal to rostral. Therefore, some structures may belong to two different areas but are here classified as belonging to the caudal most of the two. Black solid circles are the group average (all mice), error bars represent s.e.m., and hollow circles individual mice (N = 4 mice).

Terminals from Chx10-PPN neurons were found along the neuroaxis from the diencephalon to the medulla (Fig. 6c,d and Extended Data Figs. 9 and 10), with the highest overall density located within the caudal midbrain, pons and rostral medulla, targeting all major motor-related nuclei that influence spinal motor circuits^53,54^, respiratory motor centers^55^, blood pressure-modulating centers^56,57^ and raphe nuclei with modulatory inputs to spinal motor circuits^58,59^ (Supplementary Information). In contrast, only very sparse labeling was found in the rostral-most segments of the cervical spinal cord ipsilateral to the injection site (Extended Data Fig. 9b). The projections in the diencephalon, midbrain and rostral pons were almost exclusively ipsilateral. In line with this ipsilateral bias, Chx10-PPN neurons do not project to their contralateral equivalents (Extended Data Fig. 9c). However, the descending fibers diverge bilaterally by the caudal pontine reticular nucleus (PnC). This bilateral innervation is most prominent in ventral structures at the medial column of the pontine and medullary reticular formation and extends until the caudal-most part of the medulla where it becomes mainly ipsilateral again (Fig. 6c). Despite the increase in bouton density in contralateral structures where the projections have a bilateral pattern, the highest density of boutons is still located on the ipsilateral side (Fig. 6c). Although the bulk of the projections are descending, Chx10-PPN neurons, similar to other neuron types within the PPN^20,25^, also have ascending projections to areas implicated in arousal, attention and vigilance, among others^60–62^ (Supplementary Information and Extended Data Fig. 10).

These results suggest that Chx10-PPN neurons gain their effect on motor, respiratory and autonomic functions by widely broadcasting their descending projections throughout the brainstem but without directly projecting to the spinal cord and, to a lesser extent, via ascending projections that might affect brain areas involved in controlling arousal and/or attention.

## Discussion

The present study has uncovered a brainstem command evoked upon activation of Chx10^+^ neurons in the PPN that mediates a unique type of global motor arrest. The global motor arrest is accompanied by apnea and a reduction in heart rate. The distinctive feature of the Chx10-PPN-evoked global motor arrest is a fast onset and offset and a pause-and-play pattern. This characteristic pattern differentiates the Chx10-PPN-induced motor arrest from previously described motor arrest types.

Activation of glutamatergic ‘stop neurons’ in the brainstem of fish and mice halts locomotion^7,8^. In mice this leads to a canonical stop and the adoption of a typical posture where the hindlimbs and forelimbs are on the ground simultaneously^7^. After termination of the stop, the limbs regain their walking phases from that position. Movement can also be arrested through basal ganglia activity, for example, by activation of the subthalamic nucleus^63,64^. This type of motor arrest has a slow onset and a stereotypical motor outcome. Similarly, the amygdala-driven and exploration-related stop also displays slow arrest of behavior with long onset and offset latencies^10^.

Lastly, a well-known form of motor arrest is the vlPAG-driven defensive freezing, which can be expressed as an innate behavior following threat detection or as a fear conditioned response^11,51,65^. In mice, the defensive freezing may be elicited by bilateral optogenetic stimulation of Vglut2^+^ neurons in the vlPAG^12^, or by unilateral or bilateral optogenetic stimulation of the Chx10^+^ subpopulation in the vlPAG^49,50^. For direct comparison, we also characterized the Chx10-vlPAG-evoked motor response. The dynamics of the phenotype observed during the Chx10-vlPAG-evoked freezing were quantitatively and qualitatively different to the Chx10-PPN-evoked global motor arrest: it had a slow recovery, mice typically adopted a stereotyped freezing posture with both hindlimbs on the ground and a steady slow breathing rhythm after a short initial period of tachypnea, and the bradycardic effect was smaller than upon Chx10-PPN activation. The bradycardic effect we find here upon Chx10-vlPAG stimulation has also been described by Signoret-Genest and colleagues in a recently published study^50^.

In sum, the global motor arrest evoked by Chx10-PPN activation is different from other previously described forms of motor arrest, including vlPAG-mediated freezing, as it is expressed with a distinct pause-and-play pattern (see the full discussion in Supplementary Information).

The neuronal mechanisms for the implementation of the characteristic triad of Chx10-PPN-induced output actions, that is, the global motor arrest, apnea or severe reduction in respiratory rate, and heart modulation, were not directly addressed in this study. However, based on the observed descending projection pattern, we propose that the triad involves parallel actions at the level of the brainstem followed by multiple actions at the executive motor circuits in the spinal cord, as discussed in detail within the extended discussion (Supplementary Information).

An intriguing aspect of the arrest behavior described in this work revolves around its possible function when expressed under natural conditions. Although we do not provide a causal link, we found that the same combination of motor arrest and respiratory and heart rate changes as seen upon Chx10-PPN neuron activation can be observed in baseline conditions in the absence of experimental manipulations or threatening stimuli. Moreover, we find that the amount of short arrest bouts in the open field is reduced in most mice after the ablation of Chx10-PPN neurons. A confounding factor in the ablation experiment is that movement arrest in the open field might be triggered by neuronal circuits other than Chx10-PPN neurons^7,10^. So even if we had managed to obtain a complete ablation of Chx10-PPN neurons—which will be difficult to achieve due to the elongated shape of the nucleus—it is not expected that the arrest events would fully disappear. However, the fact that there is a reduction, as opposed to the increase observed in control mice, indicates that naturally occurring motor arrest events are partly mediated by Chx10-PPN neurons.

The naturally occurring arrest events that are linked to the Chx10-PPN neurons may happen, for example, during exploration, and we hypothesize that these natural brief arrest bouts may be triggered by salient but nonthreatening sensory inputs. The temporary behavioral interruption might be accompanied by or lead to an increase in attention, possibly mediated through the ascending projections from Chx10-PPN neurons. Chx10-PPN neurons target areas that have been ascribed a role in regulating the processing of unexpected and behaviorally relevant sensory stimuli, attention, and arousal, such as the thalamic parafascicular nucleus, the laterodorsal tegmental nucleus, the locus coeruleus and the dorsal raphe nucleus^60,62,66,67^ (Extended Data Fig. 10).

The attentional shift when reacting to novel environmental cues might be facilitated by the global motor arrest, but it could also be either the trigger or a consequence of it. Regardless of chronology, we hypothesize that the arrest evoked from the activation of Chx10-PPN neurons could be embedded within an attention-related cognitive state. Such a role would highlight the integrative role of the PPN as a whole in driving both motor and cognitive aspects for a coherent behavioral response (see the extended discussion in Supplementary Information).

Our study demonstrates that a subpopulation of glutamatergic neurons in the PPN has a movement-opposing effect. Therefore, the results presented in this work together with previous evidence lead to a model where the PPN has a dual opposing role in motor control depending on the subpopulation of glutamatergic cells involved: activation of glutamatergic neurons predominantly located in the caudal part of the nucleus promotes locomotion^26–30^, while the specific activation of glutamatergic Chx10-PPN neurons, which are enriched in the rostral PPN, evokes global motor arrest. This functional diversity within glutamatergic PPN neurons could explain why some studies have been unable to demonstrate locomotor initiation by broad glutamatergic PPN neuron activation^31,32^ (see extended discussion in Supplementary Information).

Definitive evidence for this proposal would require the stimulation of Chx10-negative/Vglut2-positive PPN neurons locally (caudally or rostrally) or broadly by using an intersectional approach, which we have not done here. However, our study shows that the arrest is solely linked to the Chx10-PPN neurons, which are glutamatergic and enriched in the rostral part and, therefore, provides a direct explanation for the controversy in the field regarding the diverse contributions of glutamatergic PPN neurons to movement control.

Given the implication of the PPN in the pathogenesis of Parkinson’s disease (PD), our findings could potentially have translational value. The PPN has been used as a target in deep brain stimulation approaches to ameliorate PD symptoms with variable outcomes^68–72^. Based on recent findings from our group^28,30^ and others^29,71^, in combination with the insights from the present work, it is likely that a successful approach for deep brain stimulation targeted to the PPN to alleviate PD locomotor dysfunctions should avoid the rostral part of the nucleus to prevent the engagement of the Chx10^+^ population. Instead, it should aim to engage the caudal glutamatergic neurons (mostly Vglut2^+^/Chx10^−^), which comprise the majority of glutamatergic neurons in the PPN, have a locomotor-promoting role^26–30^ and have already been shown to ameliorate gait deficits in parkinsonian animal models^30,71^.

## Methods

### Experimental animals

#### Mice

All animal experiments and procedures were performed in laboratory mice (*Mus musculus*), carried according to the EU Directive 2010/63/EU, and approved by the Danish Animal Experiments Inspectorate (Dyreforsøgstilsynet, license no. 2017-15-0201-01172) and the local ethics committee at the University of Copenhagen.

For all behavioral experiments targeting the PPN, we used hemizygous *Chx10*^*Cre*^ mice (same strain as previously reported^7,35^). For targeting the vlPAG, we crossed hemizygous *Chx10*^*Cre*^ mice with the homozygous conditional *R26R*^*ChR2−EYFP*^ line (stock no. 012569, Jackson Laboratories). For anatomical studies, we crossed hemizygous *Chx10*^*Cre*^ mice with the homozygous conditional reporter lines *R26R*^*EYFP*^ or *R26R*^*tdTomato*^ (stock no. 006148 and 007905, respectively, Jackson Laboratories). All experiments were performed in adult (>8 weeks) male or female mice (randomly selected, approximately 1:1) kept on a 12-h light–dark cycle with access to food and water ad libitum (housing temperature 23–24 °C, 45–65% humidity).

### Surgical procedures, implants and optical stimulation parameters

#### Stereotaxic injections

Viral injections were performed using a motorized stereotaxic injection system (StereoDrive Robot Stereotaxic, Neurostar). Mice were anesthetized with isoflurane (induction 4%, maintenance 2.0–1.5%; Link 7 Anesthesia & Evacuation System, Patterson Scientific) and head-fixed in the motorized stereotaxic frame (model 900SD, Kopf Instruments). After a skin incision to expose the skull, a craniotomy was performed using a hand-held drill (Success 40, Osada). A total volume of 80–100 nl of virus solution was injected into the target region at a rate of 100 nl min^−1^ using a wireless glass-capillary nanoinjector (NeuroW, Neurostar) with a pulled glass micropipette (~30-μm tip diameter; glass capillaries, Neurostar). The glass micropipette was kept in place for 5–8 min following the injection to prevent backflow. Body temperature was maintained at 37 °C throughout the procedure with a feedback-controlled heating pad (Rodent Warmer X1, Stoelting). After completion of surgery, buprenorphine was subcutaneously administered to alleviate pain (0.1 mg per kg body weight). Viral vectors and coordinates used in this study are listed within each experiment.

#### In vivo optogenetics

Optical fiber implants were assembled in-house by coupling bare optical fiber (multimode fiber, 0.22 NA, core diameter of 200-µm; Thorlabs) into a ceramic ferrule (diameter of 1.25 mm; Thorlabs) with epoxy (F112, Thorlabs), followed by multiple polishing steps and cleaving of the bare fiber to achieve the desired length for the target brain area.

For in vivo optogenetic activation of the PPN, *Chx10*^*Cre*^ mice were unilaterally injected with 80–100 nl AAVdj-EF1a-DIO-hChR2(E123T/T159C)-p2A-mCherry-WPRE or a control virus (AAVdj-EF1a-DIO-EYFP-WPRE), both a gift from K. Deisseroth. During the same surgery, an optical fiber was implanted and secured to the skull with ultraviolet light curing bonding agents and dental cement (primer and adhesive: OptiBond FL, Kerr; dental cement: Tetric EvoFlow, Ivoclar Vivadent). All unilateral injections and implantations targeted the right hemisphere. For in vivo optogenetic activation of the vlPAG, *Chx10*^*Cre*^; *R26R*^*ChR2−EYFP*^ mice, which constitutively express ChR2 in Chx10^+^ neurons, were unilaterally implanted with an optic fiber using the same procedure. All the injections and implantations were performed with a −20° angle on the sagittal plane at the following coordinates relative to bregma: for PPN, AP = −4.36 mm, ML = 1.25 mm, DV = 3.65 mm; for vlPAG, AP = −4.72 mm, ML = 0.55 mm, DV = 2.80 mm. Optical fibers were implanted with the same angle, trajectory, AP and ML coordinates, but with an end position of the fiber tip 400–500 µm above the DV injection target. Behavioral experiments started approximately 4 weeks after injection/implantation to allow for optimal opsin expression.

For activation of ChR2-expressing neurons, blue light (473 nm laser, OptoDuet, Ikecool Corporation) was delivered in trains of 10-ms pulses at 40 Hz (Master-8 or Master-9 pulse generator, A.M.P.I.) through a patch cord connected to the chronically implanted ferrule with a ceramic mating sleeve. Total train duration varied between 1 and 3 s depending on the experiment. In specific trials, long stimulation trains (up to 20 s) were delivered to assess the maximum duration of the opto-evoked phenotype. Before behavioral testing, all mice were screened for their response to blue light. Laser power was individually adjusted to the minimum intensity effective to evoke a consistent behavior, typically ranging between 2.5 and 15 mW, measured at the tip of the patch cord that connects to the implanted ferrule. We defined a consistent behavior as a motor arrest (no limb or head movements) time-locked to the duration of light stimulation that is observable by eye, without looking at respiration or any other parameters. The chosen laser power for each mouse was kept constant between trials and experiments. We used two types of control experiments for optical stimulation: (1) in the cylinder test, a separate group of mice were injected with a control virus expressing a fluorophore without opsin while everything else was kept equal to the ChR2-expressing mice, including stimulation parameters; (2) in all plethysmography-involving experiments, all the ChR2-expressing mice were used as their own controls by being tested both with blue (473 nm) and yellow (593 nm) light (473/593 nm dual wavelength laser, OptoDuet, Ikecool Corporation).

The optically evoked phenotype was subsequently assessed in several behavioral assays described below.

#### Electrode implantation for chronic limb electromyography recordings

When applicable, mice expressing ChR2 in the PPN (‘In vivo optogenetics’) were implanted with electrodes in one or both hindlimbs to record the activity of the tibialis anterior and soleus muscles. Mice were anesthetized with isoflurane (induction 4%, maintenance 2.0–1.5%) and placed on a heating pad. Recording electrodes were built in-house and disinfected before implantation. The implantation area on the hindlimbs was shaved and disinfected, small incisions were performed to expose the muscles, and two Teflon-coated stainless-steel wires (790700, A-M Systems) free of coating on the wire tips were inserted in the belly of each muscle for bipolar recordings. The wires were routed subcutaneously to a connector placed on the back of the animal. All incisions were closed up with suture stitches. After surgery, buprenorphine was administered to alleviate pain (0.1 mg per kg body weight, subcutaneously) and animals were closely monitored for signs of discomfort. Mice started behavioral experiments not earlier than a week after surgery.

#### Telemetry sensor implantation for electrocardiography recordings

When applicable, mice expressing ChR2 in the PPN or vlPAG (‘In vivo optogenetics’) were chronically implanted with a telemetry sensor (easyTEL-S-ETA, Emka Technologies) to monitor heart rate in unrestrained freely moving conditions. Before surgery, implants were prepared as follows: the two wires were cut to the desired length, the coating was removed at the tip, and a small loop was made. Implants were disinfected before implantation. Mice were anesthetized with isoflurane (induction 4%, maintenance 2.0–1.5%) and kept on a heating pad. The implantation areas were shaved and disinfected, and three incisions were performed: one on the back to place the sensor subcutaneously, and two on the left and right sides of the chest. The wires were routed subcutaneously from the back to the chest and stitched to the muscles by the wire loops. All incisions were closed up by suture stitches. After surgery, buprenorphine was administered to alleviate pain (0.1 mg per kg body weight, subcutaneously) and animals were closely monitored for signs of discomfort. Mice started behavioral experiments not earlier than a week after surgery.

### Motor behavior

#### Linear corridor test and analysis

We assessed the effect that optical stimulation of Chx10-PPN or Chx10-vlPAG neurons has on spontaneous locomotion using a linear corridor (transparent plexiglass; 120 cm long, 9 cm wide and 12 cm high; MotoRater 303030 series, TSE Systems). Videos were acquired at 300 frames per second (f.p.s.) with the in-built software of the MotoRater using a high-speed camera (CL600x2/C/FM, Optronis) with a Distagon T* 2/35 ZF.2 lens (Carl Zeiss) located below the corridor, which simultaneously records three views (ventral, left and right) through a mirror system. The camera was set at a fixed position below the center of the runway capturing a 55-cm-long view of the corridor. Mice tethered to the laser through a patch cord were free to cross the corridor at their desired speed. Optogenetic stimulation (blue light, 1 s, 40 Hz; ‘In vivo optogenetics’) was manually triggered when mice were midway through the corridor. Only trials with at least 500 ms of trackable video (that is, bottom view of the mouse within the field of view of the camera) before and after light were considered for analysis. Videos were analyzed by tracking the perianal area of mice from the bottom view, using the pattern-matching algorithm from the two-dimensional tracking tool of TSE Motion software (v. 8.5.8, TSE Systems). The first light on frame of each trial was used for temporal alignment and two-dimensional coordinates over time were exported for further processing with custom Python scripts.

Position along the chamber (*x* coordinate) was first offset corrected and then downsampled to 150 f.p.s. Velocity (m s^−1^) was calculated by dividing the difference in chamber position (*x* coordinate, meters) by the difference in time (s) between samples (*v* = (*x*_*t*2 _− *x*_*t*1_)/(1/*sf*)), where *sf* is 150 f.p.s. Finally, the jitter resulting from tracking artifacts at high sampling rate was filtered out by setting velocity values below 0.045 to 0. The group average velocity was obtained by averaging all trials, while the mouse average velocity was obtained by averaging all trials of each mouse. The mouse average velocity was smoothed with a centered rolling average (window size 20 ms) for illustration purposes only. Further analysis (heat maps, latencies) was performed using non-smoothed single-trial data. Velocity heat maps were plotted using the ‘heatmap’ function of the Seaborn Python library, with the range of the colormap set to vmin = 0.0, and vmax = 1.0 (PPN) or 1.3 (vlPAG) m s^−1^, determined by computing the group average of the maximum speed found on each trial belonging to either of the experiments (PPN or vlPAG).

Latency to arrest locomotion was calculated as follows: first, the arrest time was defined as the first frame after light onset (time 0.0 s) where velocity equaled to 0.0 m s^−1^, only if that frame was within a sequence of four contiguous frames with zero velocity, and these were not followed by a four-frame sequence with non-zero values. The arrest time corresponded to the latency to arrest, since all trials were aligned with light onset as time 0.0 s. Latency to resume locomotion was calculated as follows: first, the resume time was defined as the first frame after light offset (time 1.0 s) where velocity was greater than 0.0 m s^−1^, only if that first frame was within a sequence of four contiguous frames with velocities greater than 0.0 m s^−1^. The latency to resume was then obtained by calculating the time difference between light offset and the resume time. The total time immobile was calculated by subtracting the arrest time to the resume time.

#### Cylinder test and analysis

The effect of optical stimulation of Chx10-PPN neurons on motor behaviors other than straight forward locomotion was tested on a cylindrical arena (transparent plexiglass; 20 cm in diameter, 30 cm high), where mice are more prone to spontaneously exhibit behaviors such as grooming or rearing intermingled with ambulation bouts. We defined ambulation broadly as walking/exploring in the cylindrical arena, not necessarily following a straight path. Mice were tethered to the laser through a patch cord with an integrated rotary joint (RJPFL2, Thorlabs) and video monitored (side view) in the cylinder during a 15-min session. The arena was illuminated with an infrared (IR) illuminator (RM25-120, rayTEC). Side-view videos were recorded at 25 f.p.s. (1,280 × 1,024 resolution) using a GigE monochrome camera (acA1300-60gm, Basler), with a lens (H3Z4512CS-IR, Computar) coupled to an IR pass filter (Heliopan). The recording session was acquired with and controlled by EthoVision XT (v. 15, Noldus Information Technology) to perform online activity analysis during acquisition (see below). Optogenetic stimulation (blue light, 3 s, 40 Hz; ‘In vivo optogenetics’) was triggered by the experimenter when mice performed any of the three behaviors (ambulation, grooming, rearing). Stimulation events were annotated offline to classify each event into one of the three behavior types. The behavioral classification reflects what the mouse was doing at the time of laser trigger, but not necessarily throughout the whole analysis window (9 s total). During data acquisition and annotation, the experimenter was blind to the treatment (ChR2 or control virus).

#### Activity analysis

Activity analysis is a real-time analysis feature of EthoVision XT (v. 15, Noldus Information Technology) where the grayscale values of all the pixels in the arena are determined upon acquisition of a sample and compared to the pixels of the previous sample to calculate the number of pixels that changed between the two. The activity analysis outputs the continuous variable ‘activity’, which represents the percentage of pixels changed between samples calculated as: activity = (CP_*n*_/*P*_*n*_) × 100), where CP_*n*_ is the number of pixels changed between the current and the previous sample, and *P*_*n*_ is the total number of pixels in the arena.

After behavior annotation, continuous activity data were exported for each recording session and analyzed with custom Python scripts. First, stimulation events were extracted from the continuous activity data and temporally aligned around light onset. A total of 251 stimulation events were analyzed (ChR2: *N* = 9 mice, *n* = 182 events; control: *N* = 3 mice, *n* = 69 events). Since mice performed the three behavior types (ambulation, grooming, rearing) randomly during the recording session, the number of stimulation events per behavior type varied across mice. On average, a total of 21 light stimulation events from any of the three behaviors were analyzed per mouse (minimum of 9, maximum of 29). One of the control mice did not perform any rearing events. For each behavior type within each treatment group (ChR2 versus control), the group average activity was calculated by averaging all stimulation events, while the mouse average activity was calculated by averaging all events from each mouse. The mouse average activity was then smoothed with a centered rolling average (window size 160 ms) for illustration purposes only. Further calculations were performed using non-smoothed data.

#### Percentage of time active

To classify the continuous activity into activity states (active versus inactive), we first defined an inactivity threshold (see next paragraph for details) above which mice were classified as being active. For each stimulation event and regardless of behavior type, a time window of 9 s was considered, divided into three epochs of equal length (3 s; before, during and after light on). We then applied the inactivity threshold to the raw activity data, which classified each sample as active or inactive, and computed the percentage of samples above threshold (that is, active) within each of the three epochs for each stimulation event. We then calculated the mouse average (time active, percentage) for each epoch by averaging all stimulation events of a mouse, which reflects the percentage of time that each mouse spent being active within each of the 3-s epochs. Data are summarized with boxplots divided by treatment group (ChR2 versus control) within each epoch.

The activity values in Figs. 2f and 4a,f are labeled as arbitrary units instead of percentages because the absolute values of continuous activity in percent and, therefore, the inactivity threshold, depend on the video resolution and on the size of the subject relative to the arena. Thus, these values are not necessarily comparable across experiment types (that is, cylinder versus plethysmograph). However, the intra-experiment range of activity values and thresholds are comparable and kept constant, as the same recording setup was kept for all the sessions within each experiment type. Optimal inactivity thresholds for each experiment type (cylinder or plethysmograph) were determined offline by the experimenter after inspecting video segments outside the stimulation time windows from a subset of videos randomly picked from each experiment type. The threshold was kept constant for all animals within each experiment type.

### Chronic limb electromyography recordings

To study the impact of Chx10-PPN neuron activation on limb muscles, activity from the tibialis anterior and soleus muscles was recorded in one or both hindlimbs from awake animals during locomotion before, during and after optogenetic stimulation. Electrodes were chronically implanted as described in ‘Electrode implantation for chronic limb electromyography recordings’. The small diameter and flexibility of the wires allowed mice to perform natural locomotor movements. Recordings were performed while mice walked freely in a linear corridor tethered to the laser through a patch cord (same as in ‘Linear corridor test and analysis’). Optogenetic stimulation (blue light, 1-s or 2-s trains, 40 Hz; ‘In vivo optogenetics’) was manually triggered when mice were midway through the corridor. EMG signals were amplified and band-pass filtered (100 Hz to 1 kHz) using a low-noise pre-amplifier and amplifier system (custom made at the Electronics Lab of the Büschges group, University of Cologne), acquired at 5 kHz (AxoScope 10.6, Molecular Devices) and digitized (Digidata 1440A, Molecular Devices) for offline analysis. Optical stimulation was controlled with a Master-8 pulse generator (A.M.P.I.) also connected to the EMG recording system for synchronizing all signals. Data were inspected using Spike2 (v. 7.06, Cambridge Electronic Design), and raw example traces were exported for graphical representations.

### Limb dynamics

#### Hindlimb kinematics

During limb EMG recordings, side-view videos were simultaneously recorded in some of the trials. Videos were acquired at 200 f.p.s. using the same setup described in ‘Linear corridor test and analysis’ and the same optogenetic stimulation parameters as described in ‘In vivo optogenetics’. Side-view videos were analyzed using TSE Motion software (v. 8.5.8, TSE Systems) to track six hindlimb points: iliac crest, hip, knee, ankle, metatarsophalangeal joint (MTP) and toe tip. The hindlimb tracking was used to generate stick diagrams that represent hindlimb kinematics. The video and EMG recordings were not time synchronized during acquisition. Therefore, hindlimb position data generated with the TSE Motion software were exported and further analyzed with custom Python scripts to incorporate temporal information into the stick diagrams and synchronize them with the EMG signal. The synchronization of the limb kinematics with the EMG recordings was achieved by using the optical stimulation as a reference. The onset of optical stimulation was treated as the zero-reference, *t*_zero_, to which the EMG signal and the stick diagrams were aligned for temporal coordination. Successive stick diagrams extracted from each frame were then plotted with a spacing corresponding to the inter-frame interval, *t*_frame_ = 1/*sf*, where *sf* is the number of frames per second in the video. The time-synchronized kinematic visualization captures the temporal progression of the limb kinematics, and also provides a way to align the stick diagrams with the EMG signal throughout the entire execution of the step cycle (Fig. 3a).

#### Hindlimb position during arrest

All trials in the linear corridor experiment (‘Linear corridor test and analysis’) were further analyzed to study the hindlimb position during arrest. For each trial, we extracted a representative video frame to identify the arrest position during light stimulation once the animal was completely immobile. On each arrest frame, we measured the left and right hind paw area in contact with the floor to determine whether the paw was in mid-stance phase. The mid-stance phase was defined as the period of the step cycle where the paw-to-floor contact area constituted more than 25% of the maximal paw area. The maximal paw area was computed as the average area from all trials in full contact and, thus, excluded early touch down in the beginning of the stance and late stance where the paw is close to be lifted off the ground. In the trials were both hind paws were in stance, we computed the angle between the line across the body axis excluding the head and the line between the two hind paws. We defined a perpendicular (‘aligned’) stance phase as a stance phase where the angle between the body axis and the two hind paws was 90° ± 15°. This corresponds to both hind paws being aligned on the same virtual vertical line and perpendicular to the body axis. Both the paw area and hind limb-to-body axis angle were computed using the Fiji distribution of ImageJ (ImageJ2 2.9.0; Java 1.8.0_322). The same procedure was used for Chx10-PPN and Chx10-vlPAG stimulation trials.

#### Limb coordination

All trials in the linear corridor experiment (‘Linear corridor test and analysis’) were further analyzed to study inter-limb coordination. We used the bottom view to track the four paws using DeepLabCut (DLC)^73^, followed by further processing with custom Python scripts (see steps below).

#### Pose estimation

As a first step, the positions of the four limbs (paws, as seen from the bottom view) were extracted for each frame of the bottom-view videos using DLC, a deep learning-based markerless pose estimation tool. We first selected a total of 100 random video frames from the bottom-view videos (*n* = 40). Six markers were used in each frame: **p** = (**p**_LF_, **p**_RF_, **p**_LH_, **p**_RF_, **p**_B_, **p**_M_) corresponding to the left forelimb (LF), right forelimb (RF), left hindlimb (LH), and right hindlimb (RH), base of the tail (B) and midpoint of the trunk (M), respectively. Each marker in turn consisted of the *x*- and *y*-location in the frame. A ResNet-50-based^74^ DLC model was trained using these markers for a total of 5,000 training iterations. We used this trained model to predict on four of the videos, and corrected 32 of the erroneous frames obtained by using a threshold of *P* < 0.6 as reported by the tracking success (likelihood) scores reported in DLC. These corrections were incorporated into the original training set, resulting in an updated training set that now comprised 132 frames. Another ResNet-50-based DLC model was trained from scratch on this updated training set for 50,000 iterations. We found this iterative process, of training two DLC models for fewer number of training iterations, to be more useful than training a single model for a longer duration. The final DLC model was then used to predict the six markers, **p**, from all the 40 videos. These tracked markers returned as *x* and *y* coordinates from DLC were used for obtaining the speed and limb coordination estimates.

#### Speed estimation

The instantaneous tracking noise in tracked marker positions was first filtered using a moving window filter of 30 frames. From these filtered position estimates, the instantaneous horizontal translation speed (cm s^−1^) of the animal at any given frame number *k* was estimated as: ${s}_{i}=({p}_{i}^{k}-{p}_{i}^{k-1})/{t}_{frame}$, where *p*_*i*_ corresponds to the *x* coordinate of one of the four markers *i*, and *t*_frame_ is the inter-frame interval in seconds (*t*_frame_ = 1/*sf*). The speed estimates from all six markers [*s*_LF_*, s*_RF_*, s*_LH_, *s*_RF_, s_*B*_, s_*M*_] were averaged to obtain a speed estimate of the animal: $s=\frac{1}{6}{\sum }_{i}{s}_{i}({\rm{cm}}\,{{{\rm{s}} ^ {-1}}})$. Furthermore, the speed estimates of the two forelimbs (LFRF) and the two hindlimbs (LHRH) were averaged to obtain a separate speed estimate for each limb pair: *s*_*F*_ = (*s*_LF_ + *s*_RF_)/2 and *s*_*H*_ = (*s*_LH_ + *s*_RH_)/2 in cm s^−1^. This limb pair-specific speed information was used to detect the events of pause (speed zero) and play (speed non-zero) upon light stimulation for each limb pair (Fig. 3e).

#### Left–right limb coordination

To explore limb coordination dynamics, we measured the phase for two pairwise limb coordination profiles based on the four limb markers: forelimb coordination (LFRF) and hindlimb coordination (LHRH). For each coordination type, the phase difference was computed based on the relative peak displacement of the *x* coordinates between the two corresponding markers. Assuming a sinusoidal profile for limb displacement, the instantaneous phase for the forelimb coordination at time *t* is estimated as: *ϕ*_LFRF_ = 360*(*t* − *t*_0_)/*T*; degrees), where *t*_0_ is the time when the first limb peaks within a cycle, *t* is the time lag between the two limb markers, and *T* is the peak-to-peak duration (the locomotor cycle duration). The instantaneous phase for the hindlimb coordination was estimated using the same procedure. Maximum positive alternation corresponds to the phase of +180°, synchrony to 0°, and maximum negative alternation to −180° (Fig. 3c). The above-described phase measurement assumes that the animals move along the corridor in a straight line. To account for changes in the body axis direction, we adjusted the phase by measuring the angle of the body axis with respect to a reference horizontal line along the corridor, **r**. The body axis, **b**, is computed as the vector between the base of the tail (**p**_B_) and the trunk midpoint (**p**_M_) markers: **b** = **p**_M_ − **p**_B_. The angle between **b** and **r** is then used to estimate the body axis angle, ${\phi }_{{body}}=\arccos \left(\frac{\left(\bf b\cdot r\right)}{\left(\left|\bf b\right|\cdot \left|\bf r\right|\right)}\right)$, where || denotes the length of the vector. If the body axis is exactly aligned to the reference horizontal line then ${\phi }_{{body}}=0$. However, when the body axis makes a positive slope with respect to the reference line then the body axis angle is positive and negative if the slope is negative. This body axis angle was then either added to or subtracted from the estimated phase for each time step. Different coordination patterns during the arrest can also be intuitively displayed as a straight line between the two hindlimb markers (**p**_LH_, **p**_RH_) as shown in Extended Data Fig. 8f, where we also applied the body axis angle correction.

#### Step cycle continuity

A key observation from our experiments is that upon light activation of Chx10-PPN neurons, mice arrest locomotion at any phase of the step cycle with a short latency from light onset. The specific limb position during the arrest depends on the timing of laser trigger plus the latency. The posture then is kept on hold during light stimulation, and the step cycle resumes shortly after light offset, displaying what we call a pause-and-play pattern. To capture the fact that after light offset limb movement continues from the same position without postural rearrangements, we measured the phase difference by subtracting the phase measured at pause from the phase measured at play for each limb pair. Lastly, to quantify that the phase cycle continued as expected between the pause and play events in a more concrete manner, we introduced the notion of step cycle continuity. Our assumption for defining continuity was that if at the pause event the phase cycle was ascending with a positive slope, then at play event the phase should continue to have a positive slope, and the inverse if the phase was descending. In cases when at pause the cycle had already peaked (slope almost equal to zero), then at play the natural course of the cycle would be to switch and descend, or vice versa. For each of the limb pairs (LFRF and LHRH) in each of the videos, we quantified continuity as a binary outcome (1 = true, 0 = false) using the following criteria:

$${\mathrm{continuity}}=\left\{\begin{array}{l}1,{\mathrm{if}}\,{\mathrm{sign}}\left({m}_{\mathrm{pause}}\right)={\mathrm{sign}}\left({m}_{\mathrm{play}}\right)\\ 0,{\mathrm{if}}\,{\mathrm{sign}}\left({m}_{\mathrm{pause}}\right)\ne {\mathrm{sign}}\left({m}_{\mathrm{play}}\right)\\ 1,{\mathrm{if}}\,{\mathrm{abs}}\left({m}_{\mathrm{pause}}\right)\approx 0\,{\mathrm{and}}\left({\mathrm{sign}}\left({m}_{\mathrm{pause}}\right)\ne {\mathrm{sign}}\left({m}_{\mathrm{play}}\right)\right)\end{array}\right.,$$

where *m*_pause_ corresponds to the slope measured before the pause (that is, between the pause event and the previous peak/trough in the phase profile), *m*_play_ corresponds to the slope measured after the play (that is, between the play event and the subsequent peak/trough), ‘sign’ refers to the sign of the slope (positive or negative), and ‘abs*’* refers to absolute. The first two cases apply when the arrest happens during the ascending or descending phases, and only the sign of the slope (either *m*_pause_ or *m*_play_) is taken into account to determine the outcome. The third case applies when the arrest happens at the peak of the cycle (peak or trough) and requires two conditions to be fulfilled: first, that the absolute value of *m*_pause_ is nearly zero to classify a trial as being at the peak of the cycle (the sign does not matter as long as the absolute value is close to zero); second, that there is a sign switch between *m*_pause_ and *m*_play_. The same procedure was used for Chx10-PPN and Chx10-vlPAG stimulation trials.

#### Hardware and software

All processing of data regarding limb dynamics was performed using a desktop workstation running Ubuntu 20.04, with an Intel i7 processor, Nvidia RTX 3090 graphics processing unit and 32 GB memory. DeepLabCut (v. 2.1.8.2) was used for markerless pose estimation. Custom Python scripts were written in Python 3.8.5, using other scientific packages such as numpy (1.19.2), pandas (1.4.1), scipy (1.6.1), matplotlib (3.5.1) and seaborn (0.11.1).

### Respiratory and cardiac activity

We monitored respiratory and cardiac activity in three types of behavioral sessions: baseline, optogenetic intervention (blue or yellow light) and during anesthesia. In the sessions with anesthetized mice, we used acute intercostal EMG recordings to measure respiratory and cardiac activity without video monitoring the behavior. In all other cases, respiratory and cardiac activity were simultaneously monitored in freely moving unrestrained mice inside a whole-body plethysmography (WBP) chamber while performing online activity analysis as previously described in ‘Cylinder test and analysis’. The experimental setup (camera, video settings, illumination) and online activity tracking with EthoVision XT was similar to the cylinder test, except that mice were inside the WBP chamber instead of the cylindrical arena. During baseline sessions, mice were typically recorded for 18 min without any experimental intervention. During optogenetic sessions (blue or yellow light, 1-s or 3-s trains, 40 Hz), the laser was automatically triggered through EthoVision XT with a random interstimulus interval ranging from 45 to 90 s, but only if the instantaneous activity was higher than 0.1%, making sure that mice were not already fully immobile upon laser trigger. Mice used in optogenetic experiments went through the different sessions (baseline, blue, yellow) in three different days with a rest day in between. Care was taken to cover the light output around the patch cord/implanted ferrule junction to minimize the possibility of mice being disturbed by seeing the light and its potential effect on respiratory or heart rate.

#### Whole-body plethysmography (WBP)

Respiratory activity was monitored using a WBP for unrestrained mice modified for compatibility with optogenetic experiments (Emka Technologies). Flow changes (ml s^−1^) in the WBP are measured by a differential pressure transducer that has a port in the subject chamber and another port in the reference chamber. The pressure in the subject chamber changes with the animal’s breathing and is proportional to the subject’s respiratory flow. Downward deflections in the flow signal correspond to inspiration. The WBP was calibrated before each experimental session. Although mice were able to move freely within the closed plethysmography chamber, the small size of the chamber restricted the behavioral repertoire. Raw flow data (ml s^−1^) synchronized with the ECG and the video recording were analyzed with custom Python scripts. During analysis, we disregarded absolute flow values because our parameter of interest was the respiratory rate, which was computed by calculating the frequency of the peak inspiratory flow (PIF) events (‘Acquisition and analysis’).

#### Wireless electrocardiography

Cardiac activity was monitored using a wireless ECG system that consisted of a chronically implanted telemetry sensor (easyTEL-S-ETA, Emka Technologies; ‘Telemetry sensor implantation for ECG recordings’) and a receiver (easyTEL receiver, Emka Technologies) placed under the plethysmography chamber. This wireless setup allows for ECG recordings in freely moving animals, including during WBP recordings for simultaneous cardiac and respiratory monitoring. Raw ECG data (mV) synchronized with the respiratory flow and the video recording were analyzed with custom Python scripts.

#### Acquisition and analysis

WBP and ECG signals were acquired and digitized at 1,000 Hz using the easyMATRIX3 data acquisition device together with the IOX software (v. 2.10.5; both from Emka Technologies), and synchronized with the video recording and activity analysis of EthoVision XT using a Time Code Auxiliary Parity (TCAP) synchronization signal. Four inputs were acquired by the easyMATRIX3 acquisition system: (1) the ECG signal through the wireless receiver (*ecg*); (2) the WBP signal as an analog input through the differential pressure transducer (*dpt*); (3) TTL pulses reporting optical stimulation events when applicable (*stim*); and (4) the TCAP signal sent out from EthoVision XT (*tcap*), which is a synchronization signal with time information that allows for offline automatic synchronization of external data (in this case, *ecg*, *dpt* and *stim*) with the video recording within EthoVision XT. After acquisition, raw data were exported from the IOX software as an ASCII file and imported into EthoVision XT, which used the TCAP signal to temporally align all data with the video recording and the activity tracking performed online. All the raw time-synchronized data (activity, ECG, respiration, stimulation events) were then exported for further processing with custom Python scripts as described in the following sections.

Physiological data (*ecg*, *dpt*) were pre-processed before performing peak detection (beats, breaths, respectively) as follows: raw signals were first smoothed with a centered moving average (window size of 3 ms for *ecg*, 21 ms for *dpt*), followed by offset removal by subtracting the average of the whole recording from each sample. Events (peaks) were detected using the ‘find_peaks’ function of the SciPy Python library, which finds local maxima based on peak properties. The appropriate parameters for each of the signals (height, prominence, distance, width) were predefined using a subset of recordings, and kept constant through all recordings of the same experiment type. We defined an event (beat) on the *ecg* signal as the R wave of the QRS complex. On the *dpt* signal, however, an event (breath) was defined as the PIF. Because inspiration in the original *dpt* signal corresponds to downward deflections, PIF’s correspond to local minima, requiring the input *dpt* signal to the ‘find_peaks’ function to be inverted to find PIF’s as local maxima.

#### Raster plots and peri-stimulus time histograms

Respiratory and heart rate data are presented with raster plots and PSTHs for all optical stimulation trials recorded across the different sessions (blue light, yellow light), different light stimulus durations (1 or 3 s) and different stimulation targets (Chx10-PPN, Chx10-vlPAG). Both are displayed within a time window centered around light onset that contains the whole stimulus duration, 3 s before light, and 3 s after light. The mean rates (breaths per second –Hz– for respiration, bpm. for heart), as shown in the PSTHs, were calculated using 100-ms bins for all trials included in each raster.

#### Maximum change

To determine the maximum change in rate (heart or respiratory) during the light stimulation period compared to the baseline rate, we first calculated the continuous mean rate for each mouse, using all the trials belonging to individual mice and the same bin size as in PSTHs. The mouse mean rate was then smoothed with a centered moving average (window size of 3 bins, 300 ms). For each mouse, we established the baseline rate to be the average rate of the 5 s before light onset and assigned that value to be 100%. We then searched for the minimum rate found within the whole light on period plus 500 ms after light offset (that is, within 1.5 s from light onset for 1-s stimulations, and within 3.5 s for 3-s stimulations) and computed the percentage of the baseline rate that the detected minimum rate corresponds to. We included 500 ms after light off in the search window to account for the slow dynamics in heart rate changes, where the maximal drop is often observed toward the end of the 1-s stimulation or even right after. Finally, we calculated the percentage change between the two, to obtain the maximum rate drop per mouse and visualized it using box-and-whisker plots, where 0% of maximum change corresponds to no change from baseline, while 100% corresponds to a drop to zero breaths or beats for at least 100 ms considering all stimulation trials of a mouse together.

#### *z*-scores

*z*-scores for respiratory and heart rate were computed from the continuous mean rates of each mouse using the mean and s.d. from the baseline period (5 s before light onset) of each mouse and smoothed with a centered moving average (window size of 3 bins, 300 ms) for visualization.

#### Epoch averages

Epoch averages (5 s before, 1 or 3 s light on, 5 s after) shown in Extended Data Fig. 6 for blue and yellow light trials were computed from the continuous mean rate of each mouse as above but without smoothing.

#### Baseline sessions

The initial pre-processing and peak detection was performed in the same way as in optogenetic sessions. In the absence of experimental intervention, we searched for naturally occurring apneic arrest events as putative Chx10-PPN-triggered arrest bouts, under the assumption that this combination of motor and respiratory features reflects the naturally occurring behavior. As a first step, we searched for apnea periods, and validated a natural event as true if it simultaneously showed motor arrest. We defined apnea as the absence of PIFs for at least 500 ms, which corresponds to the 99th percentile of all inter-PIF-intervals recorded in baseline conditions (all baseline sessions from all mice pooled). Within each apnea event, where the onset is the last PIF before a gap of at least 500 ms and the offset is the first PIF after, we computed the percentage of time that mice spent below the inactivity threshold (similar to ‘Cylinder test and analysis’). Only apnea periods where mice spent at least 80% of the time in an inactive state were considered as true apneic arrest events. This criterion allowed us to automatically detect naturally occurring apneic arrest bouts of varied durations throughout the entire baseline recording sessions. To obtain a more homogeneous set of natural events comparable in length to the optogenetically evoked ones (1-s stimulation), we further selected the events where the apnea periods were longer than 800 ms. To estimate the putative onset of the natural event preceding the last PIF, which presumably is a natural trigger that evokes the arrest with certain latency, we generated random latencies with a uniform distribution over a time range spanning the mean latency to arrest ± s.d. found in the linear corridor experiment. We then took all apnea onsets (last PIF before a gap of at least 800 ms where mice are simultaneously inactive) and subtracted one of the randomly generated latency values to obtain a putative onset or ‘natural trigger’ time. From this point on, data were treated similarly to optogenetic sessions to calculate the continuous smooth mean rates and maximum changes.

#### Acute electromyography recordings in anesthetized mice

To assess if the changes in respiratory and cardiac activity during Chx10-PPN activation were direct or secondary to the opto-evoked motor arrest, acute intercostal EMG recordings were performed in anesthetized animals. A batch of ten mice expressing ChR2 in the PPN and implanted with an optical fiber (as described in ‘In vivo optogenetics’) were anesthetized with ketamine/xylazine (87.5/12.5 mg per kg body weight) and kept on a heating pad. An incision was performed on the thorax and two Teflon-coated stainless-steel wires (790700, A-M Systems) were inserted in an intercostal muscle for bipolar recordings. Respiratory and ECG signals were simultaneously recorded through the same electrode. In two of the ten animals, only the intercostal muscle activity but not the ECG signal was captured by the recording electrodes. The recording hardware and software, and the stimulation parameters were the same as described in ‘Chronic EMG limb recordings’. Two-second-long trains of optical stimuli (40 Hz, 10-ms width pulses) were delivered every 10 s and changes during stimulation in respiratory frequency (*N* = 10 animals, *n* = 270 trials) and heart rate (*N* = 8; *n* = 237) were analyzed using Spike2 (v. 7.06, Cambridge Electronic Design). The raw EMG signal was first filtered to separate the heart from the respiratory signal based on their distinctive features. The respiratory signal was then rectified. Events (heart beats, respiratory bursts) were detected as local maxima within the heart signal and rectified respiratory signal, respectively. Events were further analyzed with custom Python scripts similarly to plethysmography data (‘Raster plots and PSTHs’). Example EMG traces were exported with Spike2 for graphical representations.

#### Short stimulation trains

To investigate the effect of Chx10-PPN activation on the respiratory rhythm, we applied short (250 ms) stimulation trains (40 Hz; 10-ms pulse width), which were delivered randomly throughout the respiratory period (*N* = 8; *n* = 293). The respiratory period (*P*) is defined as the time between the onset of two consecutive inspiratory bursts, while the expiratory phase is defined as the time from the offset of an inspiratory burst to the onset of the next inspiratory burst. To evaluate any changes in the respiratory phases caused by the stimulation, we constructed a phase-response curve. The *x* axis in the phase-response curve represents the normalized respiratory period (*P* = 0.0–1.0) of the period perturbed by the light stimulus. The inspiratory phase extends approximately from 0.0 to 0.3, while the expiratory phase extends between 0.3 and 1.0. The *y* axis shows the value of the phase shift in the respiratory rhythm (*R*) happening in the perturbed period *P*. It is calculated as *R* = *P/(P*_1_*)* + *(P*_+1_*)*, where *P* is the respiratory period in which the stimulation was delivered and *P*_−__*1*_ and *P*_*+1*_ are the periods before and following *P*, respectively. If the duration of the periods during and around the stimulation is similar (that is, *P*_−__*1*_ ≈ *P* ≈ *P*_*+1*_), *R* will be around 0.5, which we extended to between 0.4 and 0.6 to account for small variations in the respiratory cycle. R values in this range mean that the stimulation did not affect the respiratory rhythm. When the perturbed period *P* is prolonged relative to *P*_−__*1*_ and *P*_*+1*_, *R* values range between 0.6 and 1.0. That happens when the rhythm is perturbed with the stimulation leading to either a phase advance of the following burst followed by resetting of the respiratory rhythm, or a phase delay of the following burst without resetting.

### Loss-of-function experiments

#### Ablation strategy

To ablate Chx10-PPN neurons, *Chx10*^*Cre*^; *R26R*^*tdTomato*^ mice were bilaterally injected in the PPN (same coordinates as for ChR2 infections) with 150 nl of either AAV5-hEF1α-FLEX-(pro)taCasp3-2A-TEVp (Addgene, 45580) or AAV5-hEF1α-FLEX-EYFP as a control (Zurich Viral Vector Facility; refs. v185-5 or v343-5, respectively). Behavior was recorded before surgery (naïve mice) and 5 weeks after surgery, to allow for Casp3-induced apoptotic cell death of the infected Chx10-PPN neurons^43^. After completion of behavioral experiments, mice were euthanized and their brains were collected, cut on a cryostat in 30-µm coronal sections, and stained as described in ‘Tissue processing and immunohistochemistry’. Serial coronal images covering the rostrocaudal axis of the PPN of all mice were acquired using an AxioScan 7 automated widefield fluorescence slide scanner with a ×10/0.45 objective (Zeiss). The use of *Chx10*^*Cre*^; *R26R*^*tdTomato*^ reporter mice allowed us to assess the extent of the ablation as follows: we first mapped the coronal images and their corresponding level in the mouse brain atlas. We then selected the same coronal level for all mice, approximately at −4.36 mm from bregma, where Chx10 neurons are enriched in the PPN. Chx10-tdTomato^+^ cells within the PPN were quantified bilaterally to obtain the total count per mouse using the Fiji distribution of ImageJ (ImageJ2 2.9.0; Java 1.8.0_322). Data are represented per mouse within each treatment group (Casp3, control), where each circle corresponds to the total Chx10-tdTomato^+^ count of a mouse (one section per mouse; *N* = 8 mice per group).

#### Open field (OF) test and analysis

Control EYFP mice and Casp3-mice were tested in a cylindrical OF arena (40 × 40 cm) in three sessions of 20 min each. Two baseline sessions were recorded consecutively (2 d apart) with the 16 naïve mice before surgery, and one post-ablation session (*N* = 8, Casp3-injected mice) or post-injection session (*N* = 8, control-injected) was recorded 5 weeks after surgery. The experimenter was blind to the treatment groups throughout. Mice were video monitored from the top view using the same acquisition system as for the cylinder test. Videos were acquired at 50 f.p.s. The recording session was controlled by EthoVision XT (v. 15, Noldus Information Technology) to simultaneously perform online activity analysis during acquisition (‘Activity analysis’). To detect arrest events, we used the activity analysis instead of speed-based criteria because the activity analysis is sensitive to any change from frame to frame regardless of full-body displacement occurring or not and, thus, provides a more sensitive readout of full immobility. The optimal inactivity threshold for this setup was determined by looking at test recordings with non-experimental mice. We only considered arrest events (mouse activity below inactivity threshold) between 500 ms and 2 s, to avoid potential confusion with longer arrest bouts that could be freezing or other kinds of long immobility bouts. For each OF session, we calculated the total amount of arrest events that fell into this criterion. We then averaged the amount of arrest events of the two baseline sessions recorded for each mouse to obtain a single baseline count. Changes in the amount of arrest events (baseline versus post) are evaluated and displayed in figures normalized to the baseline of each mouse (that is, percentage of baseline or percentage change from baseline, where the baseline of each mouse is 100%).

### Conditioned place aversion

To test whether mice perceived the light activation of Chx10-PPN or Chx10-vlPAG neurons as aversive, we used a conditioned place aversion paradigm, where we analyzed whether mice changed their initially preferred side of the chamber after this side was paired with optical stimulation (PPN: *N* = 6 mice with blue light and *N* = 5 mice with yellow light control; vlPAG: *N* = 6 mice with blue light). Mice were tested in a square OF arena (50 cm × 50 cm) with opaque floor and walls that was divided into two equal sides (chambers) by a wall with an opening of approximately 4.5 cm, allowing mice to freely explore both sides. The two chambers had distinct visual and tactile characteristics allowing mice to identify them as different and develop preference. During the conditioning sessions, the central wall was replaced by a true wall without any opening, leaving mice confined to one of the sides.

The conditioning paradigm was run across 3 d and consisted of four experimental sessions of 30 min each: On day 1 (pre-test), mice were tethered to the laser and free to explore both sides of the arena without receiving any stimulation. On day 2 (optogenetic conditioning), mice underwent two behavioral sessions (morning and afternoon). In session 1, mice were confined to their preferred chamber and received optical stimulations (40 Hz, 1 s ON) repeated every 10 s over the 30 min that the session lasted, resulting in a total of 180 stimulation trains. In session 2, mice were confined to their non-preferred chamber, tethered to the laser, but did not receive any light stimulus. The conditioning sessions 1 and 2 were counterbalanced so that some mice were first confined to their preferred chamber and then to their non-preferred chamber, and vice versa. On day 3 (post-test), mice were again tethered to the laser and free to explore both sides of the arena without receiving any stimulation.

All sessions were video monitored from the top view using the same acquisition system as in other OF experiments. Data analysis was performed using the center-body tracking within Ethovision XT v.15 (Noldus Information Technology). We first determined the percentage of time spent in each of the two chambers during the pre-test session to determine the preference of each mouse (that is, >50% of the time spent in a chamber). They all showed a mild preference for a specific chamber, ranging from 51% to 72% of cumulative duration. This information was then used to plan for the conditioning session, that is, pairing the preferred side with light or delivering no stimulation when confined to the non-preferred side, counterbalanced. After the post-test session, we again calculated the percentage of time spent in each of the two chambers. For each mouse, data are presented as the percentage of time spent in the preferred chamber during the pre-test (pre) and post-test (post). If light activation of Chx10 neurons is not perceived as aversive, there should not be a significant difference in the preference between pre and post. The heat maps showing the time spent across the entire chamber were exported directly from Ethovision and represent the mean over all heat maps (one heat map per mouse, per session).

### Histology, anatomy, image acquisition and analysis

#### Tissue processing and immunohistochemistry

Mice were deeply anesthetized with a lethal dose of pentobarbital (intraperitoneally) and transcardially perfused with a solution of ice-cold PBS and heparin (20 IU ml^−1^, Leo Pharma), followed by 4% paraformaldehyde in PBS (Histolab). Brains and, when necessary, spinal cords were dissected out and post-fixed for 3–4 h in 4% paraformaldehyde. Fixed brains were then rinsed in PBS and cryoprotected overnight with 25% (wt/vol) sucrose in PBS, frozen in Neg-50 embedding medium (Colorless Neg-50, Richard-Allan Scientific), sectioned on a cryostat (Microm HM550 or CryoStar NX70, Thermo Scientific), and mounted on Superfrost Plus slides (Thermo Scientific). Coronal slices were cut at a thickness varying between 14 and 40 µm depending on the final purpose.

Antibody staining of tissue sections was performed as follows: first, sections were rehydrated with PBS, permeabilized with 0.5% Triton X-100 in PBS (PBS-T; Sigma Aldrich), and blocked for 1 h with 5% normal donkey and/or goat serum (Jackson ImmunoResearch) in PBS-T. Second, sections were incubated overnight at 4 °C with one or several primary antibodies in a solution of 1% serum in PBS-T. Primary antibodies used in this study were rabbit anti-DsRed/tdTomato/mCherry (1:1000 dilution; 632496, Clontech/Takara), chicken anti-GFP (1:1,000 dilution; ab13970, Abcam) and goat anti-ChAT (1:100 dilution; AB144P, Millipore), and were incubated together with a fluorescent Nissl stain for anatomical reference (1:200 dilution; NeuroTrace Blue 435/455, N21479; or NeuroTrace Deep-Red 640/660, N21483, Invitrogen). Third, slides were washed with PBS-T and incubated for 2–4 h at room temperature in a solution of 1% serum in PBS-T with the appropriate fluorophore-conjugated secondary antibodies (1:500 dilution; Alexa Fluor 488 goat anti-chicken, Alexa Fluor 488 donkey anti-goat, Alexa Fluor 568 donkey anti-rabbit, Invitrogen). Finally, slides were washed and coverslipped using ProLong Diamond antifade mountant (P36961, Invitrogen) or Mowiol 4–88 mounting medium (Sigma Aldrich).

Images were acquired using a widefield fluorescence microscope (AxioImager.Z1), an AxioScan.Z1 or 7 automated fluorescence slide scanner, or an LSM 800/900 laser scanning confocal microscope (all from Zeiss).

#### Verification of viral infection and fiber placement

After completion of behavioral experiments, mice were euthanized and their brains were collected, processed (40-µm coronal sections) and stained as described in ‘Tissue processing and immunohistochemistry’. Tile scans of the coronal slices were acquired with a ×10/0.45 objective on either an AxioImager.Z1 or an AxioScan.Z1 widefield fluorescence microscope. For mice included in behavioral datasets, several slices regularly sampling the whole rostrocaudal axis of the target region were imaged and visually inspected to assess the extent of viral expression and optic fiber placement. Animals without visible viral expression in the target region and/or wrong fiber placement were excluded from analysis. In addition, we reconstructed the viral expression and optic fiber tip position for the behavioral group where optical stimulation of PPN was applied during ongoing locomotion (linear corridor) and other spontaneous motor behaviors (cylinder) as a representative dataset (Extended Data Fig. 2). Briefly, coronal sections were mapped to their corresponding level on the mouse brain atlas, and optic fiber position was assessed on the basis of the lesion produced by the fiber in the brain tissue. Considering the −20° angle on the sagittal plane used for injections and implantations, the last and most rostral image with a visible lesion was selected for each animal. The extent of viral expression was evaluated as previously described^28^.

#### Characterization of Chx10^+^ neuron distribution in the pedunculopontine nucleus

For characterizing the spatial distribution of Chx10^+^ neurons in the PPN, we used *Chx10*^*Cre*^; *R26R*^*tdTomato*^ conditional reporter mice, which express tdTomato in all Chx10^+^ neurons, combined with a ChAT immunostaining. Three reporter mice (*Chx10*^*Cre*^; *R26R*^*tdTomato*^) were euthanized, processed and stained (tdTomato-AF568, ChAT-AF488, NeuroTrace-640/660) as described in ‘Tissue processing and immunohistochemistry’. To regularly sample the whole rostrocaudal length of the PPN (approximately 800 µm), 30-µm-thick serial coronal slices were collected in four duplicate series, from the caudal end of the inferior colliculi to the first few caudal-most levels of the substantia nigra pars reticulata (from bregma: approximately −5.40 to −3.64 mm). For each mouse, one of the series (1 every 4th of all collected slices resulting in a 30 µm sample every 120 µm) was imaged using an AxioScan.Z1 automated widefield fluorescence slide scanner with a ×20/0.8 objective, a quadband filter set and a Zeiss AxioCam MRm camera. Each tile scan covering a full coronal slice was acquired as a *z*-stack of three levels with a 4-µm step. Stitching (10% minimum overlap using the NeuroTrace channel as reference) and orthogonal *z* projection (maximum intensity) were performed within Zen Blue software (Zeiss). Pre-processed images were then mapped into their corresponding level of the mouse brain atlas. Coronal sections containing the PPN nucleus were selected for further analysis according to the reference atlas by Franklin & Paxinos^33^ and characterized by the presence of ChAT^+^ neurons in the region (eight coronal levels in total; from bregma: caudal edge at −4.96 mm, followed by −4.84 mm, −4.72 mm, −4.60 mm, −4.48 mm, −4.36 mm, −4.24 mm and rostral edge at −4.16 mm). Regions of interest (ROIs) delineating the PPN (individual ROIs for left and right PPN within each section) were drawn visually aided by the Nissl (NeuroTrace) and ChAT staining, and the shape of the superior cerebellar peduncle (scp). ROIs were subsequently extracted as single files and the Intellesis Trainable Segmentation module of Zen Blue (Zeiss) was used for supervised machine learning-based segmentation (pixel classification by a random forest classifier). First, a multi-channel segmentation model with three classes (ChAT, Chx10, tissue) was trained using a manually labeled subset of ROIs as training dataset. Second, an interactive analysis pipeline was built using the previously trained segmentation model as an initial step, followed by several automated post-processing steps (for example, exclusion criteria based on minimum area for ChAT and Chx10 classes) and a final interactive step where the experimenter could assess the quality of the automated segmentation (all within Zen Blue, Zeiss). The analysis pipeline resulted in the segmentation of three classes per ROI: Chx10^+^ neurons, ChAT^+^ neurons and the whole tissue area of the ROI. On the same full-size images used for extracting the PPN ROIs, we also drew ROIs delineating the vlPAG and analyzed them using the same procedure for comparison.

#### Reconstruction of neuron distribution

For visualizing the actual spatial distribution pattern of Chx10^+^ neurons, seven hemisections of one mouse were selected (representative of the whole rostrocaudal axis of the PPN; from bregma: between −4.96 mm and −4.16 mm) and segmented as described above. Segmentation masks with the three objects of interest (Chx10^+^ cells, ChAT^+^ cells and the whole area of the PPN) were vectorized using the Image Trace feature of Adobe Illustrator. The same procedure was used for the vlPAG.

#### Density counts

For quantification of Chx10^+^ and ChAT^+^ neuron density, four hemisections were selected from each mouse (three mice in total; from bregma: −4.96 mm and −4.72 mm representing the caudal PPN, −4.48 mm and −4.24 mm representing the rostral PPN) and segmented as described above. Neuron counts and total ROI areas were exported, and neuron densities (neurons/mm^2^) were calculated for each cell type within each level of each mouse using custom Python scripts. The same procedure was used for the vlPAG.

#### In situ hybridization

Three conditional reporter mice (*Chx10*^*Cre*^; *R26R*^*EYFP*^) were euthanized and brains were processed as described in ‘Tissue processing and immunohistochemistry’. Fixed-frozen brains where kept at −80 °C and 14-µm-thick coronal sections were serially collected sampling the whole PPN. In situ hybridization for vesicular glutamate transporter 2 (Vglut2) mRNA was performed using the RNAscope Multiplex Fluorescent v2 Assay (323110, Advanced Cell Diagnostics, Bio-Techne). Pretreatment and in situ hybridization assay were performed following the manufacturer’s instructions for fixed-frozen tissue. Vglut2 mRNA was detected using the Mm-Slc17a6 hybridization probe in channel 1 (319171, ACD Bio-Techne), and the signal was amplified using TSA conjugated to Atto 550 fluorophore. The RNAscope protocol was immediately followed by immunohistochemistry against EYFP (ab13970, Abcam) as described in ‘Tissue processing and immunohistochemistry’ and nuclear counterstaining with DAPI (1:5,000 dilution). Tile scans of coronal slices were acquired with a ×20/0.8 objective on an LSM 800. A representative high-resolution *z*-stack was acquired with a ×40/1.20 water objective.

#### Coexpression analysis

For quantification of Chx10 and Vglut2 coexpression, four hemisections containing the PPN were selected from each mouse (*N* = 3, 12 hemisections total). Tile scans were stitched and ROIs delineating the PPN were drawn within Zen Blue software (Zeiss) as described in the previous section. Segmentation and coexpression analysis were performed using Imaris (v. 9.7.2; Bitplane, Oxford Instruments). Briefly, we first created a surface containing the PPN area based on the previously drawn ROIs and used the surface to mask all channels and perform the segmentation only within the PPN region. We then segmented Chx10^+^ neurons (EYFP^+^ on *Chx10*^*Cre*^; *R26R*^*eYFP*^ reporter strain) and Vglut2^+^ neurons (Vglut2 mRNA^+^) using the ‘spot detection’ function (constant spot diameter of 15 µm), and the automated segmentation results were subsequently curated by the experimenter. Finally, the Imaris XT ‘colocalize spots’ function was used for object-based colocalization. Colocalization/coexpression was defined to be true when the distance between the centroid of a Chx10^+^ neuron (spot) and a Vglut2^+^ neuron (spot) was smaller than 5 µm. We then computed two variables: the percentage of Chx10^+^ neurons among all Vglut2^+^ neurons within the PPN, and the percentage of Vglut2^+^ neurons among all Chx10^+^ neurons within the PPN. To generate the pie charts, first cell counts per hemisection (total Vglut2^+^, total Chx10^+^ and colocalized) were pooled to calculate mean coexpression percentages within each animal, and the group mean percentages were later calculated across animals.

#### Anterograde tracing

For anterograde tracing experiments, *Chx10*^*Cre*^ mice were unilaterally injected in the PPN (*N* = 4) or the vlPAG (*N* = 4) with 80–100 nl AAV1-phSyn1(S)-FLEX-tdTomato-T2A-SypEGFP-WPRE (Viral Vector Core, Salk Institute for Biological Sciences) using the same stereotaxic coordinates and injection parameters as in the optogenetic experiments. Mice were euthanized, processed and stained (tdTomato-AF568, SypEGFP-AF488, NeuroTrace-435/455) as described in ‘Tissue processing and immunohistochemistry’. Next, 30-µm-thick coronal brain slices were serially collected and imaged from the junction between the caudal medulla and the cervical spinal cord, up to the rostral pole of the hippocampus, approximately at −0.94 mm from bregma. The rostral-most ROI included in the quantification lies at −1.94 mm from bregma. Transverse spinal cord sections were also serially collected and imaged, including cervical, thoracic and lumbar segments. All images were acquired as tile scans using a ×10/0.45 objective on an LSM 900.

All coronal slices were first mapped into their corresponding level of the mouse brain reference atlas. Multiple atlas levels were then selected for analysis (same atlas levels for every mouse), resulting in a median distance of 240 µm between analyzed samples within each animal. For each slice, ROIs were drawn following Franklin & Paxinos’ reference atlas and guided by the Nissl (NeuroTrace) staining. We did not draw all structures present in each level of the reference atlas. Instead, we selected the structures to be considered as ROIs based on general relevance and presence/absence of labeling. The selected structures were the same for all mice. Afterwards, individual ROIs were extracted as single files and segmented using the Intellesis Trainable Segmentation module of Zen Blue (Zeiss), using a similar approach as in ‘Characterization of Chx10^+^ neuron distribution in the PPN’. Taking advantage of multi-channel information for pixel classification, we trained the segmentation model to classify pixels as putative boutons only if they had signal in both red (tdTomato) and green (sypGFP) channels, minimizing the chances that pixels with dirt or autofluorescence in the same range of brightness and size but restricted to a single channel could be wrongly classified as boutons. The analysis pipeline resulted in the segmentation of two classes per ROI: the boutons, and the whole tissue area of the ROI. Segmentation results were exported for further analysis with custom Python scripts (see below).

#### Quantification of projection pattern

To quantify the projection pattern, segmentation data from four *Chx10*^*Cre*^ mice injected in PPN was further processed as follows: first, for each unique ROI, we summed the number of boutons on the one hand, and the tissue area on the other, from all coronal levels where that ROI was present while keeping the ipsilateral and contralateral sides of each nucleus separated as individual ROIs. Second, we calculated the bouton density per ROI (boutons/mm^2^) dividing the total number of boutons by the total area. Third, we normalized the bouton density per ROI within each animal to a 0–1 range by min–max scaling as (*x*_norm_ = (*x* − *x*_min_)/(*x*_max _− *x*_min_), where *x*_max_ is the densest ROI in each mouse. ROIs containing the PPN, both ipsilateral (injection site) and contralateral, were excluded from this process. Data separated by side (ipsilateral/contralateral to injection site, or midline) are only shown for a selected group of brainstem structures, but were calculated and normalized taking into account all segmented ROIs except the PPN. For the extended overview of the projection pattern, we followed the same approach, but first summed the ipsilateral and contralateral sides within each level that belong to the same nucleus into a single ROI, and then summed across levels, calculated the density and normalized the bouton density by animal as explained above. In addition, we also calculated the fraction of output that goes into an ROI by dividing the number of boutons in that ROI by the total number of boutons segmented in that animal. As before, ROIs containing the PPN, both ipsilateral (injection site) and contralateral, were excluded from the process. PPN ROIs were assessed separately but applying the same normalization factor to each animal as previously used for the rest of the ROIs, making the results comparable to the ipsilateral and contralateral normalized bouton density values of the other ROIs. ROI names and acronyms are largely based in Franklin & Paxinos’ reference atlas, with few exceptions such as ‘PPN’ instead of ‘PTg’ (kept for consistency with our previous publications) and some children structures that have been merged into a single parent structure for conciseness (for example, LPGi and LPGiE as LPGi; PCRt and PCRtA as PCRt; Sp5, DMSp5, Sp5C and Sp5I as Sp5). Spinal cord images were not quantified as only very sparse labeling was found at high cervical levels and virtually no labeling in segments caudal to that.

#### Reconstruction of projection pattern

Images from six different brainstem levels were selected from a representative animal to reconstruct bouton positions. To keep spatial information, the segmentation (same model as above) was performed over images of full coronal slices without extracting the ROIs into single files. A bounding box around the tissue borders and a midline reference were manually added to transform spatial data into a meaningful set of *x* and *y* axes. Coordinates (*x*, *y*) of the centroid of the segmented objects (putative boutons) were plotted as single dots after transformation.

### Analysis software, statistics and data visualization

#### Analysis software

Data pre-processing and analysis of behavior, physiology and imaging were performed as described within each experiment. Apart from the explicitly mentioned commercially available or open-source tools related to each experiment type, we used Python (v. 3.7.4) and the following Python libraries throughout: Matplotlib (v. 3.3.2), NumPy (v. 1.19.2), pandas (v. 1.2.1), SciPy (v. 1.6.0), Seaborn (v. 0.11.2), except for the limb dynamics data (details specified in ‘Limb dynamics’). Details can also be found in the Nature Portfolio Reporting Summary.

#### Statistics

No statistical methods were used to predetermine sample sizes but our group sizes were similar to those used in the field^28,35^. Group sizes were typically smaller in anatomical experiments (3–4 mice) than in behavioral experiments (6–14 mice). Both male and female mice (approximately 1:1), littermates and non-littermates, aged between 2 and 8 months were randomly selected and allocated to experimental groups. The specific number of animals, trials, events or samples used on an experiment is included in each panel and/or the respective figure legend, the results section and the specific methods section associated with it. During behavioral data collection, the experimenter was blind to virus type if two different groups were used as treatment and control (for example AAV-DIO-ChR2 versus AAV-DIO-Fluorophore, or AAV-FLEX-Casp3 versus AAV-FLEX-EYFP), but was not blind to light wavelength (blue light versus yellow light) or injection target (PPN versus vlPAG) if a single group was used as its own control. However, data analysis was performed as automated as possible and using the same parameters regardless of group to avoid any influence on the outcome. In behavior experiments involving optogenetic stimulation, all mice were screened for their response to blue light before behavioral testing (‘In vivo optogenetics’). Mice that showed no motor phenotype upon blue-light stimulation (motor arrest visually assessed by the experimenter) were excluded from behavioral experimental groups before data collection. Postmortem tissue from mice included in behavioral and anatomical experiments was evaluated to assess injection site and fiber placement. Mice that had off-target or no viral expression were excluded from analysis.

In the text, data are described using the mean and standard deviation, range (minimum, maximum) and 95% CIs. Graphical data are displayed as raw and as complete as possible including all data points whenever possible. Point estimates are means unless otherwise stated. In continuous time-series data plots (for example, velocity and activity), thick lines are group means and thin lines are individual means summarizing all trials of an animal. In all box-and-whisker plots, lines indicate medians (Q2), box edges indicate the IQR where the lower edge is Q1 (25th percentile) and the upper edge is Q3 (75th percentile), and whiskers extend to the minimum and maximum excluding outliers (minimum = (Q1 – 1.5 × IQR); maximum = (Q3 + 1.5 × IQR)), but individual data points are always provided. If error bars or shades around point estimates are used in graphical representations, they correspond to either the standard error of the mean (mean ± s.e.m.; in for example, bouton density) or to the 95% CI (for example, ablation). Normality of data distributions was formally tested using the Shapiro–Wilk test and visually assessed with QQ plots. Data complying with the normality assumptions were tested using parametric tests. If any of the datasets within a plot did not comply with normality assumptions, the appropriate non-parametric alternative was used for all the comparisons within that plot. Two-group comparisons were performed using two-tailed Student’s *t*-tests with Welch’s correction (paired or unpaired, as appropriate), or Wilcoxon signed-rank or Mann–Whitney tests (non-parametric). For three or more groups and longitudinal data, one-way or two-way RM ANOVA was performed, followed by the appropriate multiple-comparisons test (Tukey’s after one-way RM ANOVA, Bonferroni’s after two-way RM ANOVA). In RM ANOVA, sphericity was not assumed and the Geisser–Greenhouse method was used to correct for violations. When indicated, we used multiple Mann–Whitney tests as the non-parametric alternative to the two-way RM ANOVA, followed by Holm–Sidak’s multiple-comparisons test. Reported *P* values are adjusted for multiple comparisons in all cases. When appropriate, details of the statistical approach used for each dataset can be found summarized in the figure legends and detailed in the text referring to them. Statistical significance was set at *P* < 0.05 and it is reported in text with exact values (except for values < 0.0001) with up to four decimal cases, while in figures it corresponds to *P < 0.05, **P < 0.01, ***P < 0.001, ****P < 0.0001 and NS when non-significant. All statistical analyses were performed using GraphPad Prism (v. 9.4.1).

#### Data visualization

Data plots were originally generated with custom-made Python scripts (v. 3.7.4) and rendered using standard plotting libraries (Matplotlib v. 3.3.2 and Seaborn v. 0.11.2). EMG traces were handled and exported using Spike2 (Cambridge Electronic Design) and microscopy images were handled and exported using Zen Blue 3.1 or 3.2 or Zen Lite 2.5 or 3.6 (Zeiss). In all cases, further aesthetic modifications, editing and figure assembly were done using Adobe Illustrator 2022 (v. 26.5).

### Reporting summary

Further information on research design is available in the Nature Portfolio Reporting Summary linked to this article.

## Online content

Any methods, additional references, Nature Portfolio reporting summaries, source data, extended data, supplementary information, acknowledgements, peer review information; details of author contributions and competing interests; and statements of data and code availability are available at 10.1038/s41593-023-01396-3.

## Supplementary information

Supplementary Information Supplementary Text, Supplementary Table 1 and Supplementary Video Descriptions.

Reporting Summary

Supplementary Video 1 Arrest of spontaneous locomotion in the linear corridor upon activation of Chx10-PPN neurons with blue light (1-s train at 40 Hz, 10-ms pulse width). Three examples. Playback speed 0.1×.

Supplementary Video 2 Arrest of other motor behaviors in a cylindrical arena upon activation of Chx10-PPN neurons with blue light (3-s train at 40 Hz, 10-ms pulse width). Three grooming examples, three rearing examples, and two ambulation examples. Playback speed 1× (real-time).

Supplementary Video 3 Pause-and-play pattern observable upon activation of Chx10-PPN neurons with blue light (3-s train at 40 Hz, 10-ms pulse width) during rearing and grooming. For each behavior, two examples are shown: first a ChR2-injected mouse and then an EYFP-injected control mouse. Playback speed 0.6×.

Supplementary Video 4 Arrest of spontaneous locomotion in the linear corridor upon activation of Chx10-vlPAG neurons with blue light (1-s train at 40 Hz, 10-ms pulse width). Two examples. The apparent mass over the lower back is an implanted wireless ECG sensor. Playback speed 0.1×.

## Data Availability

Source data are provided with this paper. Other data and material are available from the corresponding authors upon request.

## Source data

Source Data Fig. 1 Numerical/statistical source data.
Source Data Fig. 2 Numerical/statistical source data.
Source Data Fig. 3 Numerical/statistical source data.
Source Data Fig. 4 Numerical/statistical source data.
Source Data Fig. 5 Numerical/statistical source data.
Source Data Fig. 6 Numerical/statistical source data.

## Code availability

Custom code written to analyze limb dynamics is available at https://github.com/kiehnlab/pauseandplay and 10.5281/zenodo.8013911. Other custom code is available from the corresponding authors upon request.
