# Author Correction: Pedunculopontine Chx10^+^ neurons control global motor arrest in mice

**DOI:** 10.1038/s41593-023-01422-4

**Published:** 2023-08-11

**Authors:** Haizea Goñi-Erro, Raghavendra Selvan, Vittorio Caggiano, Roberto Leiras, Ole Kiehn

**Affiliations:** 1 Department of Neuroscience, Faculty of Health and Medical Sciences, University of Copenhagen, Copenhagen, Denmark; 2 Department of Computer Science, University of Copenhagen, Copenhagen, Denmark; 3 Department of Neuroscience, Karolinska Institutet, Stockholm, Sweden; 4 Present Address: Meta AI Research, New York, NY USA

**Keywords:** Central pattern generators, Motor control

Correction to: *Nature Neuroscience* 10.1038/s41593-023-01396-3. Published online 27 July 2023.

In the version of this article initially published, Vittoria Caggiano was not included in the author list for his contributions made while at the Karolinska Institutet. The error has been corrected in the HTML and PDF versions of the article.

