# Supplementary material for: Pedunculopontine Chx10+ neurons control global motor arrest in mice
Source: Nat Neurosci. 2023 Jul 27;26(9):1516–28. doi: 10.1038/s41593-023-01396-3 (PMC10471498; doi:10.1038/s41593-023-01396-3)
Supplement: Supplementary file 1 — Supplementary Text, Supplementary Table 1 and Supplementary Video Descriptions. [file 41593_2023_1396_MOESM1_ESM.pdf]

nature neuroscience

Article

https://doi.org/10.1038/s41593-023-01396-3

Pedunculopontine Chx10^{+} neurons control global motor arrest in mice

In the format provided by the authors and unedited

Pedunculopontine Chx10$^+$ neurons control global motor arrest in mice

Haizea Goñi-Erro[1], Raghavendra Selvan[1,2], Roberto Leiras[1], & Ole Kiehn[1,3]

[1]Department of Neuroscience, Faculty of Health and Medical Sciences, University of Copenhagen, Copenhagen, Denmark

[2]Department of Computer Science, University of Copenhagen, Copenhagen, Denmark

[3]Department of Neuroscience, Karolinska Institutet, Stockholm, Sweden

SUPPLEMENTARY MATERIAL

- **Supplementary Text: Extended Results** 2
- **Supplementary Text: Extended Discussion** 8
- **Supplementary Text References** 16
- **Supplementary Table 1: Extended Statistical Report** 21
  Statistical report for all Main Figures (1 to 6) and Extended Data Figures (1 to 10)
- **Supplementary Video Descriptions** 30
  Descriptions for Supplementary Videos (1 to 4)

Supplementary Text: Extended Results

Apnea and a reduction in heart rate accompany the motor arrest

The PPN has previously been shown to have a role in autonomic regulation, for example, glutamate microinjections induce diverse changes in respiration and the cardiovascular system that exhibit functional topography [1, 2]. Although these effects are believed to be mainly mediated by cholinergic neurons, the employed techniques, and the heterogeneous neurochemical milieu of the PPN did not allow for unequivocally determining the neurons responsible for respiratory and cardiovascular changes. Therefore, the neurochemical substrate of such changes remains unclear, and it is possible that glutamatergic cells are partly responsible.

To understand the effect sizes of the Chx10-PPN activation (blue light, 1 s, 40 Hz) on respiratory and heart rate at a subject level (beyond the mean rates after pooling all trials from all mice **Fig. 4a, b**), we looked at the maximum drop in respiratory and heart rate by computing the difference between the baseline average rate (5 s before light onset, set as 100 %) and the lowest rate found within 1.5 seconds from light onset (i.e., the light on period plus 500 ms after light offset to account for the delayed dynamics in heart changes) for each mouse (continuous smooth mean rate of all the trials of individual mice, N = 10 mice), where no change from baseline would yield 0 % change and apnea would correspond to -100 % (**Fig. 4c**). The average maximum change from baseline in respiratory rate was -93.81 ± 8.51 % (mean ± SD; min, max: -81.62, -100 %) (**Fig. 4c**, cyan) while the heart rate was -18.48 ± 6.19 % (mean ± SD; min, max: -10.45, -28.24 %) (**Fig. 4c**, orange), corresponding to a significant drop in both rates compared to the baseline rates of each mouse.

We next investigated the effect of a longer stimulation time (blue light, 40 Hz, 3 s; N = 6 mice, n = 83 trials) (**Fig. 4d**) corresponding to the stimulus length used in the cylindrical arena (**Fig. 2**). Chx10-PPN activation led again to apnea in most cases, although in some trials the apnea only lasted through the first half of the stimulation, after which slow breathing emerged (**Fig. 4d**, top-left, raster). Considering all trials together, the average breathing rate dropped from a baseline average of 4.64 Hz to 0.51 Hz in the first half of the stimulation (between 0.1 and 1.5 s during light on), which slightly increased to 2.17 Hz during the second half (**Fig. 4d**, bottom-left, PSTH). While many trials only showed apnea in the first half of the stimulation, the drop in respiratory rate during the entire light on period remained significantly reduced as compared to baseline (**Extended Data Fig. 6d**, 3 s, left, blue light; Tukey's multiple comparisons test, adjusted p values: before vs. light on, $p = 0.0003$; light on vs. after, $p < 0.0001$; before vs. after, $p = 0.0147$). The post-stimulus rebound in breathing rate was even more prominent than with shorter stimulus duration. The rate increased to an

average of 7.24 Hz (between 0.1 and 1.1 s after light off) before returning to baseline, causing a significant difference between the average respiratory rate before and after light (see above, before vs. after) (**Fig. 4d, bottom-left, PSTH; Extended Data Fig. 6a, d**). This longer stimulation protocol led to a maximum change in respiratory rate of -96.24 ± 4.48 % (mean ± SD; min, max: -90.18, -100 %) (**Fig. 4e**, cyan).

The 3 s stimulation unmasked an even clearer bradycardic effect upon Chx10-PPN activation (**Fig. 4d**, top-right, raster), with a drop in heart rate from a baseline average of 621 bpm to an average of 536 bpm during light on (**Fig. 4d**, bottom-right, PSTH; **Extended Data Fig. 6d**, 3 s, right, blue light; Tukey's multiple comparisons test, adjusted *p* values: before vs. light on, $p < 0.0001$; light on vs. after, $p < 0.0040$; before vs. after, ns). These data suggest that the heart rate reduction had not plateaued with the 1 s stimulus but did so with the 3 s stimulation (**Fig. 4d**, bottom-right, PSTH; average of 575 bpm during the first 1 s of stimulation, down to 517 bpm during the last 2 s). This slightly larger reduction in heart rate obtained with the 3 s-long activation of Chx10-PPN neurons was consistently observed at an individual level with a maximum change from baseline of -23.05 ± 2.89 % (mean ± SD; min, max: -20.64, -27.01 %) (**Fig. 4e**, orange), a drop that in all mice was larger than the variability observed during baseline (**Extended Data Fig. 6a**). The dynamics observed in respiratory and heart rate changes were consistent across mice for both 1 and 3 seconds of stimulation (**Extended Data Fig. 6a, d**). In contrast, stimulation with yellow light (593 nm, 40 Hz, 1 or 3 s) as a control for ChR2 activation had no effect on respiratory or heart rate (**Extended Data Fig. 6b-d**), while blue light consistently decreased both (**Fig. 4b-e, Extended Data Fig. 6a, d**). Interestingly, despite the strong motor and respiratory modulation observed upon Chx10-PPN neuron activation, mice did not perceive it as aversive as tested in a conditional place aversion test (**Extended Data Fig. 6e and f**).

To explore the boundaries of the motor and autonomic effect of Chx10-PPN activation, we delivered prolonged blue light trains of up to 20 seconds with the same parameters used in all previous stimulations (10 ms pulse width, 40 Hz) (**Extended Data Fig. 7a**). The motor arrest was sustained throughout the entire length of the stimulation. However, the respiratory centers escaped the arrest and after up to ~3.5 seconds of apnea, a breathing pattern resumed with a slow regular rhythm ensuring animal survival through the prolonged body motor arrest. Once the brake that Chx10-PPN activation exerts was lifted, all activity recovered immediately (**Extended Data Fig. 7a**).

A well-known link exists between locomotor activity and changes in respiratory rhythm [3, 4]. A possible explanation for the observed respiratory changes could therefore be that they are

secondary to the motor arrest evoked from Chx10-PPN activation. To examine this issue in more detail, we used intercostal EMG recordings in mice under anesthesia (ketamine/xylazine) to monitor respiratory changes decoupled from movement arrest. In this context where mice expressed a slow anesthesia-induced respiratory rate (average baseline rate 2.86 Hz; n = 270 trials, from N = 10 mice), the activation of Chx10-PPN neurons could still induce apnea (**Extended Data Fig. 7b-c**, n = 50 trials, from N = 2 mice), but the more common response was a reduction of the respiratory rate (40.17 % reduction; from an average of 2.93 Hz before light, to an average of 1.75 Hz during stimulation; n = 220 trials from N = 8 mice) (**Extended Data Fig. 7b-c**). Therefore, these results indicate that changes in respiratory frequency are not a consequence of the motor arrest but occur due to a direct effect on the respiratory rhythm. We found similar evidence in awake mice, where Chx10-PPN activation randomly delivered when the animal was already immobile also caused apnea (data not shown, see peer-review file). Under anesthesia, Chx10-PPN activation also caused a mild slowing of the heart rate although much less evident than in awake mice (from a baseline rate of 252 bpm to 235 bpm during stimulation; n = 237 trials, N = 8 mice) (data not shown).

The direct effect on respiration was further highlighted when we delivered short trains of blue light stimuli (250 ms long, 40 Hz, 10 ms pulse width) randomly throughout the different phases of the respiratory period (n = 293 trials from N = 8 mice). The short trains of stimuli influenced the respiratory rhythm in a phase-dependent manner (**Extended Data Fig. 7d-e**). When the stimulus train overlapped with the onset of the inspiratory phase of respiration (189/293 trials), the stimulation either prevented (133/189) or shortened (28/189) the inspiratory burst, leading in both cases to a phase advance of the following inspiratory burst and a resetting of the respiratory rhythm (**Extended Data Fig. 7d**, middle trace). On the remaining trials (28/189) the stimulation shortened the burst without changing the respiratory rhythm. In contrast, if the stimulation exclusively overlapped with the expiratory phase (53/293), it delayed the coming burst without leading to a resetting of the rhythm (**Extended Data Fig. 7d**, bottom trace). Lastly, if the stimulation was delivered during the inspiratory phase but once the burst had already started (51/293), the ongoing burst was shortened but there was no change in the respiratory rhythm (**Extended Data Fig. 7d**, top trace). These results suggest that Chx10-PPN activation acts directly on the inspiratory rhythm-generating circuits [5].

Collectively, these results add additional features to the phenotypic fingerprint of the Chx10-PPN evoked motor arrest: during the “pause” period animals show apnea and mild bradycardia, and during the “play” period respiration overshoots but quickly returns to pre-stimulus values as does

the heart rate. The global motor arrest therefore includes a direct effect over respiration. For longer periods of stimulation, the respiratory centers are able to escape the arrest and set to a slower rhythm that allows the animal to survive. The effect on the heart rhythm, which displays slower dynamics, may at least partly be secondary to the arrest of motor and respiratory activity.

Importantly, we found that mice spontaneously perform arrest bouts with the same motor and autonomic features in the absence of experimental neuronal manipulations (**Fig. 4a,** black arrowheads). To further explore this phenomenon, we performed whole-body plethysmography recordings in baseline conditions using the same experimental setup as before (**Fig. 4f,** left). We then searched for periods of apnea and asked if they were accompanied by simultaneous motor arrest. To classify absence of breathing as apnea, we only considered events that were at least 500 ms long, which was the 99th percentile for all inter-PIF-intervals (PIF = peak inspiratory flow) recorded in baseline conditions. For each apnea event, we computed the percentage of time that the animals' activity was below the inactivity threshold (similar approach as in **Fig. 2f**) and labelled the apnea event as a true apneic motor arrest only if the animal was inactive for at least 80 % of the time of the apnea duration (see examples of true apneic arrest events in **Fig. 4f,** right).

Within the 18-minutes recorded from all 15 mice for which baseline measurements were taken, we identified a total of 1088 events as true apneic motor arrest (longer than 500 ms). However, they were heterogeneously distributed among mice. Although on average each mouse performed such apneic arrest bouts 72.53 ± 42.91 times (mean ± SD) within a 18 minute period, some mice showed as little as 13 events while others were above 100 (amount of apneic motor arrest events in 18 minutes: median = 60 events; 25th and 75th percentiles = 38 and 96 events; min, max = 13, 164 events). We found that the mean duration of naturally occurring apneic arrest events was 693.93 ± 47.35 ms (mean ± SD) (note that we restricted the minimum duration to be 500 ms). From the longest apneic arrest event recorded for each mouse, the average maximum duration was 1.38 ± 0.31 s (mean ± SD), with the longest of all naturally occurring events detected reaching 1.91 seconds. Therefore, these searching criteria resulted in a highly variable dataset underscoring the diversity in mobility and autonomic patterns that mice show in baseline conditions and demonstrates the expression of non-homogeneous behavioral states despite all mice having the same housing and recording conditions.

To obtain bouts of simultaneous apnea and motor arrest that were comparable to the optogenetically-evoked ones, we selected periods that were close to the length of these (apnea > 800 ms), and found a total of 260 events from 14 mice, as one mouse did not perform any

event longer than 800 ms (amount of long apneic motor arrest events in 18 minutes: median = 20.5 events; min, max = 4, 36 events). After the sorting, we found that the long apnea periods were almost always correlated with movement arrest: 92.53 % of all apnea events longer than 800 ms fulfilled the criteria of simultaneous inactivity and were considered as true apneic motor arrest events (260/281). Notably, and despite the heart rate not being used as a search criterion, the naturally occurring long apneic motor arrest events were also reliably accompanied by slowing of the heart rate similar to that evoked from Chx10-PPN stimulation (**Fig. 4g**, right). The average maximum change in heart rate from baseline during naturally occurring long apneic arrest events was -17.82 ± 6.86 % (mean ± SD; min, max: -5.21, -28.74 %), compared to -18.48 ± 6.19 % (mean ± SD; min, max: -10.45, -28.24 %) upon 1 second activation of Chx10-PPN neurons (**Fig. 4g**, right) (two-tailed unpaired *t*-test, Chx10-PPN vs. natural, difference between means = -0.65 %, CI = -6.23 to 4.93 %, $p = 0.8102$, ns). These results confirm that the behavioral pattern observed upon Chx10-PPN neuron activation also exists under natural conditions possibly elicited by natural triggers such as salient environmental cues.

vlPAG-evoked freezing is different from Chx10-PPN arrest

Limb coordination during Chx10-vlPAG stimulation

To further assess limb coordination, we performed the same limb coordination analysis as for Chx10-PPN stimulation. We find that the regular alternation observed before stimulation becomes a flat line as observed for the Chx10-PPN stimulation, but because the hindlimbs most often were on the ground and aligned perpendicular to the body axis, the flat lines during arrest were less spread than during Chx10-PPN activation and more concentrated around 0 degrees (**Extended Data Figure 8e**). The difference in limb coordination during arrest between Chx10-vlPAG and Chx10-PPN neuron activation was also apparent when plotting the hindlimb coordination as the line that connects the left and right paws during arrest. While Chx10-PPN activation trials show lines covering a wide range of angles, Chx10-vlPAG trials are concentrated perpendicular to the body axis (**Extended Data Figure 8f**). Given the long latencies to resume locomotion after light off, we did not quantify continuity as for Chx10-PPN. However, visual evaluation of behavior shows that mice re-start a new step cycle from the resting position adopted during the movement arrest, or a behavior different from locomotion, e.g. exploratory sniffing (see **Supp. Video 4**).

Chx10-PPN neurons project widely through the neuroaxis

Chx10-PPN neurons have widespread descending and ascending projections. The descending projections of Chx10-PPN neurons target all major motor-related pontine nuclei including the pontine reticular nucleus oral (PnO), caudal (PnC), and ventral (PnV) parts, and the dorsomedial tegmental nucleus (DMTg), that are heavily innervated by Chx10-PPN neurons (**Fig. 6c, d**). Similarly, all major motor structures in the medulla are targeted by Chx10-PPN neurons. The medullary areas with the highest bouton density are the gigantocellular reticular nucleus (Gi), including the alpha (GiA) and ventral (GiV) parts, the lateral paragigantocellular nucleus (LPGi), and to a lesser extent, the ventral medullary reticular nucleus (MdV) and dorsal paragigantocellular nucleus (DPGi). All of these structures contain reticulospinal neurons that influence spinal motor circuits [6, 7]. Notably, respiratory motor centers including the Bötzinger (Bo) complex, the preBötzinger (PreBo) complex, the hypoglossal nucleus (N12), and the rostral ventral respiratory group (RVRG) [5] are targeted by Chx10-PPN neurons. Moreover, we found that the rostroventrolateral (RVL) and caudoventrolateral (CVL) reticular nuclei, which mediate blood pressure modulation [8, 9] are also innervated, as is the cardioinhibitory ambiguus nucleus (Amb). The prepositus nucleus (Pr), involved in vertical and horizontal gaze control [10] is also innervated by Chx10-PPN neurons as is the cap of Kooy (IOK) in the central inferior olive, related with the control of eye movements [11] (**Fig. 6d and Extended Data Fig. 10**). Finally, the caudal raphe nuclei: the raphe magnus (RMg), raphe obscurus (Rob), and raphe pallidus (RPa), which provide modulatory inputs to motor and sensory circuits in the spinal cord [12, 13] are also densely innervated.

Although the bulk of the projections are descending, Chx10-PPN neurons, similar to other neuron types within the PPN [14, 15], also have ascending projections (**Extended Data Fig. 10**). These ascending fibers target almost exclusively the ipsilateral side. In the diencephalon, the ascending projections target, among other structures, the parafascicular (PF) nucleus, thought to be involved in attention and behavioral flexibility [16-18]; the retroparafascicular nucleus (RPF), which receives projections from the preBötzinger complex and may be involved in coordinating respiration with other behaviors [19], and the magnocellular nucleus of the posterior commissure (MCPC), related with saccadic and gaze movement control [20] (**Extended Data Fig. 10**). In the midbrain and rostral pons, structures including the mesencephalic reticular formation (mRt), involved among other functions in gaze control [21], and the laterodorsal tegmental nucleus (LDTg), the locus coeruleus (LC), and the dorsal raphe nuclei (DR, DRL, PDR), which are all part of the ascending reticular activating system and are implicated in arousal, attention, and vigilance [22-24], receive Chx10-PPN innervation (**Fig. 6d, Ext. Data Fig. 10**).

Supplementary Text: Extended Discussion

The episodic nature of movement implies that its execution needs to be arrested or momentarily interrupted at a certain point. The underlying triggers of movement arrest are diverse, from volitional to defensive.

The present study has uncovered a brainstem command evoked upon activation of $Chx10^+$ neurons in the PPN that mediates a unique type of global motor arrest different from other forms of motor interruption. This global motor arrest could be recruited in response to salient environmental stimuli, since during baseline conditions and in the absence of threatening stimuli we observed that mice naturally perform bouts of motor arrest accompanied by apnea and heart rate changes similar to those evoked by Chx10-PPN neuron stimulation. In addition, our study also highlights a dual and opposing role of glutamatergic PPN neurons in motor control that seems to be linked to their location in the caudal (e.g. Masini and Kiehn 2022) and rostral portions (this study) of the PPN.

A unique motor arrest

The global motor arrest evoked from Chx10-PPN neurons involves virtually all motor behaviors including respiration, and is also associated with changes in heart rate. The distinctive feature of the Chx10-PPN evoked motor arrest is a "pause-and-play" pattern. During the "pause", the nervous system appears to keep the execution of movement on hold in a sort of memory function. The pause is followed by a "play", where movement execution resumes from the exact position and expected course from when it was halted. Both the "pause" from light onset, and the "play" from light offset, occur with a short latency. This characteristic pattern differentiates the Chx10-PPN induced motor arrest form other described motor arrest types as discussed below.

Activation of glutamatergic "stop neurons" in the brainstem of fish and mice halts locomotion [25, 26]. In the mouse this leads to a canonical stop where the animal finalizes the step cycle and adopts a typical posture where the hindlimb and forelimb are brought into a perpendicular position that allows all limbs to be on the ground simultaneously [25]. After termination of the stop, the limbs regain their walking phases from that position. Movement can also be arrested through basal ganglia activity. For example, bilateral optogenetic stimulation of subthalamic nucleus (STN) neurons inhibits ongoing locomotion [27, 28] recruited either by internal basal ganglia activity or for example by premotor cortico-subthalamic neurons recruited during visually-guided locomotion to execute a learned motor stop [29]. These types of arrest of motor behaviors have a slow onset and a stereotypical motor outcome. Similarly, the amygdala-driven and exploration-related stop that is

thought to act by inhibiting the locomotor promoting areas within the mesencephalic locomotor region —caudal Vglut2-PPN and Vglut2-CnF neurons— also displays slow arrest of behavior with long onset and offset latencies [30].

Lastly, a well-known form of motor arrest is the vlPAG-driven fear-related defensive freezing, which can be expressed as an innate behavior following threat detection or as a fear conditioned response [31-33]. Bilateral optogenetic stimulation of virally infected glutamatergic (Vglut2^{+}) neurons in the vlPAG evokes defensive freezing in mice [34]. More recently, preliminary data from our group [35] later reproduced by Vaaga et al., 2020 [36] showed that unilateral optogenetic stimulation of the Chx10^{+} subpopulation in vlPAG in transgenic mice also evokes freezing. These results have been extended in a newly published study with bilateral viral injections in *Chx10^{Cre}* mice [37].

For direct comparison with the Chx10-PPN evoked motor arrest, we here focused on characterizing the Chx10-vlPAG evoked motor response. We found that the dynamics of the motor phenotype observed during the Chx10-vlPAG evoked freezing were different when compared to the Chx10-PPN evoked global motor arrest. While both had a similar latency to onset, the freezing response evoked from the Chx10-vlPAG had a much slower recovery than the Chx10-PPN induced motor arrest, and mice remained immobile without immediately resuming the behavior they were previously engaged on. Moreover, upon Chx10-vlPAG neuron stimulation mice typically adopted a stereotyped freezing posture with both hindlimbs on the ground, as opposed to the variable postural positions observed upon Chx10-PPN neuron activation. The defensive freezing, as opposed to the Chx10-PPN evoked motor arrest, is therefore not characterized by a “pause-and-play” pattern. In addition to the motor output, the respiratory modulation is also different: Chx10-vlPAG stimulation leads to an overall reduction in respiratory rate, typically adopting a steady slow breathing rhythm after a short initial period of tachypnea, while Chx10-PPN stimulation typically leads to full apnea in the awake animal. We also find that although both Chx10-PPN and Chx10-vlPAG neurons evoke slowing of the heart rate, the bradycardic effect evoked upon Chx10-PPN activation appeared slightly larger. Importantly, the slowing of the heart rate we find here upon Chx10-vlPAG stimulation has also been described by Signoret-Genest and colleagues (2023) in a recently published study which shows that the phenotypic vlPAG-evoked freezing is consistently mediated through the activation of Chx10-vlPAG neurons rather than the Vglut2-vlPAG neuron population at large [37]. These findings confirm that overall the response to Chx10-vlPAG neuron stimulation is similar whether evoked in transgenic mice or after viral infection. Regardless of the targeting approach, collectively these findings

highlight that the light-evoked phenotype is different between Chx10-vlPAG vs. Chx10-PPN neuron activation.

Apart from leading to phenotypically different motor and respiratory outputs, we also find additional evidence that makes it unlikely that the defensive freezing and the Chx10-PPN evoked motor arrest are related or partially mediated by the same neuronal pathways. The neuronal pathway that mediates glutamatergic vlPAG-driven freezing responses was described by Tovote et al., 2016 [34] to be: central nucleus of the amygdala (CEA) > vlPAG > magnocellular reticular nucleus (Mc) > spinal cord. The authors also demonstrated that direct activation of Mc-projecting glutamatergic vlPAG neurons evoked freezing [34]. Vaaga et al., 2020 [36] corroborated these findings by showing that Chx10-vlPAG neurons also project directly to Mc. Therefore, glutamatergic vlPAG-evoked freezing, including the Chx10-vlPAG-evoked freezing, does not require the PPN to be executed. On the other hand, the fact that we find projections from Chx10-PPN neurons to neurons in the vlPAG (**Extended data Fig. 10**) opens for the possibility that the Chx10-PPN evoked motor arrest may be partly mediated through the vlPAG. However, given that Chx10-PPN neuron projections largely target brainstem areas where both neurons bridging with the executive spinal cord circuits, and circuits controlling respiration and heart-rate reside (see below), such descending connections seem sufficient to explain the observed phenotype. Nevertheless, this does not exclude the possibility that the projection from Chx10-PPN neurons to the vlPAG could in parallel engage vlPAG neurons, either $Chx10^{+}$-vlPAG or other vlPAG neuronal subtypes. We have not tested this possibility directly. Although both Chx10-vlPAG and Chx10-PPN neurons could converge at the level of the medullary reticular formation where their projections overlap, with the arguments laid out above we find it compelling to think that the global motor arrest evoked from Chx10-PPN neuron activation is unrelated to the motor pathways mediating defensive freezing because of their phenotypic difference.

In sum, the global motor arrest evoked by Chx10-PPN activation is different from other previously described forms of motor arrest by having a pause and play pattern. In addition, our study underscores the relevance of having rich behavioral descriptions, including the characterization of multiple physiological responses like respiration and cardiac features, to tell apart two behaviors that may appear similar on surface, but are ultimately different.

Mechanisms of action

The neuronal mechanisms for the implementation of the characteristic triad of Chx10-PPN-induced output actions, i.e., the global motor arrest, apnea or severe reduction in respiratory rate, and heart

modulation, have not been directly addressed in this study. However, based on the divergent descending projection pattern from Chx10-PPN neurons that we observed, we propose that the triad involves parallel actions at the level of the brainstem followed by multiple actions at the executive motor circuits in the spinal cord.

The mechanism for the respiratory modulation is perhaps what lends itself to the most straight forward explanation, given the direct projections from Chx10-PPN neurons to respiratory centers in the medullary reticular formation. The Chx10-PPN induced apnea shares striking similarities with the apnea induced by optogenetic stimulation of inhibitory glycinergic neurons in the preBötzinger complex [38]. The authors showed that short-lasting activation of the glycinergic neurons in the preBötzinger complex can suppress individual inspiratory bursts and reset the inspiratory rhythm, similar to what we observed from short lasting stimulation of Chx10-PPN neurons (**Extended Data Fig. 7d and e**). These similarities in action together with the demonstrated projection to the preBötzinger complex from Chx10-PPN neurons (**Fig. 6, Extended Data Fig. 10**) suggest that Chx10-PPN neurons could inhibit respiration by activating inhibitory circuits in the preBötzinger complex. The direct effect of Chx10-PPN neurons on respiratory rhythmogenesis is further supported by the fact that the respiratory modulation was also observed in anesthetized mice and not only in awake freely moving subjects, showing that the evoked change in respiration is decoupled from movement and, therefore, is not a phenomenon secondary to the movement arrest itself (**Extended Data Fig. 7b-e**). Chx10-PPN neurons also project to other respiratory areas including the Bötzinger complex, the hypoglossal nucleus (12N), and premotor areas in the rostral ventral respiratory group (RVRG) [5]. Therefore, there are several entry points to the respiratory regulation centers that could explain the respiratory modulation by Chx10-PPN, and their nature could be addressed in future experiments.

The mechanism for heart rate modulation can be explained by two non-mutually-exclusive scenarios. Chx10-PPN neurons may either directly evoke a bradycardic effect through a feedforward activation of brainstem nuclei controlling cardiac functions, or the heart rate reduction could be a secondary phenomenon implemented through indirect feedback regulation triggered by primary changes in respiration and/or movement. A direct feedforward effect could for example be mediated through activation of neurons in the cardioinhibitory ambiguus nucleus [39-41], which is innervated by Chx10-PPN neurons. Inhibition of the sympathetic tone, e.g., via neurons in the ventrolateral reticular nuclei [42], may also contribute to a reduction of the heart rate. In addition to the direct effects, the motor arrest and apnea may lead to a delayed reduction in the heart rate via

regulatory feedback mechanisms [40]. The slower dynamics observed in heart rate changes compared to the motor and respiratory output suggest that a secondary effect may also be involved.

The mechanism(s) for implementing the global motor arrest is/are the most difficult and at the same time probably the most interesting to explain. The global nature of the motor arrest implies simultaneous control over multiple axial and limb muscles on both sides of the body through an active process that is not a mere absence of movement/motor-drive, generalized atonia, or a massive co-contraction, but rather a carefully orchestrated motor response. Since Chx10-PPN neurons do not project directly to the spinal cord (**Extended Data Fig. 9b**), the motor arrest must be mediated by one or most likely the coordinated actions of several descending motor-related brainstem nuclei that receive input from the Chx10-PPN neuron population. Interestingly, Chx10-PPN projections follow a predominantly ipsilateral pattern and do not target the contralateral PPN. However, the symmetric nature of the motor arrest even when Chx10-PPN neurons are unilaterally stimulated suggests that the select group of brainstem nuclei that receive bilateral input from Chx10-PPN neurons are the prime candidate for mediating the global motor arrest in a symmetric fashion. The nuclei receiving bilateral projections include the PnC, PnV, Gi, GiA, GiV, and LPGi. All of these areas contain reticulospinal motor-related neurons that project broadly to the ventral spinal cord where motor circuits are located, and mediate predominantly excitatory effects although inhibitory descending pathways are also present [6, 43, 44].

The two features of the motor arrest —the fact that the arrest command doesn't lead to the adoption of a stereotypic posture, and the "pause-and-play" pattern— exclude the possibility that the descending commands act by directly activating motor neurons. Given that the conduction velocity of reticulospinal neurons and motor neurons in adult mice is in the range of 50 m/s [45] and 35-40 m/s [46], respectively, and given that the latency for motor arrest was on average 110 ms (ranging from 40 to 220 ms) it is unlikely that the arrest is mediated by a direct action on motor neurons. Instead, this action must be executed through distributed interneuron motor circuits in the spinal cord. Although we observed that these two features are also present when arresting grooming or rearing, a rhythmic behavior like locomotion provides a more comprehensive framework to explain the underlying mechanisms. Our data shows that during locomotion the flexor-extensor alternation is blocked throughout the "pause" keeping the coordination pattern on hold by maintaining tonic activity in motor neurons that are in the active phase, while preventing the expression of rhythmic activity in motor neurons that are inactive. This "pause" pattern could be explained by an excitatory action from the brainstem on the rhythm generating circuits in the

spinal cord where either flexor or extensor rhythm generating circuits are locked in their active phase causing a constant drive to the corresponding flexor or extensor motor nuclei, while they simultaneously inhibit the opposing rhythm generating circuits leading to a decreased drive to their corresponding motor nuclei. The rhythm generating circuit (flexor or extensor) that is active when the Chx10-PPN command reaches the spinal cord "wins" the game and is locked in its pause position throughout the command. A corresponding example of such a mechanism is seen when the flexor rhythm generation in the limb is arrested by tonically activating extensor rhythm generation via proprioceptive afferent activity in the hindlimb [47, 48]. The possibility for instantaneous regaining of cyclic activity seen in those studies when the excitation ceased would also explain the apparent "memory function" that we observe, which in this case would be an inbuilt feature of the spinal circuits. While still speculative, future experiments, using targeted recordings from rhythm generating and other spinal neurons [49-51], should be able to test these hypotheses in a more direct way.

Although not directly addressed in this work, in addition to the reticulospinal activation for postural and limb control, Chx10-PPN neurons also broadly innervate descending serotonergic pathways from the caudal raphe nuclei (raphe pallidus and obscurus) that modulate spinal motor circuits [13, 52]. The integration and significance of these descending pathways for the expression of the Chx10-PPN induced behavior may be clarified in future studies.

Expression of the global motor arrest under natural conditions

An intriguing aspect of the arrest behavior described in this work revolves around its possible function when expressed under natural conditions. Although we do not provide a causal link, we found that the same combination of motor arrest and heart rate changes as seen upon Chx10-PPN neuron activation can be observed in baseline conditions in the absence of experimental manipulations. Moreover, we find that the amount of short arrest bouts in the open field is reduced in most mice after the ablation of Chx10-PPN neurons. A confounding factor in the ablation experiment is that movement arrest in the open field might be triggered by neuronal circuits other than Chx10-PPN neurons (e.g. Bouvier et al. 2015; Botta et al. 2020) [25, 30]. So even if we had managed to obtain a complete ablation of Chx10-PPN neurons – which will be difficult to achieve due to the elongated shape of the nucleus – it is not expected that the arrest events would fully disappear. However, the fact that there is a reduction, as opposed to the increase observed in control mice, indicates that part of the naturally occurring motor arrest events are mediated by the Chx10-PPN neurons.

The naturally occurring arrest events that are linked to the Chx10-PPN neurons may happen e.g. during exploration, and we hypothesize that these natural brief arrest bouts may be triggered by salient but non-threatening sensory inputs. The temporary behavioral interruption might be accompanied by or lead to an increase in attention. In the present study we did not identify the input structures to Chx10-PPN neurons or a sensory stimulation paradigm to reliably trigger the global motor arrest. However, it is known that the PPN receives and integrates polymodal sensory input [53, 54]. Moreover, it has been suggested that the PPN plays a role in regulating attention based on both lesion studies and its ascending projection pattern [15, 54]. Specifically, the PPN is thought to play a role in global behavioral state transitions, mainly through ascending projections that target thalamic and dopaminergic neurons, which apart from the processing of sensory inputs within the PPN it also implies assessing their motivational value and having sensitivity to unexpected events [15]. In accordance with this, we also find that Chx10-PPN neurons have ascending projections to areas that have been ascribed a role in regulating the processing of unexpected and behaviorally relevant sensory stimuli, attention, and arousal, such as the thalamic parafascicular nucleus, the laterodorsal tegmental nucleus, the locus coeruleus, and the dorsal raphe nucleus [16, 18, 22, 24] (**Extended Data Figure 10**). Therefore, the global motor arrest triggered by Chx10-PPN neurons could be concomitant to an attention-related brain-state.

The attentional shift when reacting to novel environmental cues might be facilitated by the global motor arrest, but it could also be either the trigger or a consequence of it. Regardless of chronology, we hypothesize that the arrest evoked from the activation of Chx10-PPN neurons could be embedded within an attention-related cognitive state. Such a role would highlight the integrative role of the PPN as-a-whole in driving both motor and cognitive aspects for a coherent behavioral response.

The dual role of glutamatergic PPN neurons in motor control

Our study demonstrates that a subpopulation of glutamatergic neurons in the PPN has a movement-opposing effect. Therefore, the results presented in this work together with previous evidence lead to a model where the PPN has a dual opposing role in motor control depending on the subpopulation of glutamatergic cells involved: activation of glutamatergic neurons predominantly located in the caudal part of the nucleus promotes locomotion [55-58], while the specific activation of glutamatergic Chx10-PPN neurons, which are enriched in the rostral PPN, evokes global motor arrest. Although our viral infection and probe implantation approaches favored

rostral Chx10-PPN neuron activation, we do not exclude that more caudally localized Chx10-PPN neurons may also contribute to the phenotype.

The observed behavioral effect upon Chx10-PPN activation resembles the behavior reported in rats upon activation of excitatory neurons in the rostral PPN [59]. The authors targeted PPN neurons using a non-cre-dependent viral approach to express ChR2 under the CaMKIIa promoter, which preferentially targets excitatory cells in the MLR [55]. In most cases, stimulation elicited a locomotor response in agreement with previous reports [55-58]. However, in a small subset of rats coinciding with rostral fiber placement, the authors observed that stimulation interrupted locomotion although there was no extensive description of the motor phenotype. It is likely that in those cases the authors mainly targeted Chx10$^+$ glutamatergic neurons due to their rostral bias within the PPN. In contrast to those mentioned above, some studies have been unable to demonstrate locomotor initiation by glutamatergic PPN neuron activation [60, 61]. Instead, they showed that stimulation of these neurons in non-moving animals elicited phasic [60] or tonic muscle activity [61], and also reported arrest of movement during ongoing locomotion [60, 61]. The observed mixed effects could be explained by the fact that broad activation of Vglut2$^+$ neurons in PPN may include both Chx10$^-$ and Chx10$^+$ neurons, which by having opposing roles may lead to mixed actions depending on the subpopulation that is predominantly activated during stimulation. A definitive evidence for this proposal would require the stimulation of Chx10-negative Vglut2-positive PPN neurons locally (caudally or rostrally) or broadly by using an intersectional approach, which we have not done here. However, the present study shows that the arrest is solely linked to the Chx10-PPN neurons, which are glutamatergic and enriched in the rostral part and, therefore, provides a direct explanation for the controversy in the field regarding the diverse contribution of glutamatergic PPN neurons to movement control.

Given the implication of the PPN in the pathogenesis of Parkinson's disease (PD), our findings could potentially have translational value. The PPN has been used as a target in deep brain stimulation (DBS) approaches to ameliorate PD symptoms with variable outcomes [62-66]. Based on recent findings from our group [57, 58] and others [59, 65], in combination with the insights from the present work, it is likely that a successful approach for DBS targeted to the PPN to alleviate PD locomotor dysfunctions should avoid the rostral part of the nucleus to prevent the engagement of the Chx10$^+$ population. Instead, it should aim to engage the caudal glutamatergic neurons (mostly Vglut2$^+$/Chx10$^-$), which comprise the majority of glutamatergic neurons in the PPN, have a locomotor-promoting role [55-59], and have already been shown to ameliorate gait deficits in parkinsonian animal models [58, 65].

Supplementary Text References

1. Saponjic, J., Radulovacki, M. & Carley, D.W. Respiratory pattern modulation by the pedunculopontine tegmental nucleus. *Respiratory Physiology and Neurobiology* **138**, 223-237 (2003).

2. Topchiy, I., Waxman, J., Radulovacki, M. & Carley, D.W. Functional topography of respiratory, cardiovascular and pontine-wave responses to glutamate microstimulation of the pedunculopontine tegmentum of the rat. *Respiratory Physiology and Neurobiology* **173**, 64-70 (2010).

3. Bramble, D.M. & Carrier, D.R. Running and breathing in mammals. *Science (New York, N.Y.)* **219**, 251-256 (1983).

4. Gariépy, J.F., Missaghi, K. & Dubuc, R. The interactions between locomotion and respiration. *Progress in Brain Research* **187**, 173-188 (2010).

5. Del Negro, C.A., Funk, G.D. & Feldman, J.L. Breathing matters. *Nature Reviews Neuroscience* **19**, 351-367 (2018).

6. Brownstone, R.M. & Chopek, J.W. Reticulospinal Systems for Tuning Motor Commands. *Frontiers in Neural Circuits* **12** (2018).

7. Liang, H., Watson, C. & Paxinos, G. Terminations of reticulospinal fibers originating from the gigantocellular reticular formation in the mouse spinal cord. *Brain Structure and Function* **221**, 1623-1633 (2016).

8. Stocker, S.D., Steinbacher Jr, B.C., Balaban, C.D. & Yates, B.J. Connections of the caudal ventrolateral medullary reticular formation in the cat brainstem. *Experimental Brain Research* **116**, 270-282 (1997).

9. Sun, W. & Panneton, W.M. Defining projections from the caudal pressor area of the caudal ventrolateral medulla. *The Journal of Comparative Neurology* **482**, 273-293 (2005).

10. McCrea, R.A. & Horn, A.K.E. Nucleus prepositus. 205-230 (2006).

11. Llinás, R.R. The olivo-cerebellar system: a key to understanding the functional significance of intrinsic oscillatory brain properties. *Frontiers in Neural Circuits* **7** (2014).

12. Jacobs, B.L. & Fornal, C.A. 5-HT and motor control: a hypothesis. *Trends in Neurosciences* **16**, 346-352 (1993).

13. Schmidt, B.J. & Jordan, L.M. The role of serotonin in reflex modulation and locomotor rhythm production in the mammalian spinal cord. *Brain Research Bulletin* **53**, 689-710 (2000).

14. Gut, N.K. & Winn, P. The pedunculopontine tegmental nucleus—A functional hypothesis from the comparative literature. *Movement Disorders* **31**, 615-624 (2016).

15. Mena-Segovia, J. & Bolam, J.P. Rethinking the Pedunculopontine Nucleus: From Cellular Organization to Function. *Neuron* **94**, 7-18 (2017).

16. Bradfield, L.A., Hart, G. & Balleine, B.W. The role of the anterior, mediodorsal, and parafascicular thalamus in instrumental conditioning. *Frontiers in Systems Neuroscience* **7** (2013).

17. Saalmann, Y.B. Intralaminar and medial thalamic influence on cortical synchrony, information transmission and cognition. *Frontiers in Systems Neuroscience* **8** (2014).

18. Yamanaka, K.*, et al.* Roles of centromedian parafascicular nuclei of thalamus and cholinergic interneurons in the dorsal striatum in associative learning of environmental events. *Journal of Neural Transmission* **125**, 501-513 (2018).

19. Yang, C.F. & Feldman, J.L. Efferent projections of excitatory and inhibitory preBötzinger Complex neurons. *Journal of Comparative Neurology* **526**, 1389-1402 (2018).

20. Moschovakis, A., Scudder, C. & Highstein, S. The microscopic anatomy and physiology of the mammalian saccadic system. *Progress in Neurobiology* **50**, 133-254 (1996).

21. Wang, N., Perkins, E., Zhou, L., Warren, S. & May, P.J. Reticular Formation Connections Underlying Horizontal Gaze: The Central Mesencephalic Reticular Formation (cMRF) as a Conduit for the Collicular Saccade Signal. *Frontiers in Neuroanatomy* **11** (2017).

22. Jones, B.E. Arousal systems. *Frontiers in Bioscience* **8**, 1074-1074 (2003).

23. Samuels, E. & Szabadi, E. Functional Neuroanatomy of the Noradrenergic Locus Coeruleus: Its Roles in the Regulation of Arousal and Autonomic Function Part II: Physiological and Pharmacological Manipulations and Pathological Alterations of Locus Coeruleus Activity in Humans. *Current Neuropharmacology* **6**, 254-285 (2008).

24. Sara, Susan J. & Bouret, S. Orienting and Reorienting: The Locus Coeruleus Mediates Cognition through Arousal. *Neuron* **76**, 130-141 (2012).

25. Bouvier, J.*, et al.* Descending Command Neurons in the Brainstem that Halt Locomotion. *Cell* **163**, 1191-1203 (2015).

26. Juvin, L.*, et al.* A Specific Population of Reticulospinal Neurons Controls the Termination of Locomotion. *Cell Reports* **15**, 2377-2386 (2016).

27. Guillaumin, A., Serra, G.P., Georges, F. & Wallén-Mackenzie, Å. Experimental investigation into the role of the subthalamic nucleus (STN) in motor control using optogenetics in mice. *Brain Research* **1755**, 147226-147226 (2021).

28. Parolari, L., Schneeberger, M., Heintz, N. & Friedman, J.M. Functional analysis of distinct populations of subthalamic nucleus neurons on Parkinson's disease and OCD-like behaviors in mice. *Molecular Psychiatry* **26**, 7029-7046 (2021).

29. Adam, E.M., Johns, T. & Sur, M. Dynamic control of visually guided locomotion through corticosubthalamic projections. *Cell Reports* **40**, 111139 (2022).

30. Botta, P.*, et al.* An Amygdala Circuit Mediates Experience-Dependent Momentary Arrests during Exploration. *Cell* **183** (2020).

31. Carrive, P. The periaqueductal gray and defensive behavior: Functional representation and neuronal organization. *Behavioural Brain Research* **58**, 27-47 (1993).

32. Fanselow, M.S. Neural organization of the defensive behavior system responsible for fear. *Psychonomic Bulletin & Review* **1**, 429-438 (1994).

33. Carrive, P., Leung, P., Harris, J. & Paxinos, G. Conditioned fear to context is associated with increased Fos expression in the caudal ventrolateral region of the midbrain periaqueductal gray. *Neuroscience* **78**, 165-177 (1997).

34. Tovote, P.*, et al.* Midbrain circuits for defensive behaviour. *Nature 2016 534:7606* **534**, 206-212 (2016).

35. Leiras, R.*, et al.* (2017). An excitatory midbrain motor circuit for evoking freezing behavior. In *2017 Neuroscience Meeting Planner* (Vol. Program No. 232.08). Washington DC: Society for Neuroscience.

36. Vaaga, C.E., Brown, S.T. & Raman, I.M. Cerebellar modulation of synaptic input to freezing-related neurons in the periaqueductal gray. *eLife* **9** (2020).

37. Signoret-Genest, J.*, et al.* Integrated cardio-behavioral responses to threat define defensive states. *Nat Neurosci* (2023).

38. Sherman, D., Worrell, J.W., Cui, Y. & Feldman, J.L. Optogenetic perturbation of preBötzinger complex inhibitory neurons modulates respiratory pattern. *Nature Neuroscience* **18**, 408-414 (2015).

39. Nosaka, S., Yamamoto, T. & Yasunaga, K. Localization of vagal cardioinhibitory preganglionic neurons within rat brain stem. *The Journal of Comparative Neurology* **186**, 79-92 (1979).

40. Taylor, E.W., Jordan, D. & Coote, J.H. Central Control of the Cardiovascular and Respiratory Systems and Their Interactions in Vertebrates. *Physiological Reviews* **79**, 855-916 (1999).

41. Jordan, D. Vagal control of the heart: central serotonergic (5-HT) mechanisms. *Experimental Physiology* **90**, 175-181 (2005).

42. Sun, M. Central neural organization and control of sympathetic nervous system in mammals. *Progress in Neurobiology* **47**, 157-233 (1995).

43. Capelli, P., Pivetta, C., Soledad Esposito, M. & Arber, S. Locomotor speed control circuits in the caudal brainstem. *Nature* **551**, 373-377 (2017).

44. Takakusaki, K., Chiba, R., Nozu, T. & Okumura, T. Brainstem control of locomotion and muscle tone with special reference to the role of the mesopontine tegmentum and medullary reticulospinal systems. *Journal of Neural Transmission* **123**, 695-729 (2016).

45. Tanaka, H., Ono, K., Shibasaki, H., Isa, T. & Ikenaka, K. Conduction properties of identified neural pathways in the central nervous system of mice in vivo. *Neurosci Res* **49**, 113-122 (2004).

46. Walsh, M.E.*, et al.* Use of Nerve Conduction Velocity to Assess Peripheral Nerve Health in Aging Mice. *J Gerontol A Biol Sci Med Sci* **70**, 1312-1319 (2015).

47. Conway, B.A., Hultborn, H. & Kiehn, O. Proprioceptive input resets central locomotor rhythm in the spinal cat. *Experimental Brain Research* **68** (1987).

48. Duysens, J. & Pearson, K.G. Inhibition of flexor burst generation by loading ankle extensor muscles in walking cats. *Brain Research* **187**, 321-332 (1980).

49. Kiehn, O. Decoding the organization of spinal circuits that control locomotion. *Nature Reviews Neuroscience* **17**, 224-238 (2016).

50. Grillner, S. & El Manira, A. Current Principles of Motor Control, with Special Reference to Vertebrate Locomotion. *Physiological Reviews* **100**, 271-320 (2020).

51. Leiras, R., Cregg, J.M. & Kiehn, O. Brainstem Circuits for Locomotion. *Annual Review of Neuroscience* **45** (2022).

52. Jacobs, B.L., Martín-Cora, F.J. & Fornal, C.A. Activity of medullary serotonergic neurons in freely moving animals. *Brain Research Reviews* **40**, 45-52 (2002).

53. Winn, P. Experimental studies of pedunculopontine functions: Are they motor, sensory or integrative? *Parkinsonism & Related Disorders* **14**, S194-S198 (2008).

54. Winn, P. How best to consider the structure and function of the pedunculopontine tegmental nucleus: Evidence from animal studies. *Journal of the Neurological Sciences* **248**, 234-250 (2006).

55. Lee, A.M.*, et al.* Identification of a Brainstem Circuit Regulating Visual Cortical State in Parallel with Locomotion. *Neuron* **83**, 455-466 (2014).

56. Roseberry, T.K.*, et al.* Cell-Type-Specific Control of Brainstem Locomotor Circuits by Basal Ganglia. *Cell* **164**, 526-537 (2016).

57. Caggiano, V.*, et al.* Midbrain circuits that set locomotor speed and gait selection. *Nature* **553**, 455-460 (2018).

58. Masini, D. & Kiehn, O. Targeted activation of midbrain neurons restores locomotor function in mouse models of parkinsonism. *Nature Communications 2022 13:1* **13**, 1-23 (2022).

59. Carvalho, M.M.*, et al.* A Brainstem Locomotor Circuit Drives the Activity of Speed Cells in the Medial Entorhinal Cortex. *Cell Reports* **32**, 108123-108123 (2020).

60. Josset, N.*, et al.* Distinct Contributions of Mesencephalic Locomotor Region Nuclei to Locomotor Control in the Freely Behaving Mouse. *Current Biology* **28**, 884-901.e883 (2018).

61. Dautan, D.*, et al.* Modulation of motor behavior by the mesencephalic locomotor region. *Cell Reports* **36**, 109594-109594 (2021).

62. Mazzone, P.*, et al.* Implantation of human pedunculopontine nucleus: a safe and clinically relevant target in Parkinson's disease. *NeuroReport* **16**, 1877-1881 (2005).

63. Stefani, A.*, et al.* Bilateral deep brain stimulation of the pedunculopontine and subthalamic nuclei in severe Parkinson's disease. *Brain* **130**, 1596-1607 (2007).

64. Ferraye, M.U., *et al.* Effects of pedunculopontine nucleus area stimulation on gait disorders in Parkinson's disease. *Brain* **133**, 205-214 (2010).

65. Gut, N.K. & Winn, P. Deep Brain Stimulation of Different Pedunculopontine Targets in a Novel Rodent Model of Parkinsonism. *Journal of Neuroscience* **35**, 4792-4803 (2015).

66. Thevathasan, W., *et al.* Pedunculopontine nucleus deep brain stimulation in Parkinson's disease: A clinical review. *Movement Disorders* **33**, 10-20 (2018).

Supplementary Table 1: Extended Statistical Report

Statistical report for all Main Figures (1 to 6) and Extended Data Figures (1 to 10)

Figure	Data	Normality test (Shapiro-Wilk)	Comparison	Statistical approach	Exact *p* value (unless <0.0001)	Critical values	Multiple comparisons / post-hoc test		Mean difference	95 % CI of difference	Exact p value (adjusted) (unless <0.0001)
**Figure 1c**	Chx10⁺ neuron density in PPN (neurons/mm²)<br>N = 3 mice<br>(4 different coronal levels)	Pass	Chx10⁺ neuron density across the 4 coronal levels	RM one-way ANOVA<br>Geisser-Greenhouse correction	0.0185	F (DFn, DFd)<br>F (1.379, 2.758) = 24.35	Tukey's multiple comparisons test	-4.96 vs. -4.72	52.28	-72.64 to 177.2	0.2374
								-4.96 vs. -4.48	-152.2	-470.3 to 165.9	0.1908
								-4.96 vs. -4.24	-201.5	-350.9 to -52.12	0.0280
								-4.72 vs. -4.48	-204.5	-402.5 to -6.490	0.0470
								-4.72 vs. -4.24	-253.8	-466.7 to -40.91	0.0356
								-4.48 vs. -4.24	-49.32	-398.2 to 299.5	0.7770
**Figure 1c**	ChAT⁺ neuron density in PPN (neurons/mm²)<br>N = 3 mice<br>(4 different coronal levels)	Pass	ChAT⁺ neuron density across the 4 coronal levels	RM one-way ANOVA<br>Geisser-Greenhouse correction	0.0124	F (DFn, DFd)<br>F (1.420, 2.839) = 31.12	Tukey's multiple comparisons test	-4.96 vs. -4.72	94.99	-123.7 to 313.6	0.2236
								-4.96 vs. -4.48	213.0	62.93 to 363.1	0.0254
								-4.96 vs. -4.24	290.3	-58.43 to 639.0	0.0708
								-4.72 vs. -4.48	118.0	42.39 to 193.7	0.0211
								-4.72 vs. -4.24	195.3	-33.89 to 424.5	0.0678
								-4.48 vs. -4.24	77.25	-153.6 to 308.2	0.3327
							Test for linear trend	Slope = -98.89<br>95% CI of slope = -124.0 to -73.78			
**Figure 2c**	Average velocity (m/s) by epoch<br>N = 8 mice<br>(light activation of Chx10-PPN neurons, 1 s)	Pass	Average velocity during light on vs. before<br>(light on – before)	Two-tailed paired t-test	<0.0001	t = 22.24<br>df = 7<br>number of pairs = 8			-0.5721	-0.6329 to -0.5113	
**Figure 2g**	Time active (%) by epoch<br>ChR2-injected group (Chx10-PPN)<br>N = 9 mice	Fail	% of time active within each epoch<br>ChR2 group vs. Control group<br>(ChR2 – Control)	Mann-Whitney test (multiple)<br>Compare ranks for each test<br>Number of tests performed = 3			Mann-Whitney test (multiple)<br>Holm-Sidak method to correct for multiple comparisons	Before<br>(ChR2 – Control)<br>Mann-Whitney U = 10	-6.423<br>----<br>Mean rank difference = -1.556	-20.97 to 8.128<br>----	0.840000
								Light on<br>(ChR2 – Control)<br>Mann-Whitney U = 0	-72.83<br>----<br>Mean rank difference = -6.000	-94.34 to -51.32<br>----	0.027026
	Time active (%) by epoch<br>EYFP-injected control group (Chx10-PPN)<br>N = 3 mice	Pass						After<br>(ChR2 – Control)<br>Mann-Whitney U = 11	-7.945<br>----<br>Mean rank difference = -1.111	-21.99 to 6.101<br>----	0.840000

Figure	Data	Normality test (Shapiro-Wilk)	Comparison	Statistical approach	Exact *p* value (unless <0.0001)	Critical values	Mean difference	95 % CI of difference
**Figure 4g**	Maximum heart rate change (%) compared to baseline<br><br>ChR2-injected group (Chx10-PPN)<br><br>N = 10 mice<br><br>(light activation of Chx10-PPN neurons, 1 s)	Pass	Maximum heart rate change<br><br>Chx10-PPN group vs. Natural group<br><br>(Chx10-PPN – Natural)	Two-tailed unpaired t-test with Welch's correction	0.8102	Welch-corrected<br>t = 0.2433<br>df = 20.69	-0.6524	-6.233 to 4.928
	Maximum heart rate change (%) compared to baseline<br><br>Naturally occurring apneic arrest events<br><br>N = 14 mice	Pass						

Figure	Data	Normality test (Shapiro-Wilk)	Comparison	Statistical approach	Exact *p* value (unless <0.0001)	Critical values	Mean difference	95 % CI of difference
**Figure 5c**	Average velocity (m/s) by epoch<br><br>Chx10-PPN group<br><br>N = 8 mice<br><br>(light activation of Chx10-PPN neurons, 1 s)	Pass	Average velocity before light<br><br>Chx10-PPN group vs. Chx10-vlPAG group<br><br>(Chx10-PPN – Chx10-vlPAG)	Two-tailed Mann-Whitney test	0.0593	Sum of ranks PPN = 45<br>Sum of ranks vlPAG = 60<br>Mann-Whitney U = 9	-0.1340<br><br>----<br><br>Difference between medians = -0.1519	-0.3249 to 0.05690<br><br>----
	Average velocity (m/s) by epoch<br><br>Chx10-vlPAG group<br><br>N = 6 mice<br><br>(light activation of Chx10-vlPAG neurons, 1 s)	Fail						
**Figure 5c**	Average velocity (m/s) by epoch<br><br>Chx10-PPN group<br><br>N = 8 mice<br><br>(light activation of Chx10-PPN neurons, 1 s)	Pass	Average velocity after light<br><br>Chx10-PPN group vs. Chx10-vlPAG group<br><br>(Chx10-PPN – Chx10-vlPAG)	Two-tailed Mann-Whitney test	0.0007	Sum of ranks PPN = 84<br>Sum of ranks vlPAG = 21<br>Mann-Whitney U = 0	0.2150<br><br>----<br><br>Difference between medians = 0.2267	0.09581 to 0.3341<br><br>----
	Average velocity (m/s) by epoch<br><br>Chx10-vlPAG group<br><br>N = 6 mice<br><br>(light activation of Chx10-vlPAG neurons, 1 s)	Fail						

Figure	Data	Normality test (Shapiro-Wilk)	Comparison	Statistical approach	Exact *p* value (unless <0.0001)	Critical values	Mean difference	95 % CI of difference
**Figure 5h**	Maximum respiratory rate change (%) compared to baseline<br><br>Chx10-PPN group<br><br>N = 6 mice<br><br>(light activation of Chx10-PPN neurons, 3 s)	Pass	Maximum respiratory rate change<br><br>Chx10-PPN group vs. Chx10-vlPAG group<br><br>(Chx10-PPN – Chx10-vlPAG)	Two-tailed Mann-Whitney test<br><br>*(for consistency with heart rate comparison)*	0.0022	Sum of ranks PPN = 21<br>Sum of ranks vlPAG = 57<br>Mann-Whitney U = 0	-24.91<br><br>----<br><br>Difference between medians = -23.24	-41.55 to -8.264<br><br>----
	Maximum respiratory rate change (%) compared to baseline<br><br>Chx10-vlPAG group<br><br>N = 6 mice<br><br>(light activation of Chx10-vlPAG neurons, 3 s)	Pass						
**Figure 5h**	Maximum heart rate change (%) compared to baseline<br><br>Chx10-PPN group<br><br>N = 6 mice<br><br>(light activation of Chx10-PPN neurons, 3 s)	Fail	Maximum heart rate change<br><br>Chx10-PPN group vs. Chx10-vlPAG group<br><br>(Chx10-PPN – Chx10-vlPAG)	Two-tailed Mann-Whitney test	0.0260	Sum of ranks PPN = 25<br>Sum of ranks vlPAG = 53<br>Mann-Whitney U = 4	-6.540<br><br>----<br><br>Difference between medians = -5.169	-11.83 to -1.247<br><br>----
	Maximum heart rate change (%) compared to baseline<br><br>Chx10-vlPAG group<br><br>N = 6 mice<br><br>(light activation of Chx10-vlPAG neurons, 3 s)	Pass						

Figure	Data	Normality test (Shapiro-Wilk)	Comparison	Statistical approach	Exact *p* value (unless <0.0001)	Critical values	Mean difference	95 % CI of difference
**Extended data Figure 2f**	Chx10-tdTomato⁺ cell count in PPN<br><br>EYFP-injected control group (Chx10-PPN)<br><br>N = 8 mice	Pass	Chx10-tdTomato⁺ cell counts<br><br>Control group vs. Casp3 group<br><br>(Control – Casp3)	Two-tailed unpaired t-test with Welch's correction	<0.0001	Welch-corrected<br>t = 13.42<br>df = 11.29	286.0	239.3 to 332.7
	Chx10-tdTomato⁺ cell count in PPN<br><br>Casp3-injected group (Chx10-PPN)<br><br>N = 8 mice	Pass						
**Extended data Figure 2h**	Change in the number of arrest events compared to baseline (% change)<br><br>EYFP-injected control group (Chx10-PPN)<br><br>N = 8 mice	Pass	Arrest events (percent change from baseline)<br><br>Control group vs. Casp3 group<br><br>(Control – Casp3)	Two-tailed unpaired t-test with Welch's correction	0.0039	Welch-corrected<br>t = 3.494<br>df = 13.25	72.72	27.84 to 117.6
	Change in the number of arrest events compared to baseline (% change)<br><br>Casp3-injected group (Chx10-PPN)<br><br>N = 8 mice	Pass						

Figure	Data	Normality test (Shapiro-Wilk)	Comparison	Statistical approach	Exact *p* value (unless <0.0001)	Critical values	Multiple comparisons / post-hoc test		Mean difference	95 % CI of difference	Exact p value (adjusted) (unless <0.0001)
**Extended data Figure 6d**	Average respiratory rate within each epoch around 1 s of blue light stimulation<br><br>N = 10 mice	Pass	Average respiratory rate across the 3 epochs	RM one-way ANOVA<br><br>Geisser-Greenhouse correction	<0.0001	F (DFn, DFd)<br>F (1.344, 12.10) = 165.8	Tukey's multiple comparisons test	Before vs. Light on	3.714	2.863 to 4.566	<0.0001
								Before vs. After	-0.2381	-0.6485 to 0.1723	0.2869
								Light on vs. After	-3.953	-4.654 to -3.251	<0.0001
**Extended data Figure 6d**	Average respiratory rate within each epoch around 1 s of yellow light<br><br>N = 10 mice	Pass	Average respiratory rate across the 3 epochs	RM one-way ANOVA<br><br>Geisser-Greenhouse correction	0.2368	F (DFn, DFd)<br>F (1.318, 11.86) = 1.604	Tukey's multiple comparisons test	Before vs. Light on	0.2246	-0.2200 to 0.6691	0.3761
								Before vs. After	-0.04189	-0.3179 to 0.2341	0.9067
								Light on vs. After	-0.2665	-0.8365 to 0.3036	0.4273
**Extended data Figure 6d**	Average heart rate within each epoch around 1 s of blue light stimulation<br><br>N = 10 mice	Pass	Average heart rate across the 3 epochs	RM one-way ANOVA<br><br>Geisser-Greenhouse correction	0.0005	F (DFn, DFd)<br>F (1.555, 13.99) = 15.71	Tukey's multiple comparisons test	Before vs. Light on	40.63	17.02 to 64.23	0.0025
								Before vs. After	-1.234	-18.70 to 16.23	0.9788
								Light on vs. After	-41.86	-70.61 to -13.11	0.0071
**Extended data Figure 6d**	Average heart rate within each epoch around 1 s of yellow light<br><br>N = 10 mice	Pass	Average heart rate across the 3 epochs	RM one-way ANOVA<br><br>Geisser-Greenhouse correction	0.3921	F (DFn, DFd)<br>F (1.434, 12.91) = 0.9184	Tukey's multiple comparisons test	Before vs. Light on	-6.099	-23.27 to 11.07	0.5998
								Before vs. After	-7.370	-17.72 to 2.980	0.1707
								Light on vs. After	-1.271	-20.98 to 18.44	0.9823

Figure	Data	Normality test (Shapiro-Wilk)	Comparison	Statistical approach	Exact *p* value (unless <0.0001)	Critical values	Multiple comparisons / post-hoc test		Mean difference	95 % CI of difference	Exact p value (adjusted) (unless <0.0001)
**Extended data Figure 6d**	Average respiratory rate within each epoch around 3 s of blue light stimulation<br><br>N = 6 mice	Pass	Average respiratory rate across the 3 epochs	RM one-way ANOVA<br><br>Geisser-Greenhouse correction	<0.0001	F (DFn, DFd)<br>F (1.609, 8.045) = 136.0	Tukey's multiple comparisons test	Before vs. Light on	3.150	2.206 to 4.094	0.0003
								Before vs. After	-0.8165	-1.408 to -0.2251	0.0147
								Light on vs. After	-3.966	-4.865 to -3.067	<0.0001
**Extended data Figure 6d**	Average respiratory rate within each epoch around 3 s of yellow light<br><br>N = 6 mice	Pass	Average respiratory rate across the 3 epochs	RM one-way ANOVA<br><br>Geisser-Greenhouse correction	0.1340	F (DFn, DFd)<br>F (1.167, 5.837) = 3.007	Tukey's multiple comparisons test	Before vs. Light on	0.6945	-0.5837 to 1.973	0.2711
								Before vs. After	0.1846	-0.3813 to 0.7506	0.5750
								Light on vs. After	-0.5098	-1.393 to 0.3732	0.2385
**Extended data Figure 6d**	Average heart rate within each epoch around 3 s of blue light stimulation<br><br>N = 6 mice	Pass	Average heart rate across the 3 epochs	RM one-way ANOVA<br><br>Geisser-Greenhouse correction	0.0013	F (DFn, DFd)<br>F (1.082, 5.412) = 35.83	Tukey's multiple comparisons test	Before vs. Light on	85.86	65.86 to 105.8	<0.0001
								Before vs. After	-28.28	-74.49 to 17.93	0.2093
								Light on vs. After	-114.1	-175.2 to -53.08	0.0040
**Extended data Figure 6d**	Average heart rate within each epoch around 3 s of yellow light<br><br>N = 6 mice	Pass	Average heart rate across the 3 epochs	RM one-way ANOVA<br><br>Geisser-Greenhouse correction	0.2150	F (DFn, DFd)<br>F (1.645, 8.223) = 1.862	Tukey's multiple comparisons test	Before vs. Light on	11.73	-15.72 to 39.18	0.4127
								Before vs. After	-6.242	-43.47 to 30.98	0.8531
								Light on vs. After	-17.97	-44.44 to 8.500	0.1624

Figure	Data	Normality test (Shapiro-Wilk)	Comparison	Statistical approach	Exact *p* value (unless <0.0001)	Critical values	Mean difference	95 % CI of difference
**Extended data Figure 6f**	Time spent in preferred chamber (%) before and after conditioning<br><br>Conditioned with blue light<br><br>N = 6 mice	Pass	% of time spent in preferred chamber after conditioning vs. before conditioning<br><br>(Post – Pre)	Two-tailed paired t-test	0.3959	t = 0.9282<br>df = 5	-2.224	-8.382 to 3.934
**Extended data Figure 6f**	Time spent in preferred chamber (%) before and after conditioning<br><br>Conditioned with yellow light<br><br>N = 5 mice	Pass	% of time spent in preferred chamber after conditioning vs. before conditioning<br><br>(Post – Pre)	Two-tailed paired t-test	0.1614	t = 1.716<br>df = 4	-6.441	-16.86 to 3.982
**Extended data Figure 8g**	Time spent in preferred chamber (%) before and after conditioning<br><br>Conditioned with blue light<br><br>N = 6 mice	Fail	% of time spent in preferred chamber after conditioning vs. before conditioning<br><br>(Post – Pre)	Two-tailed Wilcoxon matched-pairs signed rank test	0.0938	Sum of signed ranks (W) = -17.00	-15.56	-40.62 to 9.492

Supplementary Video Descriptions

Supplementary Video 1

Arrest of spontaneous locomotion in the linear corridor upon activation of Chx10-PPN neurons with blue light (1 s train at 40 Hz, 10 ms pulse width). Three examples. Playback speed 0.1x.

Supplementary Video 2

Arrest of other motor behaviors in a cylindrical arena upon activation of Chx10-PPN neurons with blue light (3 s train at 40 Hz, 10 ms pulse width). Three grooming examples, three rearing examples, and two ambulation examples. Playback speed 1x (real-time).

Supplementary Video 3

Pause-and-play pattern observable upon activation of Chx10-PPN neurons with blue light (3 s train at 40 Hz, 10 ms pulse width) during rearing and grooming. For each behavior, two examples are shown: first a ChR2-injected mouse and then an EYFP-injected control mouse. Playback speed 0.6x.

Supplementary Video 4

Arrest of spontaneous locomotion in the linear corridor upon activation of Chx10-vlPAG neurons with blue light (1 s train at 40 Hz, 10 ms pulse width). Two examples. The apparent mass over the lower back is an implanted wireless ECG sensor. Playback speed 0.1x.
