# Supplementary material for: Pedunculopontine Chx10+ neurons control global motor arrest in mice
Source: Nat Neurosci. 2023 Jul 27;26(9):1516–28. doi: 10.1038/s41593-023-01396-3 (PMC10471498; doi:10.1038/s41593-023-01396-3)
Supplement: Supplementary file 2 — Reporting Summary [file 41593_2023_1396_MOESM2_ESM.pdf]

nature portfolio

Corresponding author(s): Ole Kiehn

Last updated by author(s): Jun 19, 2023

Reporting Summary

Nature Portfolio wishes to improve the reproducibility of the work that we publish. This form provides structure for consistency and transparency in reporting. For further information on Nature Portfolio policies, see our Editorial Policies and the Editorial Policy Checklist.

Statistics

For all statistical analyses, confirm that the following items are present in the figure legend, table legend, main text, or Methods section.

n/a	Confirmed	
☐	☒	The exact sample size (*n*) for each experimental group/condition, given as a discrete number and unit of measurement
☐	☒	A statement on whether measurements were taken from distinct samples or whether the same sample was measured repeatedly
☐	☒	The statistical test(s) used AND whether they are one- or two-sided<br>*Only common tests should be described solely by name; describe more complex techniques in the Methods section.*
☒	☐	A description of all covariates tested
☐	☒	A description of any assumptions or corrections, such as tests of normality and adjustment for multiple comparisons
☐	☒	A full description of the statistical parameters including central tendency (e.g. means) or other basic estimates (e.g. regression coefficient) AND variation (e.g. standard deviation) or associated estimates of uncertainty (e.g. confidence intervals)
☐	☒	For null hypothesis testing, the test statistic (e.g. *F*, *t*, *r*) with confidence intervals, effect sizes, degrees of freedom and *P* value noted<br>*Give P values as exact values whenever suitable.*
☒	☐	For Bayesian analysis, information on the choice of priors and Markov chain Monte Carlo settings
☒	☐	For hierarchical and complex designs, identification of the appropriate level for tests and full reporting of outcomes
☒	☐	Estimates of effect sizes (e.g. Cohen's *d*, Pearson's *r*), indicating how they were calculated

Our web collection on statistics for biologists contains articles on many of the points above.

Software and code

Policy information about availability of computer code

Data collection	Most behavior data (video capture) was collected using Ethovision XT (v.15, Noldus Information Technology) (same software for control and acquisition of the behavioral recording sessions).<br>Video capture in the linear corridor was performed using the software of the behavioral apparatus (TSE Motion software, v.8.5.8, TSE Systems)(MotoRater 303030 series, TSE Systems).<br>EMG data was acquired using AxoScope (10.6, Molecular Devices).<br>ECG and whole-body plethysmography signals were acquired with IOX software (v. 2.10.5, Emka Technologies).<br>Microscopy images were acquired using Zeiss microscopes equipped with the Zen Blue software (Zeiss).
Data analysis	Ethovision XT (v.15, Noldus Information Technology) was used for most behavior data tracking.<br>The bottom and side views of the data recorded in the linear corridor were tracked with TSE Motion software (v.8.5.8, TSE Systems). The bottom view was also tracked using DeepLabCut (v.2.1.8.2) for analyzing lymb dynamics with custom-made scripts.<br>EMG data was analyzed using Spike2 (v.7.06, Cambridge Electronic Design).<br>ECG and whole-body plethysmography data was analyzed with custom written Python scripts (v. 3.7.4)(see Methods for details).<br>Most microscopy images were pre-processed, analyzed, and exported using Zen Blue 3.1/3.2/3.6, or Zen Lite 2.5/3.6 (Zeiss).<br>Some images (see methods) were analyzed using the Fiji distribution of ImageJ (ImageJ2 2.9.0; Java 1.8.0_322)<br>RNAscope analysis was performed using Imaris (v.9.7.2; Bitplane, Oxford Instruments)<br>Custom made scripts were written using Python 3.7 or 3.8, and standard Python libraries for scientific computing (Matplotlib (v. 3.3.2), NumPy (v. 1.19.2), pandas (v. 1.2.1), SciPy (v. 1.6.0), Seaborn (v. 0.11.2)). The sequence of the main steps and functions used in custom-made Python scripts, together with the specific libraries and their versions are described in the methods section.<br>All statistical analysis were performed using GraphPad Prism (v. 9.4.1).<br>Data plots were originally generated with custom-made Python scripts (v. 3.7.4) and rendered using standard plotting libraries (Matplotlib v. 3.3.2 and Seaborn v. 0.11.2).<br>In all cases, further aesthetic modifications, editing and figure assembly was done using Adobe Illustrator 2022 (v. 26.5)

Custom code written to analyze limb dynamics is available at https://github.com/kiehnlab/pauseandplay and https://doi.org/10.5281/zenodo.8013911 (DOI: 10.5281/zenodo.8013911). Other custom code is available from the corresponding authors upon request.

For manuscripts utilizing custom algorithms or software that are central to the research but not yet described in published literature, software must be made available to editors and reviewers. We strongly encourage code deposition in a community repository (e.g. GitHub). See the Nature Portfolio guidelines for submitting code & software for further information.

Data

Policy information about availability of data

All manuscripts must include a data availability statement. This statement should provide the following information, where applicable:

- Accession codes, unique identifiers, or web links for publicly available datasets
- A description of any restrictions on data availability
- For clinical datasets or third party data, please ensure that the statement adheres to our policy

Data supporting the main findings presented in this study are available as source data files accompanying this manuscript. Other data and material are available from the corresponding authors upon request.

Field-specific reporting

Please select the one below that is the best fit for your research. If you are not sure, read the appropriate sections before making your selection.

☒ Life sciences ☐ Behavioural & social sciences ☐ Ecological, evolutionary & environmental sciences

For a reference copy of the document with all sections, see nature.com/documents/nr-reporting-summary-flat.pdf

Life sciences study design

All studies must disclose on these points even when the disclosure is negative.

Sample size	No statistical methods were used to predetermine sample sizes but our group sizes were similar to those used in the field. Group sizes were typically smaller for anatomical experiments (3-4 mice) compared to behavioral experiments (6-14 mice). See Caggiano et. al., 2018; Cregg et al., 2020
Data exclusions	In behavior experiments involving optogenetic stimulation all mice were screened for their response to blue light prior to behavioral testing (see Methods for details). Mice that showed no motor phenotype upon blue light stimulation (motor arrest visually assessed by the experimenter) were excluded from behavioral experimental groups before data collection. Post-mortem tissue from mice in behavioral and anatomical experiments was evaluated to assess injection site and fiber placement. Mice that had off-target or no viral expression were excluded from analysis.
Replication	All experiments have both biological and technical replicates. Each experiment was at least replicated in 3 mice and several samples/trials/events were analyzed from each mouse. Group sizes range from 3 and up to 14 mice. The specific number of animals, trials, events, or samples used on an experiment is included in each panel and/or the respective figure legend, the results section, and the specific methods section associated to it.
Randomization	Both male and female mice (approximately 1:1), littermates and non-littermates, were randomly selected and allocated to experimental groups. No protocol was followed for systematic randomization.
Blinding	During behavioral data collection, the experimenter was blind to virus type if two different groups were used as treatment and control (e.g. AAV-FLEX-ChR2 vs. AAV-FLEX-Fluorophore; AAV-FLEX-Casp3 vs. AAV-FLEX-Fluorophore), but was not blind to light wavelength (blue light vs. yellow light) or injection target (PPN vs. vlPAG) if a single group was used as its own control. However, data was analyzed as automated as possible and using the same parameters regardless of group to avoid any influence on the outcome. The experimenters were not blind to virus type or injection target during anatomy experiments (neither for data collection nor analysis).

Reporting for specific materials, systems and methods

We require information from authors about some types of materials, experimental systems and methods used in many studies. Here, indicate whether each material, system or method listed is relevant to your study. If you are not sure if a list item applies to your research, read the appropriate section before selecting a response.

Materials & experimental systems

n/a	Involved in the study	
☐	☒	Antibodies
☒	☐	Eukaryotic cell lines
☒	☐	Palaeontology and archaeology
☐	☒	Animals and other organisms
☒	☐	Human research participants
☒	☐	Clinical data
☒	☐	Dual use research of concern

Methods

n/a	Involved in the study	
☒	☐	ChIP-seq
☒	☐	Flow cytometry
☒	☐	MRI-based neuroimaging

Antibodies

Antibodies used

Rabbit anti-DsRed/tdTomato/mCherry (#632496, Clontech/Takara) - used for staining tdTomato and mCherry
Chicken anti-GFP (#ab13970, Abcam) - used for staining GFP and eYFP
Goat anti-ChAT (#AB144P, Millipore) - used for staining ChAT
Alexa Fluor 488 goat anti-chicken (#A11039 Invitrogen)
Alexa Fluor 488 donkey anti-goat (#A11055, Invitrogen)
Alexa Fluor 568 donkey anti-rabbit (#A10042, Invitrogen)

Validation

All primary antibodies used in this study are validated by the manufacturer for IHC applications in mouse tissue and/or by previous work.
anti-GFP: https://www.abcam.com/gfp-antibody-ab13970.html & https://antibodyregistry.org (AB_300798)
anti-ChAT: https://www.merckmillipore.com/DK/en/product/Anti-Choline-Acetyltransferase-Antibody,MM_NF-AB144P & https://antibodyregistry.org (AB_2079751)
anti-DsRed/tdTomato/mCherry: https://www.takarabio.com/products/antibodies-and-elisa/fluorescent-protein-antibodies/red-fluorescent-protein-antibodies ; https://www.labome.com/product/Takara-Bio-Clontech/632496.html ; & https://antibodyregistry.org (AB_10013483)

Animals and other organisms

Policy information about studies involving animals; ARRIVE guidelines recommended for reporting animal research

Laboratory animals

Laboratory mouse (Mus musculus). Transgenic lines: heterozygous Chx10_Cre alone, or crossed with the homozygous conditional lines R26R_ChR2–EYFP (Stock No: 012569), R26R_EYFP (Stock No: 006148), or R26R_tdTomato (Stock No: 007905). All homozygous conditional lines are from Jackson Laboratories. Specificity and origin of the heterozygous Chx10_Cre line has been described in previous work (see Methods section for reference). All experiments were performed in adult (> 8 weeks) male or female mice (randomly selected, approximately 1:1).

Wild animals

No wild animals were used in this study.

Field-collected samples

No field-collected samples were used in this study.

Ethics oversight

All animal experiments and procedures were carried according to the EU Directive 2010/63/EU and approved by the Danish Animal Experiments Inspectorate (Dyreforsøgstilsynet, Dyreforsøgstilsynet, license no. 2017-15-0201-01172) and the local ethics committee at the University of Copenhagen.

Note that full information on the approval of the study protocol must also be provided in the manuscript.
